# Design, synthesis and biological evaluation of novel artemisinin derivatives as potent P-glycoprotein modulators for overcoming multidrug resistance

**DOI:** 10.1039/d6ra07164c

**Published:** 2026-10-26

**Authors:** Jerónimo Laiolo, Artemis Tsirogianni, Cecilia L. Barbieri, Mariana Belén Joray, Dafni G. Graikioti, Dimitra Toumpa, Jazmín Lavallén, Nicolás A. Rodriguez, D. Mariano A. Vera, Constantinos M. Athanassopoulos, María C. Carpinella

**Affiliations:** a Universidad Católica de Córdoba Córdoba Argentina; b Synthetic Organic Chemistry Laboratory, Department of Chemistry, University of Patras GR-26504 Patras Greece kath@upatras.gr; c Institute of Chemistry and Biochemistry, IQUIBIM, College of Exact and Natural Sciences, Universidad Nacional de Mar del Plata – QUIAMM – INBIOTEC CONICET Mar del Plata Argentina dmavera@yahoo.com; d Fine Chemical and Natural Products Laboratory, CIDIE CONICET-UCC, Universidad Católica de Córdoba Córdoba Argentina ceciliacarpinella@ucc.edu.ar

## Abstract

Multidrug resistance (MDR) mediated by P-glycoprotein (P-gp, ABCB1) remains a major obstacle to successful chemotherapy, underscoring the need for potent and safe agents capable of blocking P-gp function and restoring drug sensitivity. To address this challenge, we synthesized and systematically evaluated a library of novel artemisinin derivatives. Screening identified compounds A7, A12, A13, A14 and A15 as effective molecules, significantly reducing rhodamine 123 (Rho123) efflux at minimum effective concentrations (MECs) ranging from 0.19 to 6 µM. Notably, compounds A16 and A20 displayed the highest potency, with MECs as low as 0.049 µM. These derivatives also efficiently inhibited P-gp-mediated doxorubicin (Dox) efflux, with a MEC value of 0.19 µM for compound A16 and 0.049 µM for compound A20. Consistent with these findings, both compounds markedly enhanced Dox cytotoxicity by sensitizing K562/Dox cells at concentrations of 0.050 and 0.005 µM, respectively. Molecular dynamics simulations and multiscale QM/MM calculations yielded useful clues for understanding the binding modes of the derivatives and identified key structural determinants and key amino acid residues that would be involved in P-gp modulation. The potent P-gp-modulating activity of compounds A16 and A20, together with the absence of cytotoxicity toward normal PBMCs at the nanomolar concentrations at which they modulated P-gp activity, highlights these artemisinin derivatives as promising scaffolds for the development of novel P-gp modulators. Collectively, the computational and experimental findings provide mechanistic insights into P-gp inhibition and establish a framework for the rational design of artemisinin-based P-gp modulators.

## Abstract 2

Screening novel artemisinin derivatives identified A16 and A20 as potent Rho123 efflux inhibitors (MEC: 49 nM), enhancing doxorubicin (Dox) cytotoxicity in K562/Dox cells at 50 and 5 nM, respectively.

## Graphical abstract

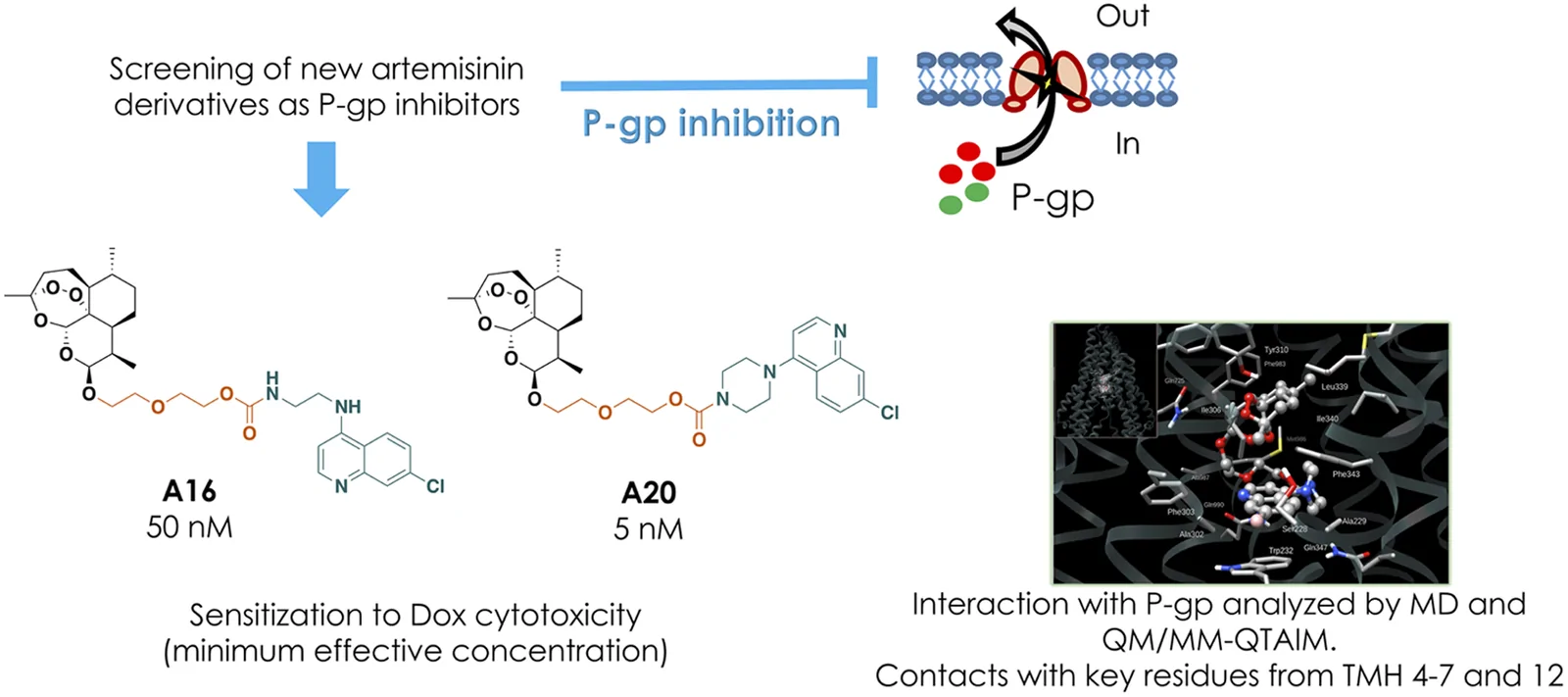

## 1 Introduction

Cancer remains one of the leading causes of mortality worldwide,^1^ representing a major challenge to both human and animal health while imposing a substantial socio-economic burden.^2,3^ Although chemotherapy has significantly improved cancer treatment outcomes,^4^ the emergence of drug resistance continues to compromise its therapeutic efficacy.^5^ Multiple mechanisms contribute to the development of simultaneous resistance to several structurally and functionally unrelated drugs,^6^ a phenomenon known as multidrug resistance (MDR).^7^ Among these mechanisms, increased drug efflux mediated by membrane transporters is considered a major contributor to MDR.^8,9^ Members of the ATP-binding cassette (ABC) transporter family actively transport a broad range of drugs in an ATP-dependent manner, thereby preventing them from reaching their intracellular targets and ultimately reducing therapeutic efficacy, frequently leading to treatment failure.^10^ Among ABC efflux transporters, P-glycoprotein (P-gp, also known as ABCB1 or MDR1) is one of the principal mediators of MDR owing to its remarkable substrate promiscuity. It efficiently extrudes numerous chemotherapeutic agents, including doxorubicin (Dox), vincristine and paclitaxel, as well as a wide variety of endogenous and exogenous compounds.^11,12^ Structurally, P-gp comprises two homologous transmembrane domains (TMDs), each containing six transmembrane helices (TMHs), and one nucleotide-binding domain (NBDs), which are responsible for ATP binding and hydrolysis.^13^ The involvement of P-gp in cancer drug resistance^12^ along with its association with poor clinical prognosis and its overexpression in several malignancies, including leukemia,^14–16^ reinforces the urgent need to identify effective modulators capable of inhibiting its efflux activity. However, despite decades of research, first-, second-, and third-generation P-gp inhibitors have achieved limited clinical success, mainly due to toxicity, pharmacokinetic interactions, insufficient transporter selectivity, and modest therapeutic efficacy.^17^ These limitations have prompted the development of, fourth-generation inhibitors, including natural-product-derived compounds, as promising candidates for more effective and selective P-gp modulation^18–20^ Despite extensive efforts to translate P-gp inhibition into anticancer therapy, the clinical development of P-gp inhibitors has remained challenging, with no inhibitor achieving approval specifically for overcoming P-gp-mediated chemotherapy resistance.^21,22^ Since its discovery, artemisinin has become one of the most important antimalarial drugs^23^ and has served as a privileged scaffold for the development of numerous derivatives with improved pharmacological properties.^24^ Although initially developed for malaria treatment,^25,26^ these derivatives have demonstrated a broad range of biological activities, including antiparasitic, anti-inflammatory, dermatological and anticancer effects^25,27,28^ including activity against hematologic malignancies.^29^ Importantly, several studies have demonstrated that artemisinin derivatives can partially overcome P-gp-mediated MDR in different cell lines.^30^ Their well-defined and readily modifiable scaffold, together with the ability of synthetic derivatives to incorporate hydrophobic and electron-rich substituents and appropriate linkers, makes artemisinin an attractive platform for the rational optimization of P-gp modulators. Artemisinin, artesunate and dihydroartemisinin moderately enhanced intracellular Dox accumulation and toxicity in P-gp-overexpressing K562/Adr cells.^31^ Likewise, several synthetic artemisinin derivatives have been reported to impair P-gp function to different extents, as demonstrated by functional assays across a variety of cellular models.^32–36^

These findings provide a strong rationale for exploring artemisinin derivatives as P-gp modulators. Accordingly, a library of artemisinin derivatives was rationally designed and synthesized based on current knowledge of the structural requirements for P-gp inhibition and the key residues involved in ligand recognition within the TMHs. These pharmacophoric features include a high hydrophobicity, an appropriate linker, and an electron-rich aromatic moiety, suitable substituents, appropriate linker flexibility and a favorable molecular shape.^20,37^

Based on these considerations, a series of 21 artemisinin derivatives was rationally designed, synthesized, and biologically evaluated to identify novel agents capable of modulating P-gp-mediated drug resistance. The most active compounds were further characterized using molecular dynamics simulations and multiscale QM/MM calculations to elucidate their binding modes and identify the molecular determinants of P-gp inhibition.

## 2 Experimental

### 2.1 Chemistry

Compounds A1 to A20 (Fig. 1) were synthesized according to the chemical reactions described in Schemes 1 and 2.

**Fig. 1:**
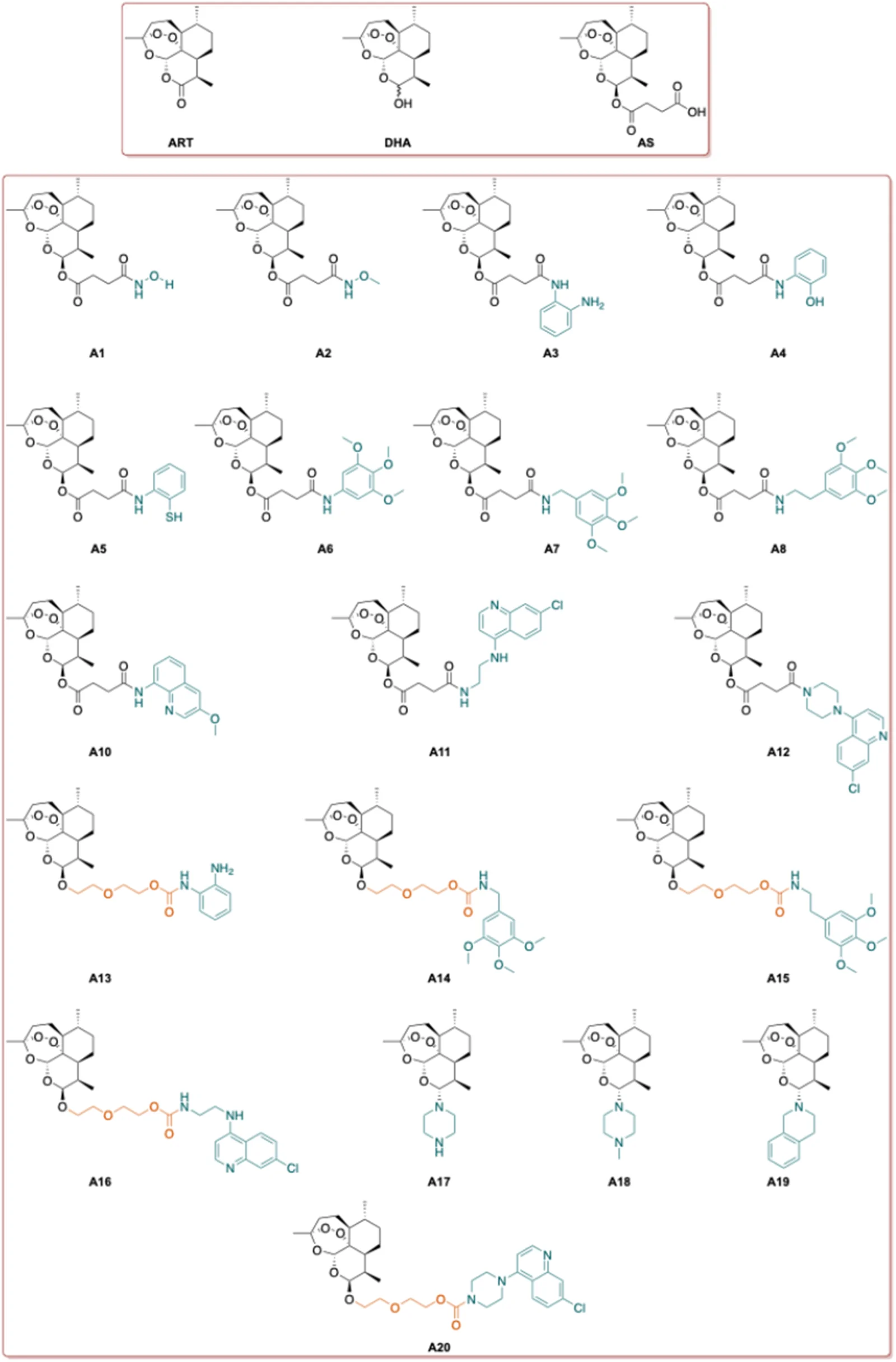
Chemical structures of the artemisinin derivatives A1–A20.

**Scheme 1:**
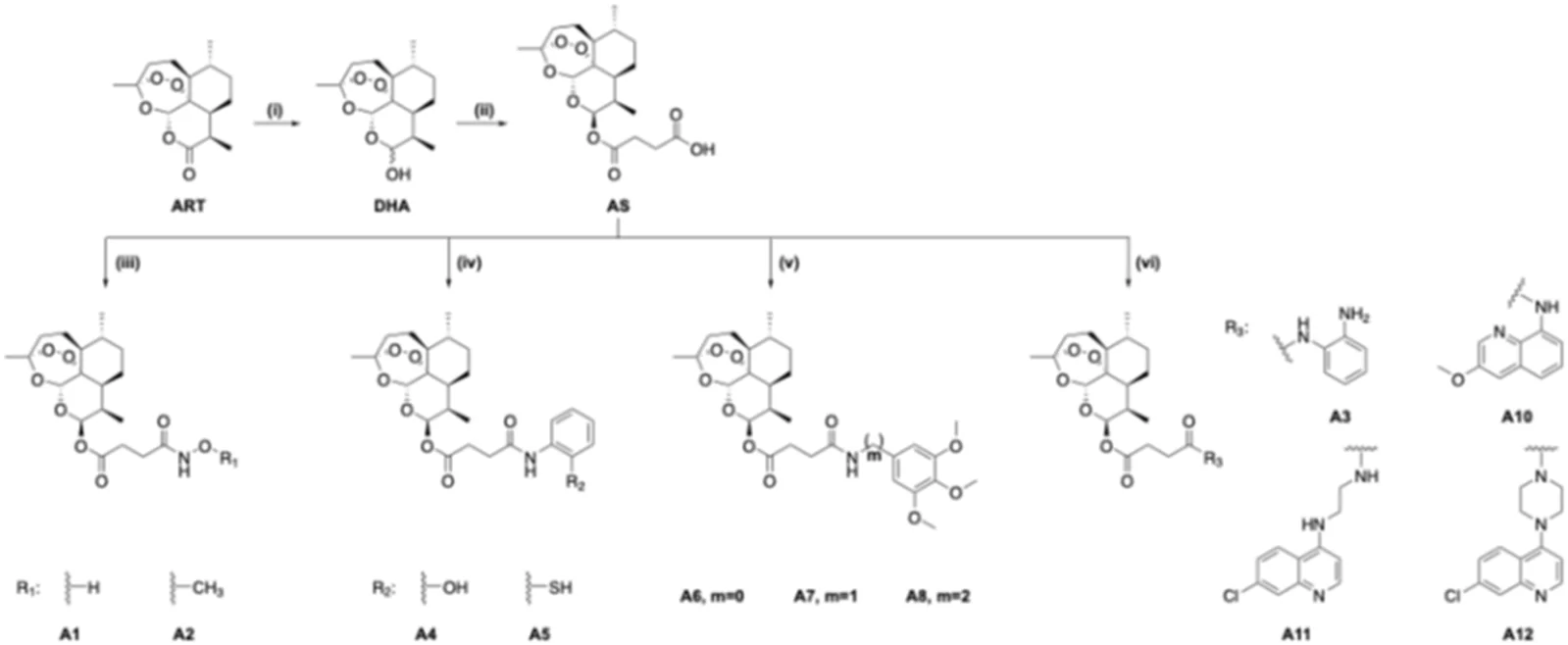
Synthesis of the artemisinin derivatives A1 to A12; reagents and conditions: (i) NaBH_4_, MeOH, 0 °C, 2 h, 95%; (ii) succinic anhydride, dichloromethane (DCM), 4-(dimethylamino)pyridine (DMAP), 0–25 °C, 24 h, 90%; (iii) corresponding hydroxylamine, (((*Z*)-(1-cyano-2-ethoxy-2-oxoethylidene)amino)oxy)-*N*,*N*-dimethyl(morpholin-4-yl)methaniminium hexafluorophosphate (COMU), *N*,*N*-diisopropylethylamine (DIPEA), DCM, 3 h, 0–25 °C, 32–38%; (iv) corresponding aniline, 1-hydroxybenzotriazole (HOBt), ethyldimethylaminopropyl carbodiimide hydrochloride (EDCI), DCM, overnight, under Ar, rt, 40–70%; (v) corresponding amine, *O*-(benzotriazol-1-yl)-*N*,*N*,*N*′,*N*′-tetramethyluronium hexafluorophosphate (HBTU), DIPEA, dimethylformamide (DMF), under Ar, 0–25 °C, 24 h, 50–70%; (vi) corresponding amine, HBTU, DIPEA, DMF, under Ar, 0–25 °C, overnight, 45–67%.

**Scheme 2:**
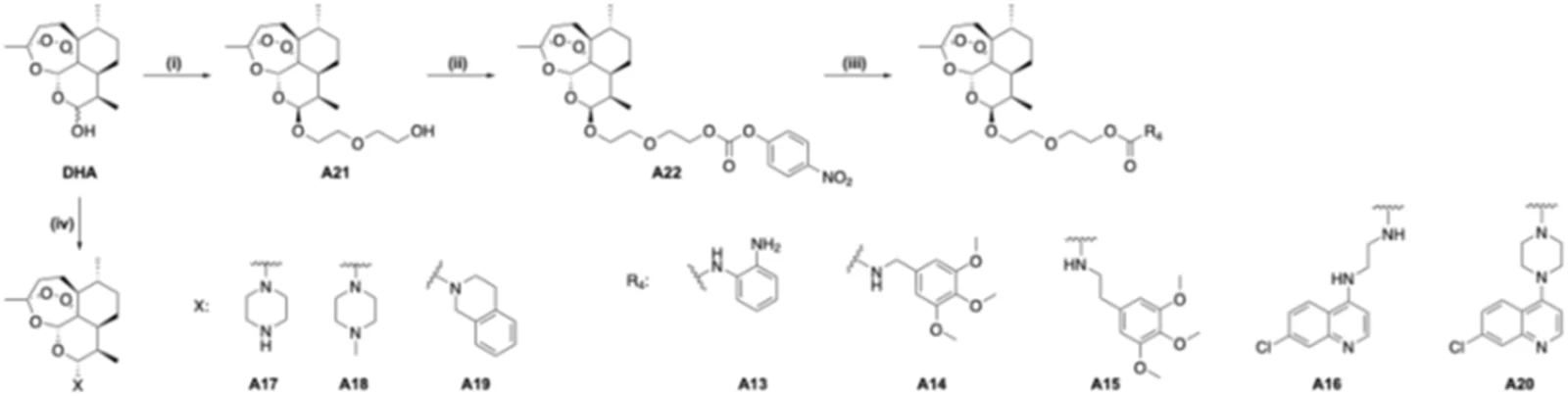
Synthesis of the artemisinin derivatives A13 to A20; reagents and conditions: (i) diethylene glycol, BH_3_·Et_2_O, DCM, 0–25 °C, 2 h, 72%; (ii) 4-nitrophenyl chloroformate, 0–25 °C, 2 h, 80%; (iii) corresponding amine, DIPEA, DCM, 0–25 °C, 24 h, 50–61%; (iv) corresponding amine, dimethyl sulfoxide (DMSO), oxalyl chloride, xylene, DIPEA, DCM, 0–25 °C, 24 h, 40–45%.

#### 2.1.1. Materials, chemicals and instruments

All solvents were dried and purified prior to use according to standard procedures. When required, reactions were performed under inert atmosphere (Ar) in preflamed glassware. Anhydrous Na_2_SO_4_ was used for drying solutions, and the solvents were then routinely removed at *ca.* 40 °C under reduced pressure. All reagents employed in the present work were commercially available and used without further purification. The synthesis of DHA and artesunate was carried out according to a previously published procedure.^38,39^ The synthesis of *N*^1^-(7-chloroquinolin-4-yl)ethane-1,2-diamine and 7-chloro-4-(piperazin-1-yl)quinoline was performed according to published procedures.^40^ Flash column chromatography (FCC) was performed on silica gel (70–230 and 230–400 mesh, Merck, Germany) and analytical thin layer chromatography (TLC) on silica 60gel-F_254_ precoated aluminum foils (0.2 mm film, Merck, Germany). Spots on the TLC plates were visualized with UV light at 254 nm and using anisaldehyde solution. ^1^H NMR spectra were recorded in CDCl_3_ at 600.13 MHz and ^13^C spectra at 150.9 MHz on a Bruker AVANCEIII HD spectrometer (see Fig. S1–S38). Chemical shifts (*δ*) are referenced with respect to the residual CHCl_3_ proton of the solvent CDCl_3_ at *δ* = 7.26 ppm. ^13^C NMR spectra were fully decoupled and are referenced to the middle peak of the solvent CDCl_3_ at *δ* = 77.0 ppm. Splitting patterns are designated as: s, singlet; d, doublet; t, triplet; q, quadruplet; dd, double doublet, *etc.* Coupling constants (*J*) are reported in Hertz. Electrospray Ionization High-Resolution Mass Spectrometry spectra (ESI-HRMS spectra) were recorded at 30 eV on a HESI-ORBITRAP MS, EXPLORIS 120 (Thermo Fischer Scientific, BRE725531, Waltham, MA, USA) using MeOH as the solvent. ESI^+^ mass spectra were recorded at 30 V, on a Micromass-Platform LC spectrometer using MeOH as solvent. The purity of the tested compounds was determined by reversed phase high performance liquid chromatography (HPLC) (UHPLC – Diode Array) equipped with a UHPLC – DAD, Dionex Ultimate 300, Thermo Scientific and a Thermo Acclaim C18 (100 × 2.1 mm, 2.2 µm) column. The purity of the tested compounds, which corresponded to >95% (see the SI), was evaluated as a percentage ratio between the areas of the main peak and of possible impurities.

#### 2.1.2. Experimental procedures

##### 2.1.2.1 General procedure A for the synthesis of hydroxamic acid A1 and hydroxamate A2

To a cold solution (0 °C) of the hydroxylamine (1 eq.) and DIPEA (0.8 eq.), for pH 7–7.5, in DCM (0.11 M) were added artesunate (0.89 eq.), COMU (1 eq.) and DIPEA (0.89 eq.). The reaction mixture was stirred at room temperature for 3 h. After completion of the reaction (monitored by TLC) the mixture was evaporated to dryness under reduced pressure and the residue was diluted with EtOAc. The mixture was then washed with H_2_O and brine, dried over Na_2_SO_4_, filtered, and evaporated to dryness under reduced pressure. The residue was subjected to FCC, affording the pure products A1 and A2 as colorless oils.

Compound A1: artesunate was reacted with NH_2_OH·HCl according to general procedure A. Yield = 38%; colorless oil; *R*_f_ (PhMe/EtOAc 1 : 1) = 0.34; ^1^H NMR (CDCl_3_, 600 MHz) *δ* 6.19 (s, 1H), 5.30 (s, 1H), 3.42 (t, *J* = 6.6 Hz, 2H), 3.00 (s, 1H), 2.88 (s, 3H), 2.71 (d, *J* = 8.4 Hz, 1H), 2.47 (t, *J* = 7.8 Hz, 2H), 2.43–2.35 (m, 2H), 2.11–2.03 (m, 6H), 1.72–1.64 (m, 2H), 1.59 (s, 2H), 1.43 (s, 3H), 1.26 (s, 3H) ppm; ^13^C NMR (CDCl_3_, 151 MHz) *δ* 182.3, 135.2, 108.3, 104.7, 104.6, 89.9, 79.1, 51.6, 44.6, 37.7, 36.4, 34.3, 30.2, 26.0, 24.6, 20.4, 18.0, 16.3 ppm; HR-ESI (*m*/*z*) [M^+^] calculated for C_19_H_29_NO_8_ 399.1893. Found: 399.1899; HPLC analysis: flow rate: 0.3 mL min^−1^, peak area 95.5%, *t*_R_ = 5.4 min, gradient: 10% CH_3_CN for 1 min, 10% to 95% CH_3_CN over 4.5 min, 95% CH_3_CN for 2 min, 95% to 10% CH_3_CN over 0.5 min and 10% CH_3_CN for 2 min.

Compound A2: artesunate was reacted with MeONH_2_·HCl according to general procedure A. Yield = 32%; colorless oil; *R*_f_ (PhMe/EtOAc 1 : 1) = 0.42; ^1^H NMR (CDCl_3_, 600 MHz) *δ* 8.79 (br s, 1H), 5.76 (d, *J* = 9.9 Hz, 1H), 5.43 (s, 1H), 3.75 (s, 3H), 2.77 (m, 3H), 2.55 (m, 1H), 2.36 (m, 2H), 2.02 (dt, *J* = 14.6, 4.4 Hz, 1H), 1.88 (ddt, *J* = 13.7, 6.7, 3.6 Hz, 1H), 1.77 (dq, *J* = 13.5, 3.8 Hz, 1H), 1.71 (dq, *J* = 13.4, 3.4 Hz, 1H), 1.61 (dt, *J* = 13.8, 4.6 Hz, 1H), 1.47 (m, 1H), 1.42 (s, 3H), 1.35 (m, 2H), 1.28 (dd, *J* = 11.3, 6.4 Hz, 1H), 1.01 (td, *J* = 12.7, 3.6 Hz, 1H), 0.95 (d, *J* = 6.2 Hz, 3H), 0.85 (d, *J* = 7.1 Hz, 3H) ppm; ^13^C NMR (CDCl_3_, 151 MHz) *δ* 171.73, 171.10, 104.65, 92.51, 91.65, 80.26, 64.83, 51.65, 45.32, 37.41, 36.33, 34.19, 31.85, 29.64, 26.03, 25.63, 24.70, 22.10, 20.33, 12.17 ppm; HR-ESI (*m*/*z*) [M^+^] calculated for C_20_H_31_NO_8_ 413.2050. Found: 413.2055; HPLC analysis: flow rate: 0.3 mL min^−1^, peak area 97.7%, *t*_R_ = 5.8 min, gradient: 90% CH_3_CN for 2 min, 90% to 100% CH_3_CN over 4 min, 100% CH_3_CN for 2 min, 100% to 90% CH_3_CN over 0.1 min and 90% CH_3_CN for 2 min.

##### 2.1.2.2 General procedure B for the synthesis of amides A4 and A5

To a solution of artesunate (1 eq.) in DCM (0.04 M), the corresponding amine (1.2 eq.), HOBt (1.5 eq.), and EDCI·HCl (1.1 eq.) were added. The reaction mixture was stirred at room temperature overnight under an Ar atmosphere. After the completion of the reaction (monitored by TLC), the mixture was diluted with DCM, and washed with cold 5% aq. citric acid, 5% aq. NaHCO_3_, H_2_O and brine, dried over Na_2_SO_4_, filtered, and evaporated to dryness under reduced pressure. The residue was subjected to FCC, affording the pure artesunate amides A4 and A5 as oils.

Compound A4: artesunate was reacted with *o*-aminophenol according to general procedure B. Yield = 40%; orange oil; *R*_f_ (PhMe/EtOAc 8 : 2) = 0.13; ^1^H NMR (CDCl_3_, 600 MHz) *δ* 8.68 (br s, 1H), 8.23 (s, 1H), 7.18–7.09 (m, 3H), 6.99 (d, *J* = 7.8 Hz, 1H), 6.86 (t, *J* = 7.8 Hz, 1H), 5.77 (d, *J* = 9.6 Hz, 1H), 5.44 (s, 1H), 2.83–2.69 (m, 4H), 2.57 (br s, 1H), 2.04 (d, *J* = 14.4 Hz, 1H), 1.91–1.89 (m, 1H), 1.76–1.60 (m, 6H), 1.42 (s, 3H), 1.34–1.26 (m, 4H), 0.96 (d, *J* = 5.4 Hz, 3H), 0.88 (d, *J* = 7.2 Hz, 3H) ppm; ^13^C NMR (CDCl_3_, 151 MHz) *δ* 171.7, 148.4, 129.0, 128.1, 127.0, 122.4, 120.4, 119.6, 104.6, 92.5, 91.6, 80.1, 51.5, 45.1, 37.2, 36.2, 34.0, 31.7, 31.2, 29.7, 25.8, 24.5, 21.9, 20.1, 11.9 ppm; HR-ESI (*m*/*z*) [M^+^] calculated for C_25_H_33_NO_8_ 475.2206. Found: 475.2209; HPLC analysis: flow rate: 0.3 mL min^−1^, peak area 99.1%, *t*_R_ = 5.6 min, gradient: 30% CH_3_CN for 1 min, 30% to 100% CH_3_CN over 4.5 min, 100% CH_3_CN for 2 min, 100% to 30% CH_3_CN over 0.5 min and 30% CH_3_CN for 2 min.

Compound A5: artesunate was reacted with 2-aminothiophenol according to general procedure B. Yield = 70%; brown oil; *R*_f_ (PhMe/EtOAc 9 : 1) = 0.38; ^1^H NMR (CDCl_3_, 600 MHz) *δ* 7.45 (d, *J* = 7.8 Hz, 1H), 7.17–7.14 (m, 2H), 6.73 (d, *J* = 7.2 Hz, 1H), 6.60 (t, *J* = 7.8 Hz, 1H) 5.80 (d, *J* = 10.2 Hz, 1H), 5.43 (s, 1H), 4.15 (q, *J* = 7.2 Hz, 2H), 2.74–2.56 (m, 5H), 2.40–2.35 (m, 1H), 2.04–2.01 (m, 1H), 1.91–1.87 (m, 2H), 1.79–1.70 (m, 2H), 1.63–1.61 (m, 1H), 1.43 (s, 3H), 1.38–1.30 (m, 2H), 1.27–1.22 (m, 4H), 0.96 (d, *J* = 6.0 Hz, 3H), 0.86 (d, *J* = 7.2 Hz, 3H) ppm; ^13^C NMR (CDCl_3_, 151 MHz) *δ* 171.1, 136.8, 131.5, 128.6, 127.9, 127.6, 127.2, 118.3, 115.3, 104.4, 92.1, 91.5, 80.1, 60.7, 51.5, 45.2, 37.2, 36.2, 34.0, 34.8, 29.2, 28.9, 25.9, 21.9, 20.1, 14.1, 12.0 ppm; HR-ESI (*m*/*z*) [M^+^] calculated for C_25_H_33_NO_7_S 491.1978. Found: 491.1979; HPLC analysis: flow rate: 0.5 mL min^−1^, peak area 99.8%, *t*_R_ = 6.0 min, gradient: 30% CH_3_CN for 1 min, 30% to 100% CH_3_CN over 4.5 min, 100% CH_3_CN for 2.5 min, 100% to 30% CH_3_CN over 0.1 min and 30% CH_3_CN for 2 min.

##### 2.1.2.3 General procedure C for the synthesis of artesunate amides A3, A6, A7, A8, A10, A11 and A12

To a stirring solution of artesunate in dry DMF (0.5 M), under Ar atmosphere, the corresponding amine (1.5 eq.) and HBTU (1.1 eq.) were added, and the suspension was cooled to 0 °C. Subsequently DIPEA (4 eq.) was added dropwise and the reaction mixture was stirred for 5 min at 0 °C and then at room temperature overnight. Upon consumption of the starting material, the reaction mixture was diluted with EtOAc, and was washed with cold NaHCO_3_, H_2_O and brine. The organic layer was dried over Na_2_SO_4_, filtered, and evaporated to dryness under reduced pressure. The residue was subjected to FCC, to afford the pure artesunate amides A3–A12 as pale-yellow oils.

Compound A3: artesunate was reacted with *o*-phenylenediamine according to general procedure C. Yield = 45%; colorless oil; *R*_f_ (PhMe/EtOAc 8 : 2) = 0.11; ^1^H NMR (CDCl_3_, 600 MHz) *δ* 7.30 (m, 2H), 5.60 (s, 1H), 5.39 (s, 1H), 5.30 (d, *J* = 3.5 Hz, 1H), 4.75 (d, *J* = 9.1 Hz, 1H), 2.62 (m, 1H), 2.38 (m, 1H), 2.31 (m, 1H), 2.03 (dtd, *J* = 14.3, 4.7, 3.0 Hz, 1H), 1.88 (m, 2H), 1.80 (m, 2H), 1.67 (m, 2H), 1.56 (dt, *J* = 13.8, 4.5 Hz, 1H), 1.50 (ddd, *J* = 13.3, 11.6, 5.0 Hz, 2H), 1.43 (d, *J* = 5.1 Hz, 3H), 1.36 (m, 1H), 1.27 (m, 2H), 1.01 (m, 1H), 0.95 (m, 6H), 0.91 (m, 1H) ppm; ^13^C NMR (CDCl_3_, 151 MHz) *δ* 158.8, 135.3, 129.4, 125.6, 108.5, 104.8, 96.7, 95.1, 91.6, 90.0, 88.1, 81.5, 63.7, 52.8, 45.8, 44.8, 37.8, 36.6, 35.1, 31.1, 30.3, 26.2, 25.0, 22.4, 20.6, 16.5, 14.3, 13.1 ppm; HR-ESI (*m*/*z*) [M^+^] calculated for C_25_H_34_N_2_O_7_ 474.2366. Found: 474.2360; HPLC analysis: flow rate: 0.5 mL min^−1^, peak area 100%, *t*_R_ = 5.8 min, gradient: 30% CH_3_CN for 1 min, 30% to 100% CH_3_CN over 4 min, 100% CH_3_CN for 2 min, 100% to 30% CH_3_CN over 0.1 min and 30% CH_3_CN for 3 min.

Compound A6: artesunate was reacted with 3,4,5-trimethoxy aniline according to the general procedure C. Yield = 50%; pale-yellow oil; *R*_f_ (PhMe/EtOAc 7 : 3) = 0.14; ^1^H NMR (CDCl_3_, 600 MHz) *δ* 7.68 (s, 1H), 7.24 (d, *J* = 7.5 Hz, 1H), 7.17 (d, *J* = 7.4 Hz, 1H), 6.87 (s, 2H), 6.48 (s, 2H), 5.78 (d, *J* = 9.9 Hz, 1H), 5.44 (s, 1H), 3.84 (d, *J* = 7.7 Hz, 6H), 3.80 (s, 3H), 2.90 (s, 3H), 2.81 (s, 2H), 2.35 (s, 2H), 2.05–2.01 (m, 1H), 1.76 (dq, *J* = 13.5, 3.2 Hz, 1H), 1.71 (dq, *J* = 13.5, 3.2 Hz, 1H), 1.64–1.60 (m, 1H), 1.41 (s, 3H), 1.25 (s, 1H), 0.96 (d, *J* = 6.1 Hz, 3H), 0.84 (d, *J* = 7.1 Hz, 3H) ppm; ^13^C NMR (CDCl_3_, 151 MHz) *δ* 176.3, 153.6, 153.2, 129.0, 127.3, 125.3, 104.5, 104.2, 97.5, 92.4, 91.5, 80.1, 60.9, 56.2, 51.5, 45.2, 38.6, 37.3, 36.2, 34.0, 31.9, 29.6, 28.4, 25.9, 24.6, 22.0, 20.2, 12.0 ppm; ESI-MS (30 eV) *m*/*z*: 572.63 [M + Na]^+^; HR-ESI (*m*/*z*) [M + Na]^+^ calculated for C_28_H_39_NNaO_10_ 572.2472. Found: 572.2458; HPLC analysis: flow rate: 0.5 mL min^−1^, peak area 98.1%, *t*_R_ = 7.3 min, gradient: 30% CH_3_CN for 1 min, 30% to 100% CH_3_CN over 4.5 min, 100% CH_3_CN for 2.5 min, 100% to 30% CH_3_CN over 0.1 min and 30% CH_3_CN for 2 min.

Compound A7: artesunate was reacted with 3,4,5-trimethoxy benzylamine according to the general procedure C. Yield = 70%; pale-yellow oil; *R*_f_ (PhMe/EtOAc 1 : 1) = 0.16; ^1^H NMR (CDCl_3_, 600 MHz) *δ* 7.24 (d, *J* = 7.4 Hz, 1H), 7.17 (d, *J* = 7.4 Hz, 1H), 6.51 (s, 2H), 6.02 (t, *J* = 5.1 Hz, 1H), 5.75 (d, *J* = 9.8 Hz, 1H), 5.39 (s, 1H), 4.42–4.38 (m, 1H), 4.36–4.32 (m, 1H), 3.85 (s, 6H), 3.82 (s, 3H), 2.86–2.82 (m, 1H), 2.79–2.74 (m, 1H), 2.59–2.53 (m, 2H), 2.51–2.46 (m, 1H), 2.35 (s, 3H), 2.04–2.00 (m, 1H), 1.78–1.75 (m, 1H), 1.73–1.70 (m, 1H), 1.63–1.59 (m, 1H), 1.41 (s, 3H), 1.35–1.32 (m, 1H), 0.96 (d, *J* = 6.1 Hz, 3H), 0.83 (d, *J* = 7.1 Hz, 3H) ppm; ^13^C NMR (CDCl_3_, 151 MHz) *δ* 171.8, 171.1, 153.4, 137.2, 134.0, 129.0, 128.2, 125.3, 104.8, 104.5, 92.2, 91.5, 80.1, 60.8, 56.1, 51.5, 45.2, 43.9, 37.3, 36.2, 34.1, 31.7, 30.9, 29.7, 25.9, 24.6, 22.0, 20.2, 12.0 ppm; ESI-MS (30 eV) *m*/*z*: 586.68 [M + Na]^+^; HR-ESI (*m*/*z*) [M + Na]^+^ calculated for C_29_H_41_NNaO_10_ 586.2628. Found: 586.2619; HPLC analysis: flow rate: 0.5 mL min^−1^, peak area 95.1%, *t*_R_ = 5.5 min, gradient: 30% CH_3_CN for 1 min, 30% to 100% CH_3_CN over 4 min, 100% CH_3_CN for 2 min, 100% to 30% CH_3_CN over 0.1 min and 30% CH_3_CN for 3 min.

Compound A8: artesunate was reacted with 2-(3,4,5-trimethoxyphenyl) ethanamine according to general procedure C. Yield = 50%; pale-yellow oil; *R*_f_ (PhMe/EtOAc 1 : 1) = 0.25; ^1^H NMR (CDCl_3_, 600 MHz) *δ* 6.41 (d, *J* = 8.4 Hz, 2H), 6.23–6.05 (m, 1H), 5.61–5.35 (m, 1H), 3.84 (d, *J* = 6.0 Hz, 6H), 3.81 (d, *J* = 3.9 Hz, 3H), 3.51–3.47 (m, 2H), 2.81 (s, 3H), 2.74 (q, *J* = 7.0 Hz, 2H), 2.68–2.64 (m, 2H), 2.49–2.32 (m, 3H), 2.07–2.00 (m, 1H), 1.90–1.86 (m, 1H), 1.72–1.65 (m, 1H), 1.59–1.45 (m, 2H), 1.40 (m, 3H), 1.29–1.22 (m, 2H), 0.98–0.84 (m, 6H) ppm; ^13^C NMR (CDCl_3_, 151 MHz) *δ* 175.7, 172.5, 153.3, 136.6, 135.0, 134.4, 128.2, 108.1, 105.6, 104.5, 89.7, 79.0, 60.8, 56.1, 51.4, 44.4, 40.9, 38.7, 37.5, 36.2, 35.8, 34.1, 30.7, 30.0, 29.7, 25.8, 24.4, 20.2, 16.2 ppm; ESI-MS (30 eV) *m*/*z*: 600.72 [M + Na]^+^; HR-ESI (*m*/*z*) [M^+^] calculated for C_30_H_43_NO_10_ 577.2887. Found: 577.2881; HPLC analysis: flow rate: 0.3 mL min^−1^, peak area 95.6%, *t*_R_ = 5.7 min, gradient: 30% CH_3_CN for 1 min, 30% to 100% CH_3_CN over 4.5 min, 100% CH_3_CN for 2 min, 100% to 30% CH_3_CN over 0.5 min and 30% CH_3_CN for 2 min.

Compound A10: artesunate was reacted with 3-methoxyquinolin-8-amine according to general procedure C. Yield = 67%; colorless oil; *R*_f_ (PhMe/EtOAc 9 : 1) = 0.12; ^1^H NMR (CDCl_3_, 600 MHz) *δ* 9.84 (s, 1H), 8.64 (dd, *J* = 4.2, 1.6 Hz, 1H), 8.48 (d, *J* = 2.7 Hz, 1H), 8.02 (dd, *J* = 8.3, 1.6 Hz, 1H), 7.39 (dd, *J* = 8.3, 4.2 Hz, 1H), 6.78 (d, *J* = 2.7 Hz, 1H), 5.82 (d, *J* = 9.8 Hz, 1H), 5.43 (s, 1H), 3.91 (s, 3H), 2.91 (m, 4H), 2.58 (m, 1H), 2.03 (ddd, *J* = 14.6, 4.9, 3.0 Hz, 1H), 1.88 (ddt, *J* = 13.6, 6.9, 3.6 Hz, 1H), 1.76 (dq, *J* = 13.6, 3.8 Hz, 1H), 1.70 (dq, *J* = 13.4, 3.3 Hz, 1H), 1.61 (tt, *J* = 9.2, 4.6 Hz, 2H), 1.49 (ddd, *J* = 13.6, 11.4, 5.0 Hz, 1H), 1.44 (s, 3H), 1.37 (td, *J* = 13.5, 3.5 Hz, 1H), 1.30 (m, 2H), 1.01 (td, *J* = 12.7, 12.1, 3.5 Hz, 1H), 0.95 (d, *J* = 6.1 Hz, 3H), 0.85 (d, *J* = 7.1 Hz, 3H) ppm; ^13^C NMR (CDCl_3_, 151 MHz) *δ* 171.5, 169.7, 158.5, 135.3, 135.0, 129.0, 128.2, 125.3, 122.0, 104.4, 99.8, 92.2, 91.5, 80.1, 55.6, 51.6, 45.3, 37.3, 36.2, 34.1, 32.1, 31.8, 29.4, 26.0, 24.6, 22.0, 20.2, 12.0 ppm; HR-ESI (*m*/*z*) [M + Na]^+^ calculated for C_29_H_36_N_2_NaO_8_ 563.2369. Found: 563.2363; HPLC analysis: flow rate: 0.5 mL min^−1^, peak area 96.6%, *t*_R_ = 3.4 min, gradient: 90% CH_3_CN for 1 min, 90% to 100% CH_3_CN over 4 min, 100% CH_3_CN for 3 min, 100% to 90% CH_3_CN over 0.1 min and 90% CH_3_CN for 2 min.

Compound A11: artesunate was reacted with *N*^1^-(7-chloroquinolin-4-yl)ethane-1,2-diamine according to general procedure C. Yield = 48%; colorless oil; *R*_f_ (DCE/MeOH 9 : 1) = 0.10; ^1^H NMR (CDCl_3_, 600 MHz) *δ* 8.40 (d, *J* = 5.6 Hz, 1H), 7.97 (d, *J* = 9.0 Hz, 1H), 7.94 (d, *J* = 2.1 Hz, 1H), 7.39 (dd, *J* = 9.0, 2.1 Hz, 1H), 7.00 (dd, *J* = 13.7, 7.8 Hz, 1H), 6.29 (d, *J* = 5.7 Hz, 1H), 5.59 (d, *J* = 9.8 Hz, 1H), 4.99 (s, 1H), 4.11 (q, *J* = 7.1 Hz, 1H), 3.70 (m, 1H), 3.58 (m, 1H), 3.48 (ddt, *J* = 11.8, 7.6, 3.5 Hz, 1H), 3.43 (m, 1H), 2.76 (m, 2H), 2.60 (dt, *J* = 14.2, 7.0 Hz, 1H), 2.53 (m, 1H), 2.46 (m, 1H), 2.30 (m, 1H), 2.14 (m, 1H), 1.96 (ddd, *J* = 14.6, 4.9, 3.1 Hz, 1H), 1.83 (ddt, *J* = 13.6, 6.6, 3.5 Hz, 1H), 1.65 (dp, *J* = 10.5, 3.4 Hz, 1H), 1.52 (dt, *J* = 13.7, 4.5 Hz, 1H), 1.34 (s, 3H), 1.30 (ddd, *J* = 13.8, 11.9, 4.9 Hz, 1H), 1.25 (t, *J* = 7.2 Hz, 1H), 1.19 (m, 1H), 1.10 (m, 1H), 1.03 (td, *J* = 13.3, 3.2 Hz, 1H), 0.93 (d, *J* = 6.2 Hz, 3H), 0.74 (d, *J* = 7.1 Hz, 3H) ppm; ^13^C NMR (CDCl_3_, 151 MHz) *δ* 174.1, 172.4, 151.5, 150.3, 136.1, 126.1, 123.1, 117.3, 104.8, 98.5, 92.8, 91.7, 80.3, 60.7, 51.6, 45.3, 44.9, 38.7, 37.5, 36.4, 34.3, 31.9, 31.3, 30.9, 26.1, 24.8, 22.1, 20.4, 14.5, 12.3 ppm; HR-ESI (*m*/*z*) [M + Na]^+^ calculated for C_30_H_38_ClN_3_NaO_7_ 610.2296. Found: 610.2284; HPLC analysis: flow rate: 0.5 mL min^−1^, peak area 99.9%, *t*_R_ = 2.0 min, gradient: 90% CH_3_CN for 1 min, 90% to 100% CH_3_CN over 4 min, 100% CH_3_CN for 3 min, 100% to 90% CH_3_CN over 0.1 min and 90% CH_3_CN for 2 min.

Compound A12: artesunate was reacted with 7-chloro-4-(piperazin-1-yl)quinoline according to general procedure C. Yield = 61%; colorless oil; *R*_f_ (DCE/MeOH 9 : 1) = 0.10; ^1^H NMR (CDCl_3_, 600 MHz) *δ* 8.73 (d, *J* = 5.0 Hz, 1H), 8.05 (d, *J* = 2.1 Hz, 1H), 7.95 (d, *J* = 9.0 Hz, 1H), 7.46 (dd, *J* = 9.0, 2.2 Hz, 1H), 6.84 (d, *J* = 5.0 Hz, 1H), 5.80 (d, *J* = 9.8 Hz, 1H), 5.43 (s, 1H), 3.89 (d, *J* = 5.6 Hz, 2H), 3.79 (br s, 2H), 3.23 (q, *J* = 3.8 Hz, 2H), 3.18 (d, *J* = 5.3 Hz, 2H), 2.84 (m, 1H), 2.79 (s, 3H), 2.76 (m, 1H), 2.66 (m, 1H), 2.57 (dqd, *J* = 14.3, 7.1, 4.5 Hz, 1H), 2.02 (ddd, *J* = 14.6, 4.9, 3.0 Hz, 1H), 1.89 (ddt, *J* = 13.7, 6.9, 3.8 Hz, 1H), 1.77 (dd, *J* = 13.6, 4.0 Hz, 1H), 1.71 (dd, *J* = 13.4, 3.3 Hz, 1H), 1.62 (dt, *J* = 13.9, 4.5 Hz, 1H), 1.47 (m, 1H), 1.41 (s, 3H), 1.37 (dd, *J* = 13.6, 3.6 Hz, 1H), 1.28 (m, 1H), 1.01 (m, 1H), 0.96 (d, *J* = 6.1 Hz, 3H), 0.87 (d, *J* = 7.2 Hz, 3H) ppm; ^13^C NMR (CDCl_3_, 151 MHz) *δ* 171.7, 169.9, 156.4, 151.9, 150.1, 135.2, 129.0, 128.2, 126.6, 125.3, 124.8, 121.8, 109.3, 104.5, 92.1, 91.5, 80.1, 51.6, 45.4, 41.7, 38.6, 37.3, 36.2, 34.1, 31.9, 29.5, 27.6, 26.0, 24.6, 22.0, 20.2, 12.1 ppm; HR-ESI (*m*/*z*) [M + H]^+^ calculated for C_32_H_41_ClN_3_O_7_ 614.2633. Found 614.2620 and [M + Na]^+^ calculated for C_32_H_40_ClN_3_NaO_7_ 636.2452. Found: 636.2439; HPLC analysis: flow rate: 0.5 mL min^−1^, peak area 98.2%, *t*_R_ = 4.3 min, gradient: 30% CH_3_CN for 1 min, 30% to 100% CH_3_CN over 4.5 min, 100% CH_3_CN for 2 min, 100% to 30% CH_3_CN over 0.5 min and 30% CH_3_CN for 2 min.

##### 2.1.2.4 General procedure for the synthesis of artemisinin carbamates A13–A16 and A20

To an ice-cold (0 °C) solution of activated alcohol A22 (1 eq.) in DCM (0.3 M), corresponding amine (1.1 eq.) was added, followed by the dropwise addition of DIPEA (1.5 eq.). The reaction mixture was stirred at room temperature for 24 h. After the completion of the reaction, the mixture was diluted with DCE and was washed with NaHCO_3_, H_2_O and brine. The organic layer was dried over Na_2_SO_4_, filtered, and evaporated to dryness under reduced pressure. The residue was subjected to FCC, to afford the pure artesunate carbamates as a pale-yellow and yellow oils.

Compound A13: compound A22 was reacted with *o*-phenylenediamine according to general procedure. Yield = 35%; pale-yellow oil; *R*_f_ (PhMe/EtOAc 2 : 8) = 0.13; ^1^H NMR (CDCl_3_, 600 MHz) *δ* 7.01 (m, 1H), 6.78 (m, 1H), 5.49 (s, 1H), 4.84 (d, *J* = 3.4 Hz, 2H), 4.42–4.20 (m, 2H), 3.99–3.91 (m, 1H), 3.76–3.70 (m, 3H), 3.69–3.64 (m, 2H), 3.62–3.57 (m, 2H), 2.66–2.62 (m, 1H), 2.41–2.33 (m, 1H), 2.05–2.01 (m, 1H), 1.90–1.85 (m, 1H), 1.83–1.74 (m, 2H), 1.65–1.62 (m, 2H), 1.59–1.56 (m, 3H), 1.48–1.44 (m, 2H), 1.44–1.44 (m, 3H), 0.94 (m, 3H), 0.90 (m, 3H) ppm; ^13^C NMR (CDCl_3_, 151 MHz) *δ* 104.2, 102.3, 87.9, 81.1, 72.2, 70.7, 67.7, 61.9, 52.6, 44.5, 37.5, 36.4, 34.6, 30.9, 26.1, 24.4, 20.4, 13.0 ppm; ESI-MS (30 eV) *m*/*z*: 529.3 (M + Na)^+^; HR-ESI (*m*/*z*) [M^+^] calculated for C_26_H_38_N_2_O_8_ 506.2628. Found: 506.2630; HPLC analysis: flow rate: 0.5 mL min^−1^, peak area 98.2%, *t*_R_ = 5.3 min, gradient: 30% CH_3_CN for 1 min, 30% to 100% CH_3_CN over 4.5 min, 100% CH_3_CN for 2 min, 100% to 30% CH_3_CN over 0.5 min and 30% CH_3_CN for 2 min.

Compound A14: compound A22 was reacted with 3,4,5-trimethoxy benzylamine according to general procedure. Yield = 50%; pale-yellow oil; *R*_f_ (PhMe/EtOAc 8 : 2) = 0.13; ^1^H NMR (CDCl_3_, 600 MHz) *δ* 6.51 (s, 2H), 5.47 (s, 1H), 5.33–5.27 (m, 1H), 4.83 (d, *J* = 3.3 Hz, 1H), 4.32–4.27 (t, *J* = 5 Hz, 3H), 4.21–4.18 (m, 1H), 3.95–3.93 (m, 1H), 3.85 (s, 6H), 3.82 (s, 3H), 3.69 (t, *J* = 4.7 Hz, 2H), 3.63–3.60 (m, 2H), 2.62–2.58 (m, 1H), 2.37–2.32 (m, 2H), 1.96–1.93 (m, 1H), 1.86–1.78 (m, 2H), 1.63–1.60 (m, 2H), 1.48–1.41 (m, 2H), 1.37 (s, 3H), 1.25 (s, 2H), 0.94 (d, *J* = 6.4 Hz, 3H), 0.90 (d, *J* = 7.4 Hz, 3H) ppm; ^13^C NMR (CDCl_3_, 151 MHz) *δ* 180.3, 156.4, 153.4, 137.4, 134.1, 127.7, 104.6, 104.1, 102.1, 87.9, 81.1, 77.2, 70.5, 69., 67.32, 64.4, 60.8, 56.1, 52.5, 45.4, 44.5, 37.5, 37.4, 36.4, 34.7, 30.8, 29.7, 26.1, 24.4, 20.3, 12.9 ppm; ESI-MS (30 eV) *m*/*z*: 618.45 [M + Na]^+^; HR-ESI (*m*/*z*) [M + Na]^+^ calculated for C_30_H_45_NNaO_11_ 618.2890. Found: 618.2880; HPLC analysis: flow rate: 0.5 mL min^−1^, peak area 95.3%, *t*_R_ = 3.4 min, gradient: 50% CH_3_CN for 1 min, 50% to 100% CH_3_CN over 4 min, 100% CH_3_CN for 2 min, 100% to 50% CH_3_CN over 0.1 min and 50% CH_3_CN for 3 min.

Compound A15: compound A22 was reacted with 2-(3,4,5-trimethoxyphenyl) ethanamine according to general procedure. Yield = 61%; pale-yellow oil; *R*_f_ (PhMe/EtOAc 9 : 1) = 0.38; ^1^H NMR (CDCl_3_, 600 MHz) *δ* 6.40 (s, 2H), 5.44 (s, 1H), 4.82 (d, *J* = 3.3 Hz, 1H), 4.25–4.21 (m, 1H), 4.19–4.15 (m, 1H), 3.84 (s, 6H), 3.82 (s, 3H), 3.67–3.65 (m, 3H), 3.63–3.59 (m, 2H), 3.43 (q, *J* = 6.7 Hz, 2H), 2.75 (t, *J* = 7.1 Hz, 2H), 2.63–2.58 (m, 1H), 2.38–2.33 (m, 3H), 2.01–1.98 (m, 1H), 1.89–1.84 (m, 1H), 1.80 (td, *J* = 13.6, 3.6 Hz, 1H), 1.74–1.70 (m, 1H), 1.63–1.59 (m, 1H), 1.46–1.43 (m, 1H), 1.41 (s, 3H), 1.25–1.21 (m, 3H), 0.94 (d, *J* = 6.3 Hz, 3H), 0.90 (d, *J* = 7.4 Hz, 3H) ppm; ^13^C NMR (CDCl_3_, 151 MHz) *δ* 156.3, 153.4, 129.0, 128.2, 125.3, 105.6, 104.0, 102.1, 87.9, 81.1, 70,5, 69.4, 67.3, 64.1, 60.8, 56.2, 52.5, 44.4, 42.2, 37.4, 36.4, 34.7, 26.1, 24.7, 24.4, 20.3, 12.9 ppm; ESI-MS (30 eV) *m*/*z*: 632.39 [M + Na]^+^; HR-ESI (*m*/*z*) [M + Na]^+^ calculated for C_31_H_47_NNaO_11_ 632.3047. Found: 632.3038; HPLC analysis: flow rate: 0.5 mL min^−1^, peak area 100%, *t*_R_ = 6.1 min, gradient: 30% CH_3_CN for 1 min, 30% to 100% CH_3_CN over 4 min, 100% CH_3_CN for 2 min, 100% to 30% CH_3_CN over 0.1 min and 30% CH_3_CN for 3 min.

Compound A16: compound A22 was reacted with *N*^1^-(7-chloroquinolin-4-yl)ethane-1,2-diamine according to general procedure. Yield = 60%; yellow oil; *R*_f_ (CH_2_Cl_2_/MeOH 93 : 7) = 0.28; ^1^H NMR (CDCl_3_, 600 MHz) *δ* 8.47 (d, *J* = 5.5 Hz, 1H), 7.95 (s, 1H), 7.83 (d, *J* = 8.9 Hz, 1H), 7.37 (d, *J* = 8.9, 1H), 6.64 (br s, 1H), 6.33 (d, *J* = 5.5 Hz, 1H), 5.76 (br s, 1H), 5.45 (s, 1H), 4.82 (d, *J* = 3.5 Hz, 1H), 4.33–4.30 (m, 1H), 4.22–4.17 (m, 1H), 3.78 (t, *J* = 5.8 Hz, 4H), 3.67 (t, *J* = 4.6 Hz, 2H), 3.64 (t, *J* = 5.8 Hz, 4H), 3.58–3.54 (m, 2H), 3.43 (t, *J* = 5.3 Hz, 1H), 2.59–2.56 (m, 1H), 2.39–2.33 (m, 1H), 2.02–1.99 (ddd, *J* = 14.6, 5.0, 3.0 Hz, 1H), 1.88–1.83 (ddt, *J* = 13.6, 6.7, 3.6 Hz, 1H), 1.77 (td, *J* = 13.5, 3.5 Hz, 1H), 1.71 (m, 1H), 1.61–1.57 (dq, *J* = 13.1, 3.3 Hz, 1H), 1.41 (s, 3H), 0.92 (d, *J* = 6.3 Hz, 3H), 0.85 (d, *J* = 7.4 Hz, 3H) ppm; ^13^C NMR (CDCl_3_, 151 MHz) *δ* 158.7, 150.9, 150.6, 135.4, 127.6, 125.7, 122.0, 117.0, 104.1, 101.9, 98.3, 87.9, 81.2, 71.3, 70.7, 69.1, 66.9, 66.1, 64.8, 63.7, 61.7, 61.2, 52.5, 45.6, 44.4, 42.6, 39.8, 37.5, 36.4, 34.6, 30.8, 26.2, 24.7, 24.4, 20.3, 12.9 ppm; ESI-MS (30 eV) *m*/*z*: 642.32 [M + Na]^+^; HR-ESI (*m*/*z*) [M + H]^+^ calculated for C_31_H_43_ClN_3_O_8_ 620.2739. Found: 620.2733; HPLC analysis: flow rate: 0.5 mL min^−1^, peak area 95.1%, *t*_R_ = 4.3 min, gradient: 30% CH_3_CN for 1 min, 30% to 100% CH_3_CN over 4.5 min, 100% CH_3_CN for 2.5 min, 100% to 30% CH_3_CN over 0.1 min and 30% CH_3_CN for 2 min.

Compound A20: compound A22 was reacted with 7-chloro-4-(piperazin-1-yl)quinoline according to general procedure. Yield = 60%; yellow oil; *R*_f_ (PhMe/EtOAc 4 : 6) = 0.12; ^1^H NMR (CDCl_3_, 600 MHz) *δ* 8.73 (d, *J* = 5.0 Hz, 1H), 8.06 (d, *J* = 2.1 Hz, 1H), 7.94 (d, *J* = 8.9 Hz, 1H), 7.44 (d, *J* = 2.1 Hz, 1H), 6.84 (d, *J* = 5.0 Hz, 1H), 5.42 (s, 1H), 4.85 (d, *J* = 3.5 Hz, 1H), 4.27 (t, *J* = 4.8 Hz, 2H), 3.96–3.92 (m, 1H), 3.78–3.77 (m, 4H), 3.74–3.71 (m, 2H), 3.65–3.62 (m, 2H), 3.52 (s, 1H), 3.19 (t, *J* = 5.1 Hz, 2H), 2.65–2.60 (m, 1H), 2.39–2.32 (m, 1H), 2.16 (s, 3H), 2.03–1.98 (ddd, *J* = 14.6, 5.0, 3.0 Hz, 1H), 1.89–1.84 (ddt, *J* = 14.0, 7.1, 3.6 Hz, 1H), 1.81 (td, *J* = 13.6, 3.6 Hz, 1H), 1.74 (dq, *J* = 14.2, 3.9 Hz, 1H), 1.62 (dt, *J* = 13.2, 3.4 Hz, 1H), 1.49–1.43 (m, 1H), 1.41 (s, 3H), 1.24 (m, 2H), 0.94 (d, *J* = 6.3 Hz, 3H), 0.91 (d, *J* = 7.4 Hz, 3H) ppm; ^13^C NMR (CDCl_3_, 151 MHz) *δ* 156.8, 155.3, 151.9, 150.0, 135.2, 128.7, 126.6, 125.6, 124.8, 109.3, 104.1, 102.1, 87.9, 81.1, 71.3, 70.5, 69.4, 67.2, 65.0, 63.7, 52.2, 52.0, 44.4, 42.6, 37.5, 36.4, 34.7, 30.8, 26.2, 24.4, 20.4, 13.0 ppm; HR-ESI (*m*/*z*) [M + H]^+^ calculated for C_33_H_45_ClN_3_O_8_ 646.2895. Found: 646.2886 and [M + Na]^+^ calculated for C_33_H_44_ClN_3_NaO_8_ 668.2715. Found: 668.2705; HPLC analysis: flow rate: 0.5 mL min^−1^, peak area 95.6%, *t*_R_ = 4.7 min, gradient: 30% CH_3_CN for 1 min, 30% to 100% CH_3_CN over 4.5 min, 100% CH_3_CN for 2.5 min, 100% to 30% CH_3_CN over 0.1 min and 30% CH_3_CN for 2 min.

##### 2.1.2.5 General procedure for the synthesis of 10-aza-artemisinin derivatives A17, A18 and A19

To an ice-cold (0 °C) solution of DHA (1 eq.) and DMSO (0.010 mL) in dry xylene (0.2 M), oxalyl-chloride (1.1 eq.) was added dropwise and the reaction mixture was stirred at room temperature for 10 min under Ar conditions. Then, a solution of a corresponding amine (4 eq.) and DIPEA (1.1 eq.) in dry CH_2_Cl_2_ (0.2 M) was added dropwise and the reaction mixture was stirred at room temperature overnight. After the completion of the reaction, the mixture was diluted with EtOAc; sequentially washed with water, 5% aqueous Na_2_CO_3_, 5% aqueous NaHCO_3_, water, and brine; dried over anhydrous Na_2_SO_4_; and concentrated to dryness under reduced pressure. The residues thus obtained were subjected to FCC purification affording the conjugate A17–A19 as a colorless or pale-yellow oils.

Compound A17: DHA was reacted with piperazine according to general procedure. Yield = 42%; pale-yellow oil; *R*_f_ (CH_2_Cl_2_/MeOH/Et_3_N 10 : 1 : 0.1) = 0.10; ^1^H NMR (CDCl_3_, 600 MHz) *δ* 5.25 (s, 1H), 4.02 (d, *J* = 10.2 Hz, 1H), 3.22–3.16 (m, 6H), 3.09 (q, *J* = 7.3 Hz, 8H), 3.02–2.98 (m, 2H), 2.53–2.48 (m, 1H), 2.35–2.29 (m, 1H), 1.99–1.95 (m, 1H), 1.84 (ddt, *J* = 13.6, 6.7, 3.5 Hz, 1H), 1.75–1.67 (m, 2H), 0.93 (d, *J* = 6.3 Hz, 3H), 0.85 (d, *J* = 6.4 Hz, 1H), 0.76 (d, *J* = 7.1 Hz, 3H) ppm; ^13^C NMR (CDCl_3_, 151 MHz) *δ* 104.1, 91.4, 90.9, 80.1, 51.6, 45.7, 43.5, 37.4, 36.3, 34.2, 28.5, 25.9, 24.7, 21.6, 20.2, 13.4, 8.6 ppm; HR-ESI (*m*/*z*) [M + H]^+^ calculated for C_19_H_33_N_2_O_4_ 353.2440. Found: 353.2431; HPLC analysis: flow rate: 0.3 mL min^−1^, peak area 96.1%, *t*_R_ = 7.2 min, gradient: 30% CH_3_CN for 1 min, 30% to 100% CH_3_CN over 4.5 min, 100% CH_3_CN for 2 min, 100% to 30% CH_3_CN over 0.5 min and 30% CH_3_CN for 2 min.

Compound A18: DHA was reacted with *N*-methylpiperazine according to general procedure. Yield = 45%; colorless oil; *R*_f_ (PhMe/EtOAc 97 : 3) = 0.13; ^1^H NMR (CDCl_3_, 600 MHz) *δ* 5.25 (s, 1H), 4.05 (d, *J* = 10.2 Hz, 1H), 3.11–3.08 (m, 2H), 2.92–2.89 (m, 2H), 2.69–2.66 (m, 4H), 2.58–2.51 (m, 1H), 2.42 (s, 3H), 2.37–2.27 (m, 1H), 2.00–1.96 (m, 1H), 1.87–1.82 (m, 1H), 1.71–1.66 (m, 2H), 1.54–1.50 (m, 1H), 1.48–1.42 (m, 1H), 1.34 (s, 3H), 1.32–1.27 (m, 2H), 1.25–1.19 (m, 2H), 1.02–0.97 (m, 1H), 0.93 (d, *J* = 6.3 Hz, 3H), 0.78 (d, *J* = 7.2 Hz, 3H) ppm; ^13^C NMR (CDCl_3_, 151 MHz) *δ* 103.9, 91.4, 90.7, 80.3, 54.6, 51.6, 45.8, 45.3, 37.4, 36.3, 34.3, 28.6, 25.9, 24.8, 21.6, 20.3, 13.4 ppm; ESI-MS (30 eV) *m*/*z*: 389.5 [M + Na]^+^, 405.3 [M + K]^+^; HR-ESI (*m*/*z*) [M + H]^+^ calculated for C_20_H_35_N_2_O_4_ 367.2597. Found: 367.2589; HPLC analysis: flow rate: 0.5 mL min^−1^, peak area 99.5%, *t*_R_ = 4.4 min, gradient: 30% CH_3_CN for 1 min, 30% to 100% CH_3_CN over 4.5 min, 100% CH_3_CN for 2.5 min, 100% to 30% CH_3_CN over 0.1 min and 30% CH_3_CN for 2 min.

Compound A19: DHA was reacted with 1,2,3,4-tetrahydroisoquinoline according to general procedure. Yield = 40%; pale-yellow oil; *R*_f_ (PhMe/EtOAc 98 : 2) = 0.35; ^1^H NMR (CDCl_3_, 600 MHz) *δ* 7.12–7.05 (m, 4H), 5.34 (s, 1H), 3.30 (br s, 1H), 2.94–2.72 (m, 4H), 2.43–2.33 (m, 2H), 2.08–1.99 (m, 2H), 1.93–1.85 (m, 2H), 1.77–1.68 (m, 3H), 1.60–1.57 (m, 2H), 1.45–1.41 (m, 3H), 1.37 (s, 3H), 0.99–0.93 (m, 6H) ppm; ^13^C NMR (CDCl_3_, 151 MHz) *δ* 135.0, 128.7, 126.7, 103.9, 91.8, 89.7, 80.4, 79.0, 51.8, 51.4, 46.0, 44.4, 37.4, 36.2, 34.3, 34.1, 30.0, 28.8, 26.0, 24.8, 21.7, 20.3, 16.2, 13.6. ppm; HR-ESI (*m*/*z*) [M + H]^+^ calculated for C_24_H_34_NO_4_ 400.2488. Found: 400.2483 and [M + Na]^+^ calculated for C_24_H_33_NNaO_4_ 422.2307. Found 422.2301; HPLC analysis: flow rate: 0.5 mL min^−1^, peak area 98.2%, *t*_R_ = 2.0 min, gradient: 90% CH_3_CN for 1 min, 90% to 100% CH_3_CN over 4 min, 100% CH_3_CN for 3 min, 100% to 90% CH_3_CN over 0.1 min and 90% CH_3_CN for 2 min.

### 2.2 Biological assays

#### 2.2.1. Materials, chemicals and instruments

Rho123, 3-(4,5-dimethyl-2-thiazolyl)-2,5-diphenyl-2*H*-tetrazolium bromide (MTT), histopaque 1077 and lectin from *Phaseolus vulgaris* (PHA) were purchased from Sigma Aldrich (Sigma-Aldrich Co., Buenos Aires, Argentina). Doxorubicin hydrochloride (Dox, 99.8%, Synbias Pharma Ltd) was obtained from Nanox Release Technology (Buenos Aires, Argentina) and was dissolved in bi-distilled water just prior to use. Verapamil hydrochloride 98.0% was provided by Parafarm (Buenos Aires, Argentina) while tariquidar was purchased from ChemFaces Biochemical Co., Ltd (Wuhan, China). Both known P-gp inhibitors^41^ were prepared in DMSO. Flow cytometry was carried out in a Life Technologies Attune-NxT flow cytometer with 96-well autosampler (Thermo Fisher Scientific, USA).

#### 2.2.2. Cell lines and cell culture

The doxorubicin-sensitive chronic myeloid leukemia (CML) cell line K562 and its counterpart P-gp-overexpressing K562/Dox cells were kindly provided by Dr V. Rumjanek, Universidade Federal do Rio de Janeiro, Rio de Janeiro, Brazil.^42^ Both cell lines were cultured in RPMI-1640 medium (Invitrogen Life Technologies, Carlsbad, CA) supplemented with 10% fetal bovine serum, 2 mM l-glutamine, 100 U per mL penicillin, and 100 µg mL^−1^ streptomycin (Invitrogen Life Technologies, Carlsbad, CA) at 37 °C in a 5% CO_2_ humidified incubator. Cells were passaged twice weekly, and only cultures in the exponential growth phase with viability greater than 90%, as determined by trypan blue exclusion, were used for the experiments. To maintain the chemo-resistant phenotype, K562/Dox cells were continuously cultured in the presence of 60 nM of Dox and transferred to drug-free medium 4 days before each experiment. The IC_50_ of Dox was 16.71-fold higher against the resistant K562/Dox cells compared to the sensitive K562 (IC_50_ values of 109.30 ± 9.73 and 6.54 ± 0.88 µM, respectively). This level of resistance correlates with the level of expression of P-gp.^43^

#### 2.2.3. MTT assay

The effect of the target compounds on cell proliferation was determined using the MTT colorimetric assay as previously described.^20^ Briefly, K562 and K562/Dox cells (5 × 10^4^ cells per well) were seeded into 96-well plates containing RPMI-1640 medium and exposed to serial dilutions of each compound starting at a maximum concentration of 50 µM. A negative control containing 0.5% (v/v) DMSO was included in parallel. No significant differences in cell viability were observed between the negative control and untreated cells. Following 48 h of continuous incubation at 37 °C in a humidified atmosphere containing 5% CO_2_, MTT solution was added to each well and the plates were incubated for an additional 4 h. The optical density was measured at 595 nm using an iMark™ microplate reader (Bio-Rad, USA).

#### 2.2.4. Rhodamine 123 and doxorubicin intracellular accumulation assays

Since Rho123 and Dox are both substrates of P-gp, their intracellular fluorescence intensity provides a reliable measure of transporter activity. Accordingly, intracellular dye accumulation was quantified by flow cytometry.^20,37^ Briefly, 5 × 10^4^ cells were seeded in 96-well plates, and incubated with the test compounds for 1 h at 37 °C in a humidified atmosphere containing 5% CO_2_. Rho123 (500 ng mL^−1^) was then added. In a separate set of experiments, Dox at 5 µM was administered. In both assays, the plates were incubated for an additional 1 h under the same conditions. After incubation, cells were washed twice with cold PBS, and 15 000 events per sample were acquired to determine cell-associated fluorescence. Dead cells were excluded based on forward- and side-scatter characteristics. Rho123 and Dox were excited at 488 nm, and the emitted fluorescence was collected using 530/30 nm and 585/42 nm bandpass filters, respectively. Negative controls with 0.5% DMSO were used for comparisons. Intracellular fluorescence was expressed as the mean fluorescence intensity (MFI). The P-gp-modulating effect of the artemisinin derivatives on P-gp transport activity was expressed as the fluorescence activity ratio (FAR) calculated as MFI of Rho123 or Dox with compound/MFI of Rho123 or Dox alone.

#### 2.2.5. Doxorubicin resistance reversal assay

To asses the ability of the most potent compounds to reverse P-gp-mediated MDR and restore Dox sensitivity in K562/Dox cell, a resistant reversal assay was performed.^20,23,37^ MTT assay was carried out as described above. K562 and K562/Dox cells at 5 × 10^4^ cells per well were incubated for 48 h with Dox alone at concentrations ranging from 3.4 to 431 and 0.27 to 34.5 µM, respectively or in combination with the artemisinin derivatives A16 and A20 at concentrations ranging from 0.025 to 0.40 µM and 0.0025 to 0.40 µM, respectively. Wells containing 0.5% v/v DMSO were used as negative controls while verapamil was included as positive control. The fold reversal (FR) values were calculated as the ratio of the IC_50_ of Dox to the IC_50_ of Dox in the presence of the reversal agent.

#### 2.2.6. Cytotoxicity on peripheral blood mononuclear cells and hemolysis assay

The cytotoxicity of compounds A16 and A20, was evaluated on normal human cells. Peripheral blood mononuclear cells (PBMC) were isolated from heparinized blood of healthy donors using Ficoll-Hypaque density-gradient centrifugation. The study protocol was approved by the Research Ethics Board of the Catholic University of Córdoba, and written informed consent was obtained from participants. Cell viability was assessed using the MTT assay, as previously described.^37^ Compounds A16 and A20 were tested at concentrations ranging from 0.39 to 50 µM. To determine whether the observed cytotoxicity involved membrane damage, a hemolysis assay was performed using human erythrocyte to evaluate the lytic properties of the compounds.^37^ Negative controls contained DMSO only.

#### 2.2.7. Data analysis

All data are expressed as the mean ± standard error (SE). The statistical analyses were performed using GraphPad Prism 7.0. For P-gp modulation assays, each treatment concentration was compared with its corresponding untreated control within the same experimental plate using a one-tailed paired Student's *t*-test. This paired analysis was selected because treated and control samples were evaluated within the same independent experiment/plate. We used a one-tailed test based on the predefined directional hypothesis that P-gp modulation would increase intracellular Rho123 or Dox accumulation. For other experiments, one-tailed unpaired Student's *t*-tests were used, as appropriate. Differences in values with *p* ≤ 0.05 were considered significant. Each experiment was performed in triplicate or quadruplicate and done at least three times independently.

### 2.3 Molecular modeling studies

The model for the receptor was built from the cryo-EM (cryo electron microscopy) structure 6QEX co-crystallized with paclitaxel.^44^ Blind docking was performed to validate and optimize the docking protocol using paclitaxel with structure 6QEX, as well as vincristine with the co-crystallized structure 7A69.^45^ When comparing Autodock 4.2.6 and Vina 1.2.6, the latter demonstrated superior performance and was therefore selected for docking the entire compound set.^46^

The noticeable details for Vina were the use of a very large box including the whole transmembrane barrel which implies the absence of any assumption about the preferred binding site. Taking into account the size of the box, the “exhaustiveness” parameter was increased from the default of 8 and the procedure repeated at least 16 times by varying in each simulation the “exhaustiveness” from 32 to 256.

Gasteiger charges were assigned to the ligands, which were fully geometry-optimized using the semi-empirical PM6 Hamiltonian as implemented in Gaussian 16.^47^

The nature of the minima was confirmed in each case by diagonalization of the Hessian matrix and verification of all-positive eigenvalues. After each Vina run, 12 conformers were saved whenever their binding energy were less than 3 kcal mol^−1^ above the most stable.

The most stable conformers of each ligand was selected as the initial structures for the molecular dynamics (MD) simulations, the protocol of which has been described previously in detail.^20,37^ Briefly, it comprises:

• (I) Force field parametrization and setup: the atomic charges for all ligands were derived using the AMBER20 sqm module, employing semi-empirical AM1 quantum calculations.^48^ Parameters were assigned based on the Generalized Amber Force Field (GAFF2) using the antechamber utility.^49^ The protein was modeled with the ff18SB force field.

• (II) Minimization: 250 steepest descent steps with heavy restraints on the protein backbone, solvated in a TIP3P water box (10 Å padding) and neutralized with chloride ions.

• (III) Minimization: 6500 conjugate gradient steps to fully relax the system.

• (IV) Heating: 100 ps in the NTV ensemble up to 300 K with backbone restraints.

• (V) Stabilization/production during 100 ns in the NTP ensemble (1 atm, 300 K) using the Andersen thermostat/barostat. A 50.0 kcal mol^−1^ Å^−2^ harmonic restraint was applied to the backbone to maintain the transmembrane topology of P-gp. The SHAKE algorithm was applied for contraining hydrogen-heavy atom bonds. Electrostatics was computed with Particle Mesh Ewald (PME) method (10 Å cutoff).

All trajectories were computed using the pmemd.cuda module of AMBER20. The RMSD of each ligand and surrounding residues (5 Å around) stabilized at 1.4–2.0 Å (see Fig. S58). The hydrogen bond analyses were achieved using the last 95 ns of simulation and it is not further discussed, since they showed no clear distinction between potent or poor binders in terms of either experimental MECs or 
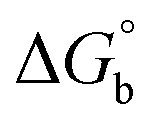
 of each species (examples as Fig. S59).

The binding free energies 
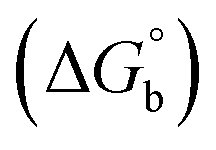
 were calculated using the MM-PBSA/GBSA using the mmpbsa module in AMBER20 (ref. 48 and 50) averaging over the last 80–120 ps of each trajectory and repeating with at least two trajectories. Frames were sampled every 10–20 ps to minimize energy self-correlation. The mmpbsa module MMPBSA.py was used to perform the per residue energy decomposition analysis (Fig. 6) and statistical error averaged from the trajectories analyzed for the uncertainties (Table 4).

• The most populated conformational clusters (last 95 ns) were identified based on RMSD on heavy atoms using Chimera 1.15 and extracted as starting structures for QM/MM.^51^ These representative structures were processed for obtaining the QTAIM analyses of the protein-ligand interactions.^52^ The setup for the ONIOM^53^ calculations involved a very large quantum region including the ligand and all the residues within 5 Å (37–45 amino acids) treated with DFT at the level of theory M06-2X/6-31G(d).^54^ The rest of the protein was the lower layer treated with UFF force field. These models were relaxed (20–40 conjugate gradient steps) using the Gaussian 16 implementation of ONIOM and the final wave function was used for the QTAIM analysis carried out with MultiWFN 3.8.^55^ Non-covalent interactions were characterized by their bond paths and the critical points (BCPs) were computed to quantify electron density (*ρ*(*r*)) at ligand–residue interfaces. The density (Σ*ρ*(*r*)) was summed over BCPs for each ligand moiety, the artemisinin core, the linker and the variable group. It was also summed over each contributing amino acid in contact, *i.e.* per residue contributions (Fig. 10 and 11).

Additional calculations for characterizing the electron-donor and polarizability properties of some of the fragments present as substituents in several compounds were computed at the level of theory CAM-B3LYP/6-311G(d,p) (further details as orbital and total energies in atomic units as Table S2). For the ionization potentials calculations, the total electronic energies of the neutral and radical cations were used, as prescribed in ref. 56, expected to be more accurate than the usual Koopmans' theorem approximation using just the frontier orbitals, in these cases the bases sets were 6-311+G(d,p). The nucleophilicity index was calculated as defined by Chattaraj *et al.*^57^ The LOLIPOP index as introduced in ref. 58, which measures the actual ability of an aromatic ring for forming π-stackings (the lower the index, the more likely to interact with other aromatics), was computed with MultiWfn 3.8. The index for naphthalene as reference at the same level of theory of the subject fragments was found 5.79, close to 5.81 value of reference.^59,60^

Rendering of the structures showing bond paths and BCPs were prepared with MultiWFN 3.8 scripts and VMD 1.9.3.^61^

## 3 Results and discussion

### 3.1 Design and synthetic chemistry

Previous studies revealed that the presence of electron-rich rings with three *O*-methyl groups, the length of the linker connecting the two pharmacophoric moieties, and a flexible NHCH_2_– group, positively contribute to anti-P-gp activity.^20,37^ Building on these findings, a library of artemisinin derivatives was rationally designed and synthesized. The hydrophobic artemisinin scaffold was linked to either a piperazine ring or electron-rich aromatic moieties through flexible linkers of varying lengths. The aromatic rings were functionalized with electron-donating substituents, ranging from a hydroxyl group to three *O*-methyl groups. Haloaminoquinoline rings, predicted to be more electron-rich and polarizable than the 3,4,5-trimethoxyphenyl group, were also incorporated as electron-rich π systems. These features were examined using quantum chemical calculations comparing anilines, substituted toluenes, and substituted quinolines. The results for some of these rings are summarized in Table 1, highlighting the most desirable electronic features for the fragments (further details are provided in the SI, section Molecular modeling details, Table S2). Based on the properties in Table 1, the inclusion of haloaminoquinoline rings would be desirable to fulfill the profile proposed and to widen the chemical diversity within the series. Most linkers contained at least two flexible methylene units. The synthetic routes to the target artemisinin derivatives are outlined in Schemes 1 and 2. Scheme 1 describes the synthesis of compounds A1–A12, whereas Scheme 2 illustrates the preparation of compounds A13–A20.

**Table 1:** Calculated electronic properties for fragments commonly found in the artemisinin derivative series. Most likely values in bold^a^

Properties^a^	IP^b^/eV	Dipole/Debye	Nucleophilicity index^c^	Polarizability/a.u.	LOLIPOP index
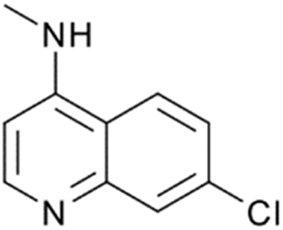	**7.608**	5.45	**3.90**	**140.3**	1.67, 3.18
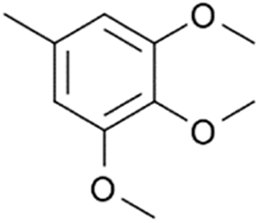	7.894	2.56	3.59	121.5	2.76
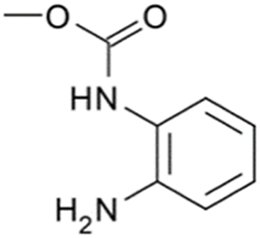	7.886	5.63	3.58	116.6	2.43
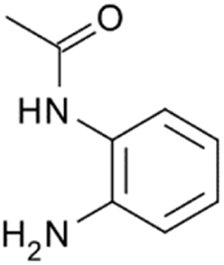	8.004	4.35	3.57	102.6	**2.33**
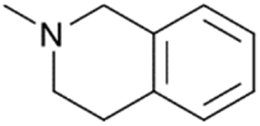	8.134	0.77	3.37	111.0	3.50

a Further details as Table S2.

b Calculated from the total energies of the neutral and radical cation.

c Reference HOMO energy of tetracyanoethylene at the same level of theory: −10.990 eV.

Scheme 1, outlines the synthesis of artesunate (AS) from artemisinin, followed by its coupling with a series of hydroxylamines, anilines, and amines to afford the designed derivatives A1–A12. In contrast, derivatives A13–A20 were synthesized through a different strategy, as illustrated in Scheme 2. Starting from DHA, the corresponding ether compound A21 was first prepared and subsequently reacted with 4-nitrophenyl chloroformate to generate the key activated carbonate intermediate A22. This intermediate was then coupled with appropriately selected amines to afford derivatives A13–A16 and A20. In a parallel approach, DHA was converted into the corresponding 10-aza analogues A17–A19 by treatment with the appropriate amines under DCM/oxalyl chloride/DMSO/xylene/DIPEA conditions, as described below.

### 3.2 Biological evaluation

#### 3.2.1. Artemisinin-derived analogues selectively inhibit P-gp without affecting cell viability

Before determining whether the artemisinin-derived analogues (Fig. 1) could reverse the MDR phenotype through inhibition of P-gp, their effects on cell viability were assessed using the MTT assay. The results showed that neither the synthesized compounds nor artemisinin, artesunate, and dihydroartemisinin (DHA) exerted cytotoxic activity on K562/Dox or K562 cells (Table 2). All compounds displayed half-maximal inhibitory concentration (IC_50_) values above the 10 µM threshold established by the U.S. National Cancer Institute (NCI) to consider a compound as cytotoxic.^62^ In contrast, according to this criterion, the potent P-gp blocker tariquidar exhibited cytotoxic activity, with IC_50_ values of 2.4 and 2.2 µM toward K562/Dox and K562 cells, respectively (Table 2).

**Table 2:** Antiproliferative effect of the derivatives on K562 and its multidrug-resistant counterpart, K562/Dox cells^a^

Compounds	IC_50_ (µM)	
	K562/Dox	K562
Artemisinin	>50	>50
Artesunate	>50	>50
DHA	>50	>50
A2	>50	>50
A4	>50	>50
A5	>50	>50
A10	>50	>50
A19	>50	>50
A3	>50	>50
A11	>50	>50
A18	>50	>50
A1	>50	45.39 ± 5.40
A6	>50	>50
A8	>50	>50
A17	>50	>50
A7	36.42 ± 1.35	47.14 ± 2.33
A12	>50	>50
A13	>50	>50
A15	>50	>50
A14	>50	>50
A16	27.55 ± 7.33	18.92 ± 1.95
A20	46.75 ± 9.36	20.73 ± 4.75
Verapamil	>50	>50
Tariquidar	2.38 ± 0.32	2.25 ± 0.28

a DHA: dihydroartemisinin. The maximum concentration tested corresponded to 50 µM. Values are expressed as mean ± SE of three independent experiments (*n* = 3).

As a primary screening, the rhodamine 123 (Rho123) intracellular accumulation assay was performed. As shown in Table 3 compounds A7, A12, A13, A14, A15, A16 and A20 inhibited Rho123 efflux, as indicated by statistically significant fluorescence activity ratio (FAR) values (*p* < 0.0001–0.05) at minimum effective concentrations (MECs) below 10 µM. Notably, these compounds exhibited markedly higher P-gp-modulating activity than artemisinin, artesunate, and dihydroartemisinin, none of which showed detectable anti-P-gp activity at 50 µM (Table S1). The lack of activity toward P-gp of the latter compounds is consistent with previous reports by Reungpatthanaphong and Mankhetkorn,^31^ Efferth *et al.*^63^ and Oga *et al.*^33^ who described little or no effect on P-gp activity for these sesquiterpene lactones. Overall, the enhanced activity of the derivatives highlights the effectiveness of the structural modifications introduced in this series.

**Table 3:** MDR-reversing activity of the most active artemisinin derivatives evaluated^a^

Compounds	FAR														
	K562/Dox												K562		
	Concentration (µM)												Concentration (µM)		
	50	25	12.5	6.25	3.12	1.56	0.78	0.39	0.19	0.098	0.049	0.024	50	25	12.5
A7	3.20 ± 0.24***	1.98 ± 0.07***	1.33 ± 0.06**	1.16 ± 0.04*	1.03 ± 0.09									0.78 ± 0.04*	
A12	4.01 ± 0.67*	3.66 ± 0.63*	2.60 ± 0.47*	1.63 ± 0.10**	1.51 ± 0.14*	1.08 ± 0.11							0.90 ± 0***		
A13	3.28 ± 0.35***	2.91 ± 0.32***	2.43 ± 0.24***	1.89 ± 0.14**	1.50 ± 0.08**	1.40 ± 0.10*	1.19 ± 0.03**	1.04 ± 0.09					0.99 ± 0.02		
A14	3.81 ± 0.58**	3.24 ± 0.41**	2.76 ± 0.32**	2.28 ± 0.19***	1.88 ± 0.23**	1.66 ± 0.18**	1.35 ± 0.05*	1.23 ± 0.04**	1.16 ± 0.03*	0.99 ± 0.06			0.96 ± 0.05		
A15	3.41 ± 0.38***	3.51 ± 0.41***	2.85 ± 0.27***	2.31 ± 0.26***	1.76 ± 0.15***	1.39 ± 0.07**	1.17 ± 0.05*	1.20 ± 0.04*	**1.14** ±0.01**	1.00 ± 0.08			0.84 ± 0.02**		
A16		3.41 ± 0.37****	3.39 ± 0.44****	2.89 ± 0.34****	2.45 ± 0.24****	1.95 ± 0.15****	1.61 ± 0.08****	1.49 ± 0.07***	1.40 ± 0.12**	1.21 ± 0.04***	1.09 ± 0.0s4*	0.96 ± 0.03			0.89 ± 0.01**
A20		5.40 ± 0.13****	4.64 ± 0.19****	3.84 ± 0.08****	2.89 ± 0.10****	2.20 ± 0.16****	1.67 ± 0.12****	1.46 ± 0.09***	1.22 ± 0.05***	1.20 ± 0.06*	1.23 ± 0.04**	0.99 ± 0.03			0.91 ± 0.03*
Verapamil	5.65 ± 0.43****	5.13 ± 0.48****	4.55 ± 0.37****	3.79 ± 0.36****	3.02 ± 0.24****	2.16 ± 0.34**	1.61 ± 0.15**	1.23 ± 0.07*	1.08 ± 0.04				0.98 ± 0.05		
Tariquidar	1.89 ± 0.30****	1.50 ± 0.15***	1.42 ± 0.11***	1.34 ± 0.05****	1.14 ± 0.03**	1.20 ± 0.03***	1.06 ± 0.03						1.02 ± 0.05		

a Fluorescence activity ratio (FAR) = mean fluorescence intensity (MFI) of rhodamine 123 (Rho123) with compound/MFI of Rho123 alone. The minimum effective concentrations (MECs) of each compound are shown in bold. Values are expressed as mean ± SE from at least four independent experiments (*n* ≥ 4). Significant differences between treated and untreated cells were determined by using the one-tailed paired Student's *t*-test (****p < 0.0001, ***p < 0.001, **p < 0.01, *p < 0.05).

#### 3.2.2. Structural optimization identifies compounds A16 and A20 as potent P-gp modulators

In particular, compounds A16 and A20 exhibited remarkable activity, as evidenced by MEC values of 0.049 µM (Table 3 and Fig. 2A), which were lower than those of the reference compounds verapamil and tariquidar (MEC = 0.39 and 1.56 µM, respectively). The FAR values obtained in K562/Dox cells treated with compound A16 at concentrations ranging from 25 to 1.56 µM were lower than those observed with verapamil at the corresponding concentrations, whereas compound A20 produced FAR values comparable to those of verapamil over the same concentration range (Table 3). Although higher FAR values and lower MECs were expected for tariquidar, the results obtained are consistent with previous reports using different cell lines and pre-incubation times in Rho123 accumulation assays.^64,65^ Notably, Li *et al.*,^66^ reported that tariquidar inhibited Rho123 efflux from K562/P-gp cells at 0.5 µM, exhibiting a level of activity comparable to that observed in the present study. As shown in Fig. 2A, treatment with compound A16 at 0.39 µM significantly increased Rho123-associated mean fluorescence intensity (MFI), indicating inhibition of P-gp-mediated efflux with higher Rho123 accumulation than that observed with verapamil at the same concentration (MFI: 2366 ± 164 and 1255 ± 35, respectively; *p* < 0.001). Similarly, compound A20 significantly increased intracellular Rho123 accumulation at both 25 and 0.39 µM with significantly higher MFI values compared with verapamil (Fig. 2A).

**Fig. 2:**
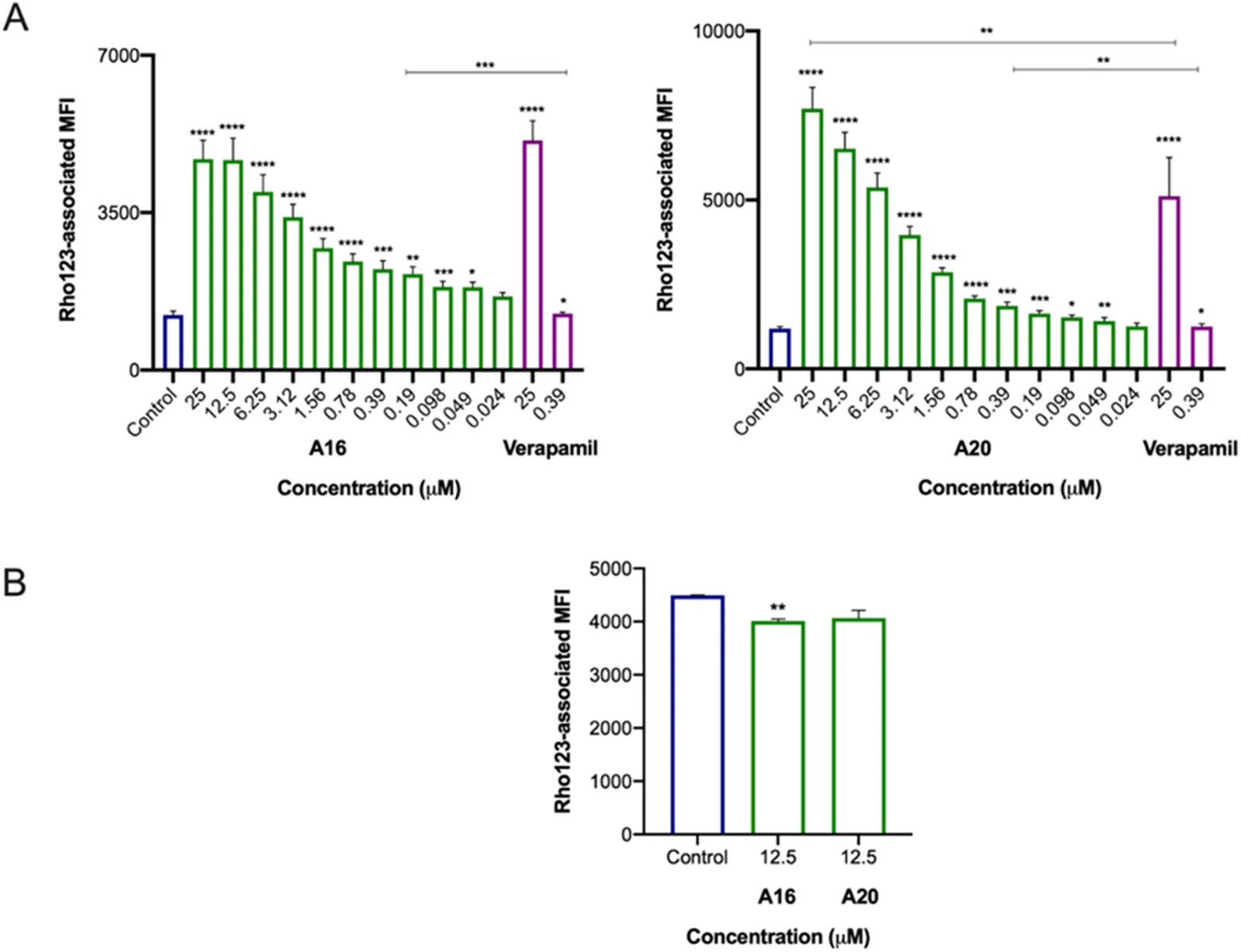
Inhibition of P-gp-mediated rhodamine 123 (Rho123) efflux by different concentrations of compounds A16 and A20 (A), as determined by the accumulation assay in the K562/Dox cell line. Intracellular Rho123-associated fluorescence significantly increased in K562/Dox cells following treatment with the target compounds, whereas no significant changes were observed in K562 cells treated with compounds A16 and A20 at 12.5 µM (B). Values are expressed as mean ± SE from at least four independent experiments (*n* ≥ 4). Statistical significance relative to the respective negative control was assessed using a paired one-tailed Student's *t*-test (****p < 0.0001, ***p < 0.001, **p < 0.01, *p < 0.05).

The MFI values of Rho123 in untreated K562 cells were comparable to or even higher than, those in cells treated with compounds A16 or A20 (Fig. 2B). These findings indicate that the increased Rho123 accumulation in the chemoresistant cells resulted from inhibition of P-gp-mediated efflux by the tested compounds.

As previously reported, the presence of three methoxy (–OCH_3_) groups in a series of quinolinone–pyrimidine hybrids and betulin derivatives significantly enhanced anti-P-gp activity^20,37^ a trend that was also observed in the present study. This is supported by comparison of compound A6 (MEC = 12.5 µM) with the structurally related but inactive analogues A3, A4 and A5 which bear alternative substituents (Table S1). Compounds A6, A7, A8, A14 and A15, all containing three *O*-methyl substituents and an alkyl-carbamate linker effectively inhibited Rho123 efflux with MEC values ranging from 0.19 to 12.5 µM (Tables 3 and S1). Among these, compounds A14 and A15, which feature a longer linker connecting the artemisinin core to the methoxybenzene ring and lack esterification, were the most active, exhibiting MEC values of 0.19 µM compared with compounds A6, A7 and A8, which showed MEC values of 6 and 12.5 µM (Tables 3 and S1). Introducing of an additional methylene group in the carbamate chain of compound A7, increased the blocking effect on dye efflux compared with compound A6, however, no further improvement in activity was observed upon the introduction of a second methylene unit, as in compound A8. Comparing the activity of compound A20 with that of compound A12, which exhibited MEC values of 0.049 and 3.12 µM, respectively, indicates that esterification and the shorter linker in compound A12 reduced probe retention. Overall, these results suggest that incorporating a haloaminoquinoline group contributes to enhanced P-gp modulation, resulting in the lowest effective concentrations among the tested derivatives.

#### 3.2.3. Compounds A16 and A20 restore intracellular Dox accumulation and reverse multidrug resistance

To further evaluate the MDR reversal effect by targeting P-gp of compounds A16 and A20 on P-gp activity, a Dox accumulation assay was conducted. Treatment with Dox in combination with compound A16 resulted in a significant increase in intracellular accumulation, with FAR values ranging from 1.29 ± 0.09 to 1.65 ± 0.09 at concentrations from 0.19 to 25 µM (Fig. 3A). Similarly, co-administration of Dox with compound A20 produced a significant increase in intracellular accumulation, with FAR values ranging from 1.19 ± 0.02 (*p* < 0.05) to 1.52 ± 0.12 (*p* < 0.01) at concentrations from 0.049 to 25 µM (Fig. 3B). Comparable MFI values were observed in parental K562 cells treated with either A16 or A20 relative to untreated controls (Fig. 3C), indicating that the effects were specifically associated with modulation of P-gp-mediated drug resistance rather than nonspecific changes in Dox uptake.

**Fig. 3:**
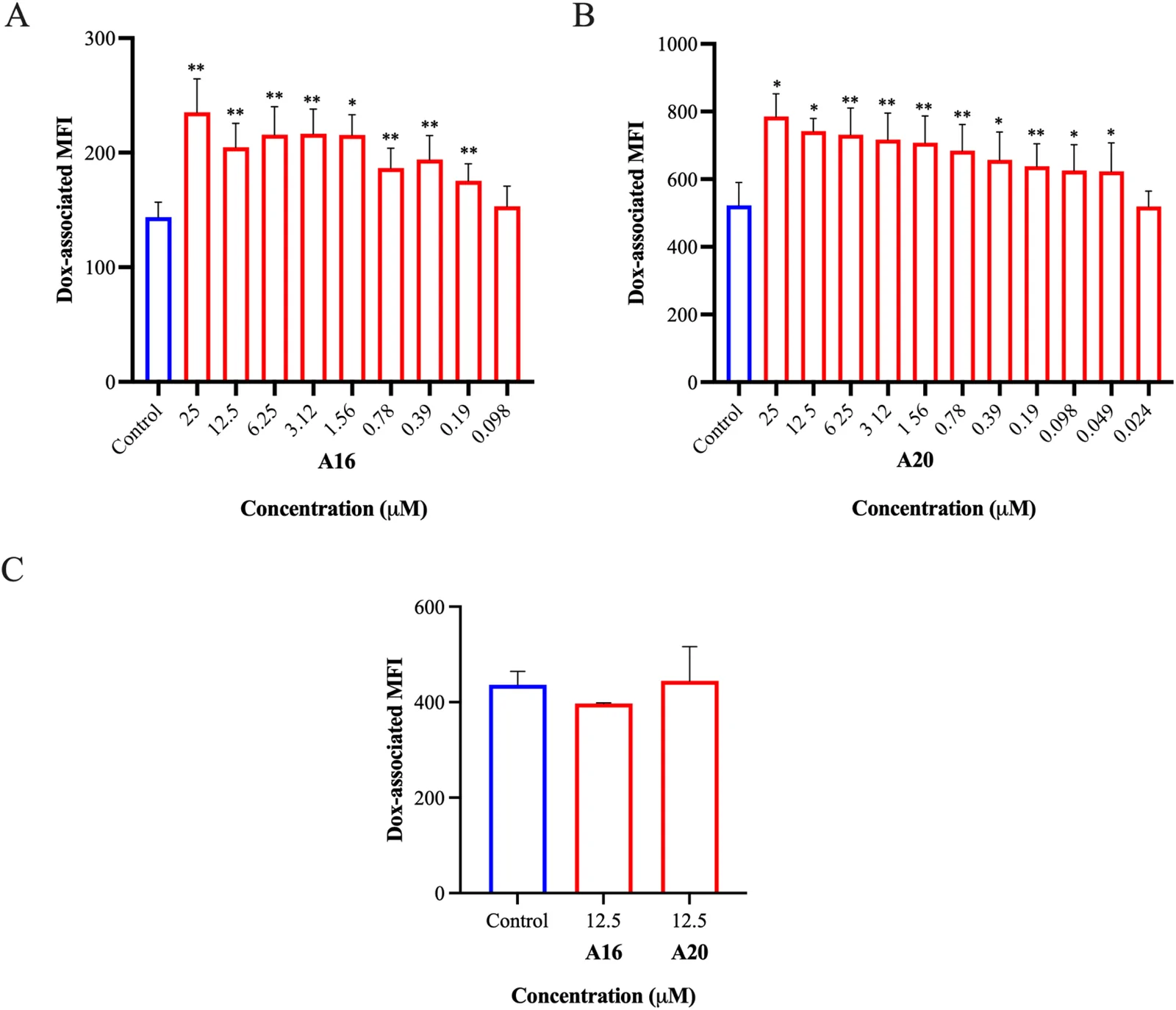
Inhibition of P-gp-mediated doxorubicin (Dox) efflux by different concentrations of compounds A16 (A) and A20 (B), as determined by the accumulation assay in the K562/Dox cell line. Intracellular Dox-associated fluorescence significantly increased in K562/Dox cells following treatment with the target compounds, whereas no significant changes were observed in K562 cells treated with compounds A16 and A20 at 12.5 µM (C). Values are expressed as mean ± SE from at least four independent experiments (*n* ≥ 4). Statistical significance relative to the respective negative control was assessed using a paired one-tailed Student's *t*-test (****p < 0.0001, ***p < 0.001, **p < 0.01, *p < 0.05).

Building on the observed increase in intracellular Dox accumulation, we next evaluated whether this effect translated into reversal of drug resistance by assessing Dox cytotoxicity in K562/Dox cells after co-treatment with the target compounds. As shown in Fig. 4, both compound A16 and compound A20 significantly increased the sensitivity of K562/Dox cells to Dox, as evidenced by reduced IC_50_ values. Compound A16 achieved a maximal fold-reversal (FR) value of 4.17 ± 0.95 (*p* < 0.01) at 0.40 µM, whereas compound A20 displayed a pronounced concentration-dependent effect (*b* = 101.4; 95% CI 48.89–153.9; *p* < 0.01; *R*^2^ = 0.79), reaching a maximal FR value of 46.36 ± 10.78 (*p* < 0.01) at the same concentration. The results indicated that both artemisinin derivatives could increase Dox potency in K562/Dox cells at very low concentrations with compound A16 showing an FR of 1.53 ± 0.05 (*p* < 0.001) at 0.05 µM and compound A20 showing an FR of 1.12 ± 0.04 (*p* < 0.05) at 0.005 µM.

**Fig. 4:**
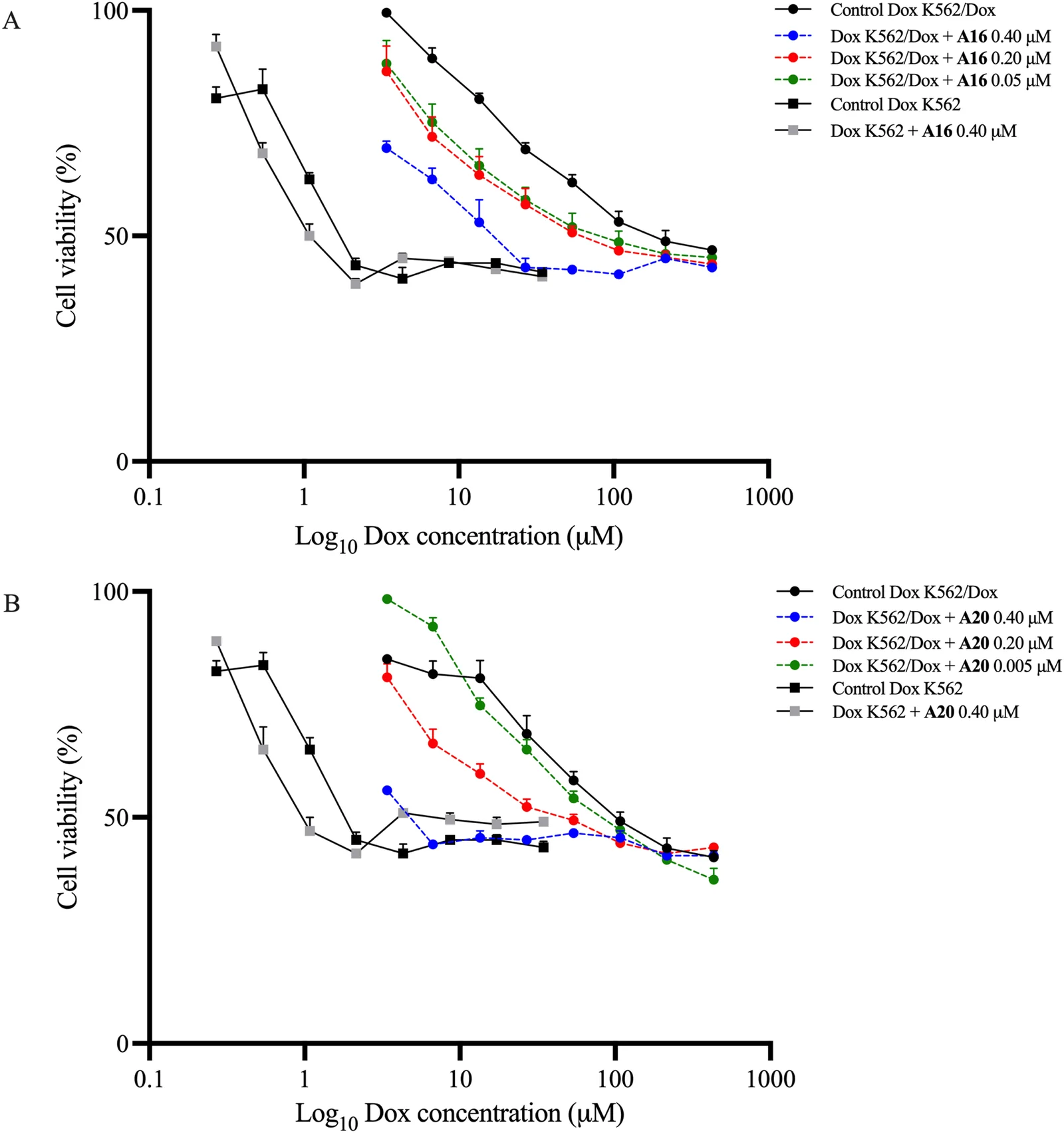
Dose–response curves showing that artemisinin derivatives A16 (A) and A20 (B) reverse doxorubicin (Dox) resistance in K562/Dox cells. A sensitizing effect was observed with compound A16 (0.05–0.40 µM) and compound A20 (0.005–0.40 µM). Assays in parental K562 cells ruled out changes in Dox cytotoxicity unrelated to P-gp modulation. Values are expressed as mean ± SE from three independent experiments (*n* = 3).

### 3.3 Cytotoxicity on human normal cells

Given the outstanding activity, compounds A16 and A20 were selected for further evaluation of their biological profile to explore potential pharmacological applications. Cytotoxicity was assessed in normal human peripheral blood mononuclear cells (PBMCs) using the MTT assay. The compounds exhibited IC_50_ values of 8.47 ± 0.06 µM for A16 and 6.06 ± 0.39 µM for A20. When compared with the lowest concentrations that produce significant reversal in K562/Dox cells (0.05 µM for A16 and 0.005 µM for A20), these results indicate an *in vitro* safety margin of approximately 170-fold for compound A16 and 1200-fold for compound A20, supporting their potential for P-gp modulation at concentrations below those associated with cytotoxicity in PBMCs. In addition, neither compound A16 nor A20 showed detectable hemolytic activity at 50 µM, the highest concentration tested in the hemolysis assay, further supporting their favorable cellular tolerability profile under the experimental conditions.

Although compounds A16 and A20 showed favorable *in vitro* safety margins, these results provide only an initial indication of cellular tolerability and cannot fully predict *in vivo* safety. Further *in vivo* pharmacokinetic and toxicity studies are therefore needed to determine whether these favorable margins translate into an acceptable therapeutic window.

### 3.4 Molecular modeling studies

#### 3.4.1. Molecular dynamics reveals a dual-contact binding mode associated with potent P-gp inhibition

To elucidate the binding mode of the most potent compounds A16 and A20 computational analyses were performed. The entire series of compounds was docked into the P-gp structure without imposing prior assumptions about the binding region, thereby allowing exploration of nearly the entire transmembrane domain. This protocol, described previously in detail,^20,67^ was validated through blind docking using the co-crystallized structures of taxol and vincristine as reference chemotherapeutic agents. All compounds shown in Fig. 1 localized within or near the apex of the inverted “V” shape formed by TMHs 4, 5 and 6 from the first half of the protein and TMHs 7 and 12 from the second half.

In our previous studies,^20,37,67^ we observed that only tight binding to this specific site, despite the calculated stability from long molecular dynamics (MD) energetic analyses, correlated with modulating activity. To further investigate this relationship, twelve representative docked structures were selected as starting structures for 100–110 ns MD simulations. These compounds were chosen to encompass the full spectrum of structural diversity and biological activity, including four inactive or weakly active compounds, four moderately active compounds, and the four compounds showing the strongest P-gp-modulating activity, as defined by their MEC values determined from the Rho123 accumulation assay (MECs highlighted in bold in Tables 3 and S1). The calculated free energies of binding 
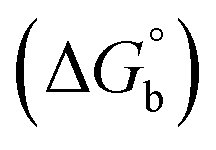
 from MM-PBSA analyses of the MD trajectories are summarized in Table 4. A reasonable good correlation (*R*^2^ = 0.834) between MECs and 
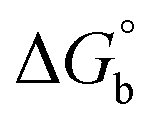
 is shown as Fig. S65.

**Table 4:** Summary of the calculated free energies of binding from the MM-PBSA analyses of the MD trajectories

Species	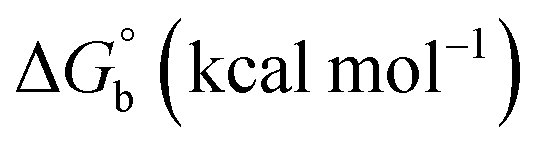	MEC (µM)
**Substrates**		
Rhodamine 123	−25.3^a^	
Doxorubicin	−24.2^a^	

**Reference**		
Verapamil	−35.7	0.39

**Subject compounds**		
A19	−17.64	>50
DHA	−18.57	>50
A17	−25.79	12.5
A2	−26.32	>50
A3	−26.64	50
A18	−28.2	50
A12	−30.75	3.12
A13	−30.81	0.78
A8	−32.15	12.5
A14	−37.87	0.19
A16	−39.17	0.049
A20	−43.21	0.049

a Taken from reference Laiolo *et al.*, 2021.^67^ DHA: dihydroartemisinin. MEC: minimum effective concentration by rhodamine 123 (Rho123) accumulation assay.

The most potent modulators, particularly compound A20, adopted a characteristic “U” shaped conformation, that enabled simultaneous tight contacts with TMH6 and TMH12. A representative snapshot corresponding to the most populated conformation from the cluster analysis of the trajectory is shown in Fig. 5. The main residues involved in the binding of compound A20 (Fig. 5) and compound A16 (SI, Fig. S60 and S61) were identified as critical for inhibition, consistent with previous findings involving libraries of compounds with different chemical scaffolds featuring a hydrophobic moiety, a linker and a variable group.^20,37^ These residues mainly include in TMH4: Ser228, Ala229, Trp232, Leu236; in TMH5: Phe303, Tyr307, Ile360; in TMH6: Phe336, Leu339, Ile340, Phe343, Gln347; in TMH7: Gln725, Phe728 and in TMH12: Val982, Phe983, Met986, Ala987, Gln990. Most of these interactions remained stable throughout the simulations for compounds exhibiting highly favorable 
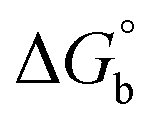
 values (below −30 kcal mol^−1^) and MEC values below 10 µM (Table 4). Notably, these residues overlap with those involved in the binding of the chemotherapeutic agents paclitaxel, vincristine and Dox, as supported by crystallographic and computational studies^20,37,45^ (see also SI, Fig. S61). To gain further insight into the energetic determinants of ligand binding, the MM-PBSA free energy was decomposed on a per-residue basis (Fig. 6). Compounds A20 and A16 exhibited strong interactions with TMHs 4, 6, and 12, a binding pattern also observed for the active compounds A12, A13, and A14 (SI, Fig. S62). In contrast, weakly active or inactive ligands, such as compound A2 (Table S1 and Fig. 6), lacked stable contacts with TMHs 4 and 6 and showed reduced interactions with TMH12. The twisted “U” conformation adopted by compound A20 positioned its artemisinin moiety deep within the hydrophobic core of the binding cavity, formed mainly by TMHs 4–6 and Met986 from TMH12 (Fig. 7). The relevance of this region had previously been established for unrelated families of potential P-gp inhibitors.^20,37^ In contrast, poorly active or inactive compounds A2 and A3 failed to adopt this favorable binding pose, as illustrated in the comparative analysis of their most populated clusters (Fig. 8). Accordingly, compounds A12 and A20 with 
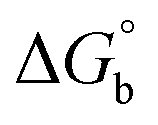
 values of −30.7 and −43.2 kcal mol^−1^, respectively (Table 4) maintained stable simultaneous contacts with both halves of the binding cavity (TMHs 4–6 and TMHs 7–12). Conversely, compounds A2 and A3 with 
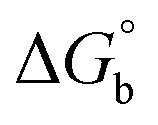
 values of −26.3 and −26.6 kcal mol^−1^, respectively (Table 4) established weaker interactions, particularly with TMH12, and therefore failed to satisfy the dual-contact binding mode consistently observed for potent inhibitors.

**Fig. 5:**
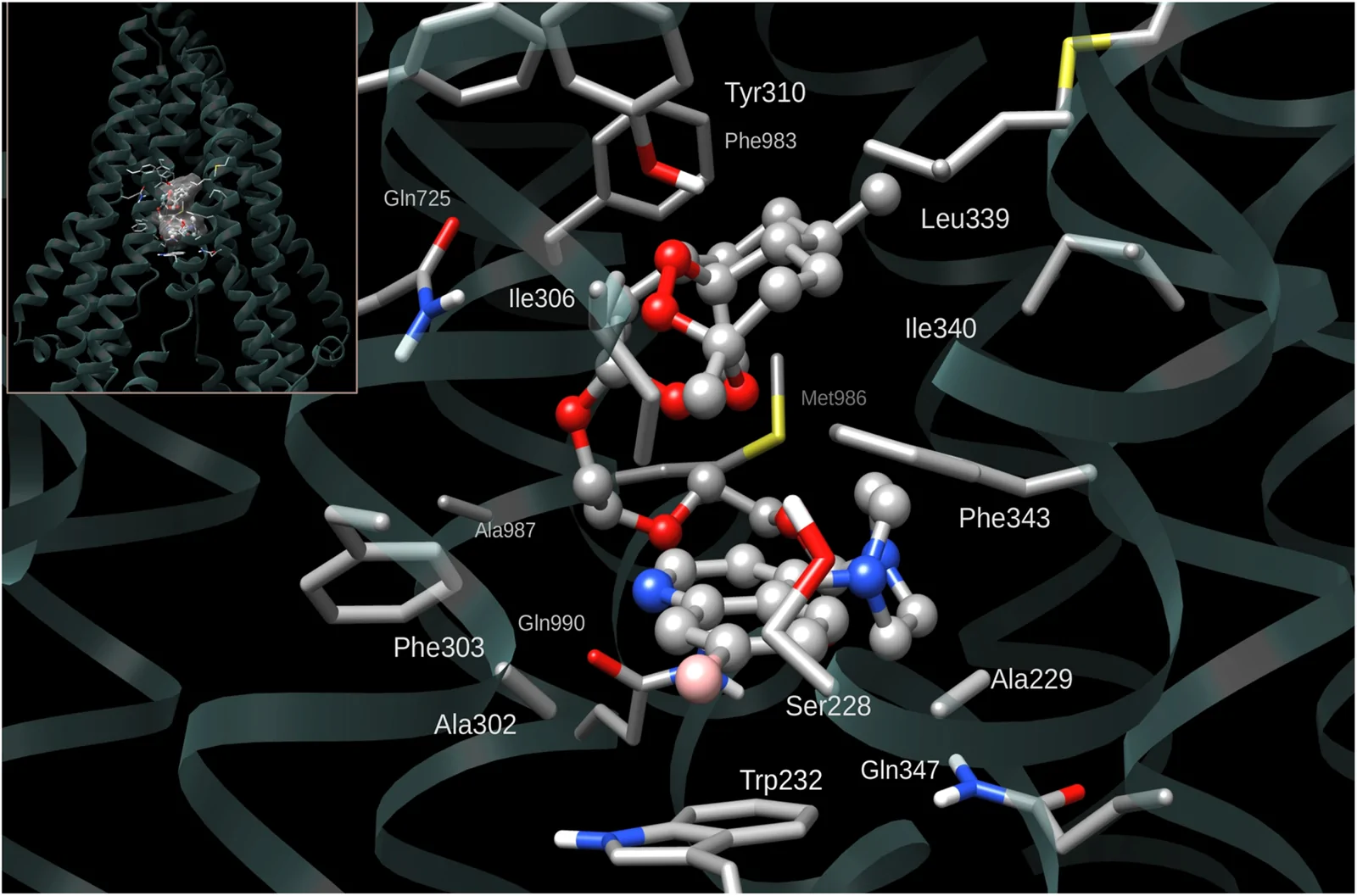
Binding pose of compound A20 featuring the most populated cluster from the analysis of the last 90 ns of trajectory.

**Fig. 6:**
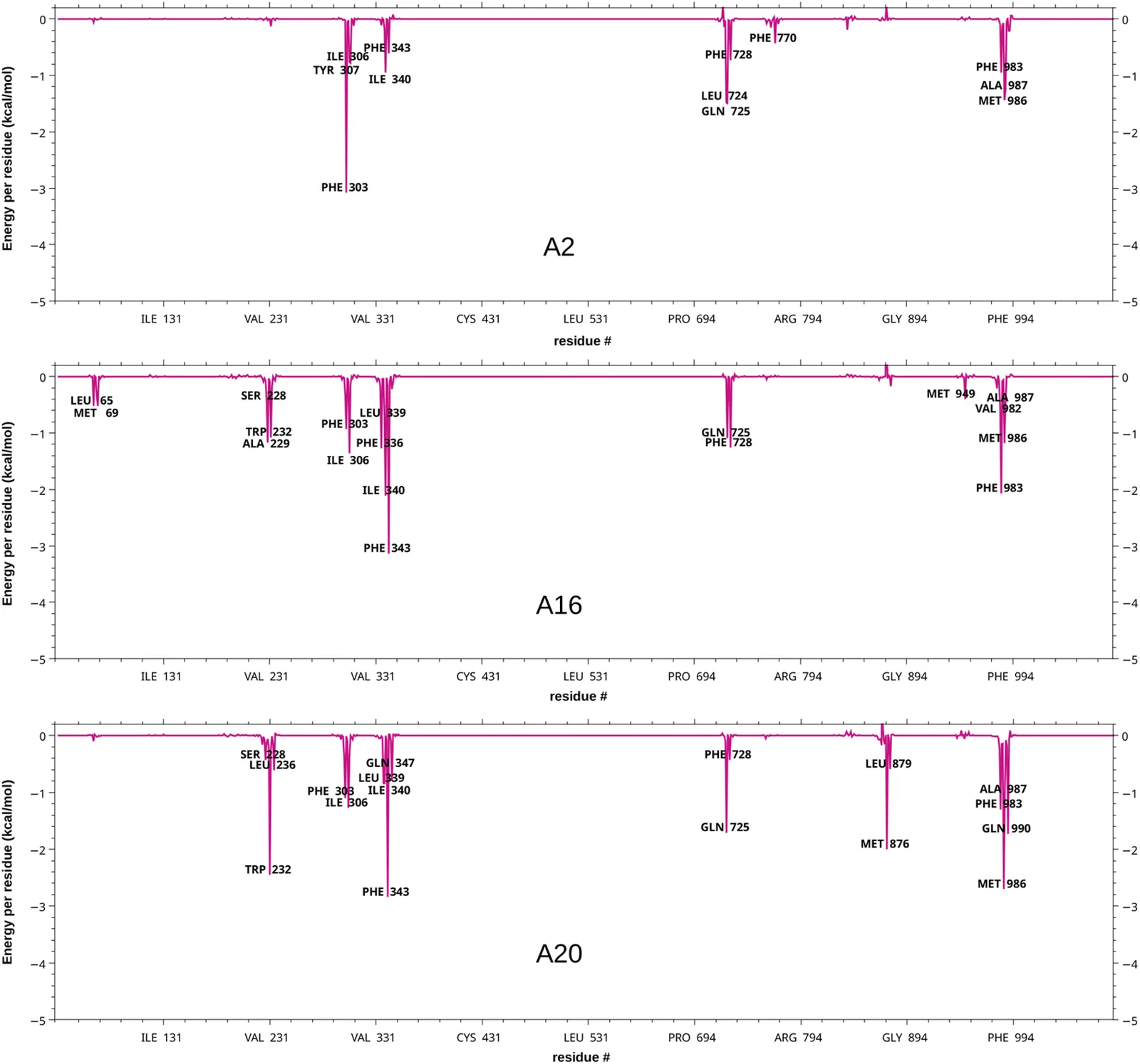
Per residue decomposition of the contribution to the total 
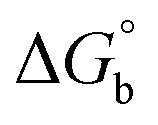
. Analyses over the last part of the trajectories for representative compounds A2, A16 and A20.

**Fig. 7:**
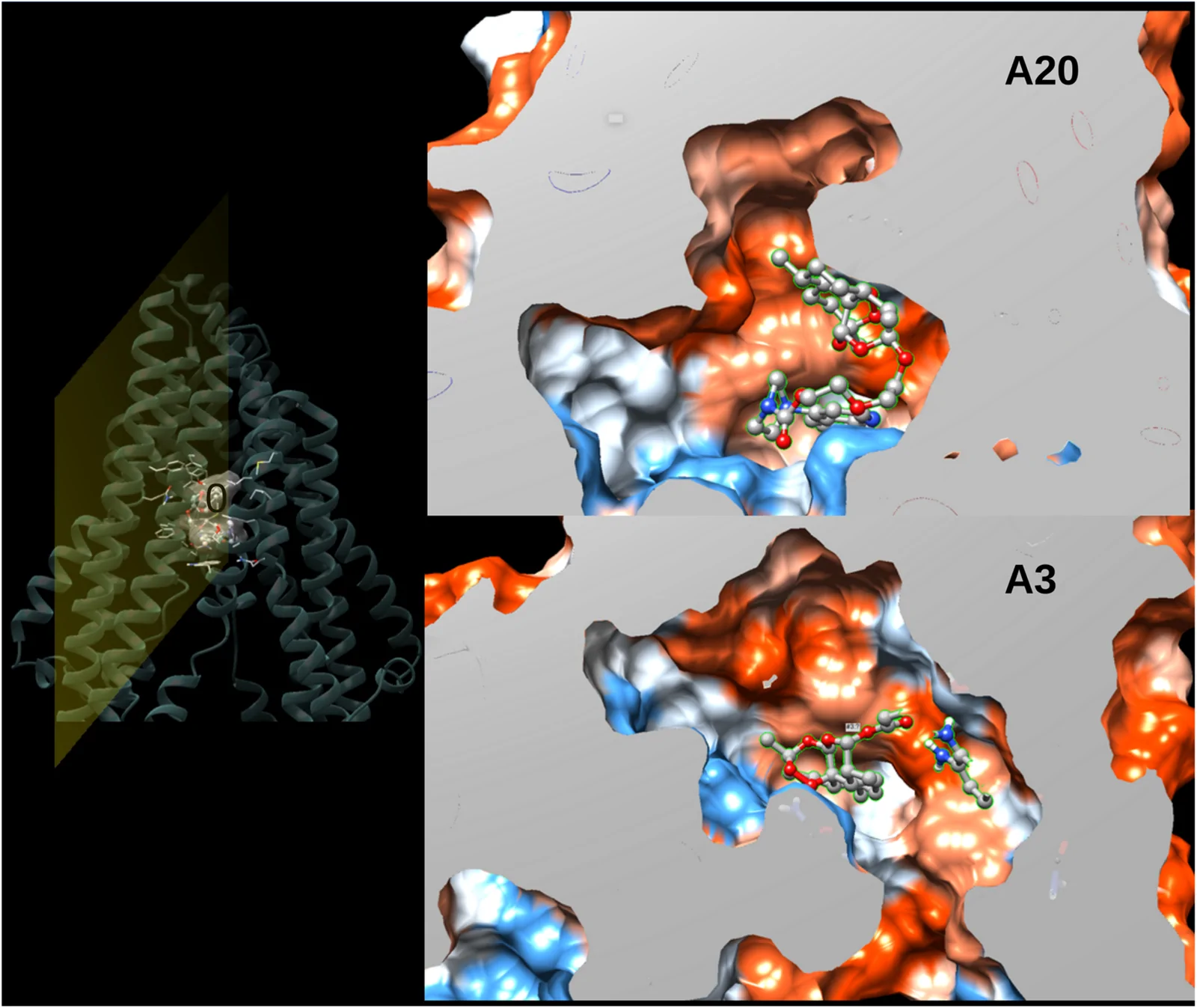
A cross section of the binding pocket. The protein surface is colored from blue (least hydrophobic) to orange (most hydrophobic). Note the hydrophobic region between trans membrane helices (TMHs) 4–6 is occupied by the artemisinin moiety of compound A20 whereas it is not contacted by the artemisinin moiety compound A3 (intense orange empty cavity above the ligand).

**Fig. 8:**
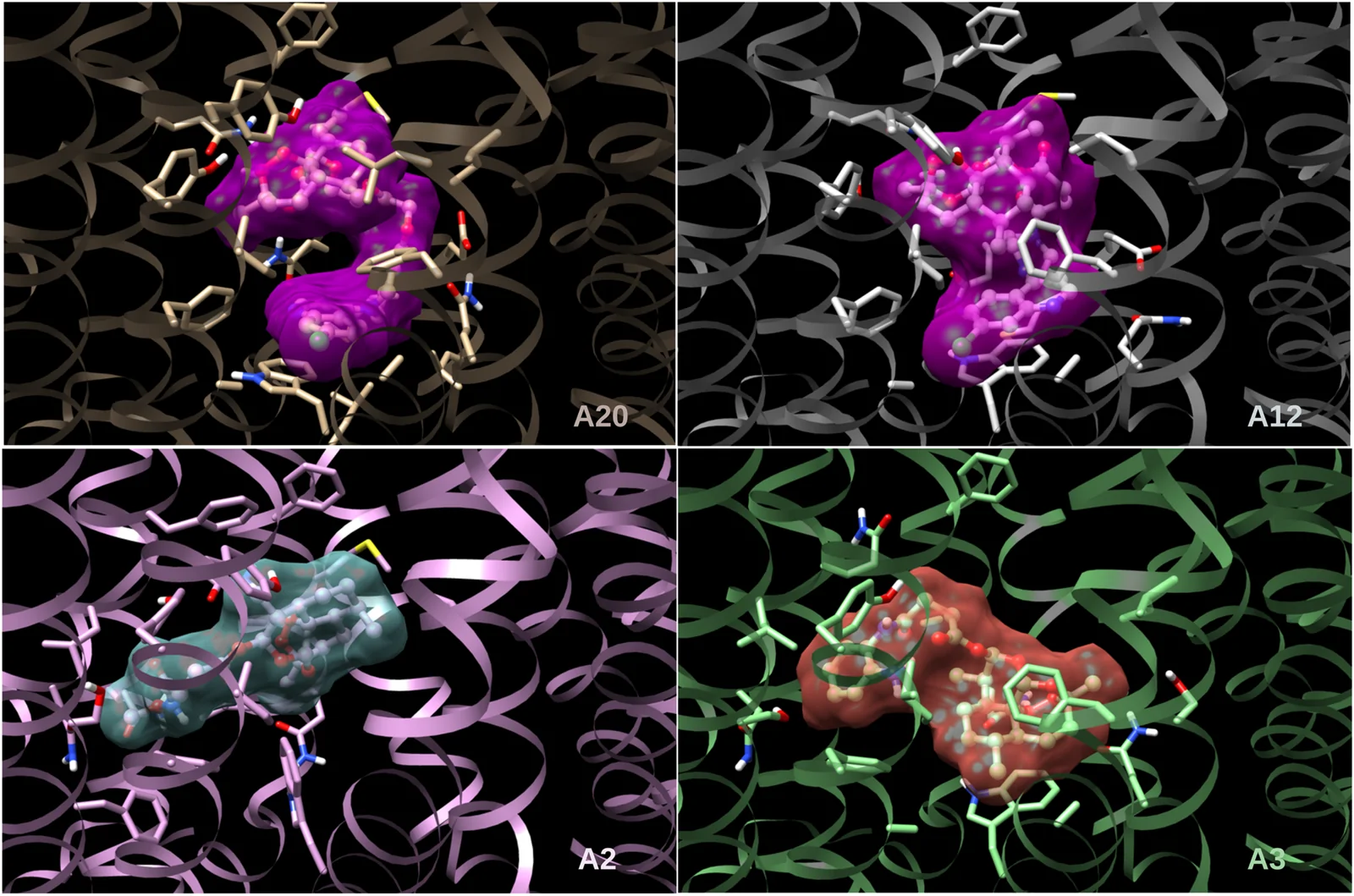
Comparison of the most populated cluster from the MD simulation for compounds A20 and A12, which exhibited favorable binding free energies 
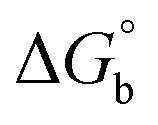
 values of −43.2 and −30.8 kcal mol^−1^, respectively with those of compounds A2 and A3 which showed less favorable values of −26.3 and −26.6 kcal mol^−1^. Note the pronounced changes in the shape and the loss of contacts with one of the two homologous halves of the protein (primarily involving TMH 6 and 12) relative to the more tightly bound compounds. In addition to their less favorable binding energies, compounds A2 and A3 failed to establish simultaneous tight interactions with the indicated transmembrane helices, a feature previously proposed to be essential for stable binding.

#### 3.4.2. QTAIM analysis confirms the key interactions underlying A16 and A20 binding

In order to go a step further, beyond the limitations of the classical force field used for computing the molecular dynamic (MD) trajectories, a multiscale QM/MM/QTAIM calculations on very large quantum-region models were prepared for non-active compound A2 as a negative control and for the most promising compounds A16 and A20. Analysis of the electron density at bond critical points (BCPs) provides a sensitive description of weak non-covalent interactions between ligand atoms and surrounding protein residues that are often underestimated by classical force fields. This methodology has been successfully applied to describe diverse biological systems.^37,68–71^ The QTAIM analysis of the representative structure of compound A20 is shown in Fig. 9 whereas the corresponding analyses for compounds A16 and A2 are available in Fig. S63 and S64, respectively. Bond paths corresponding to ligand–protein interactions are shown in yellow, with the associated BCPs highlighted in orange. The electron density values, *ρ*(*r*), measured at each BCP were summed to obtain Σ*ρ*(*r*), providing an estimate of the cumulative interaction strength for selected structural regions (Fig. 9). Aiming to gain a deeper insight into the contribution of each structural feature to the overall stabilization of the complex (in terms of Σ*ρ*), the molecules were conceptually divided into three fragments: (I) moiety 1 corresponding to the conserved artemisinin scaffold, (II) the flexible linker connecting artemisinin to the (III) moiety 2, representing the most variable part. The contribution of each fragment to the total electron density is summarized in Fig. 10. The significantly lower Σ*ρ*(*r*) value obtained for compound A2 agrees with its weaker binding affinity predicted by the classical MD simulations. More importantly, although the artemisinin scaffold is identical in all three compounds, its contribution to the total interaction density differs markedly. As discussed above (Fig. 7 and 8), only the most active compounds were able to accommodate the artemisinin scaffold deeply within the most hydrophobic region shaped by TMHs 4–6 together with the Met986 of TMH12. In compound A20 incorporation of the piperazine substituent into the quinoline moiety likely reduces direct π–π interactions while simultaneously enabling the flexible linker to establish tighter contacts with TMH12 and stabilize the characteristic twisted “U”-shaped binding conformation.

**Fig. 9:**
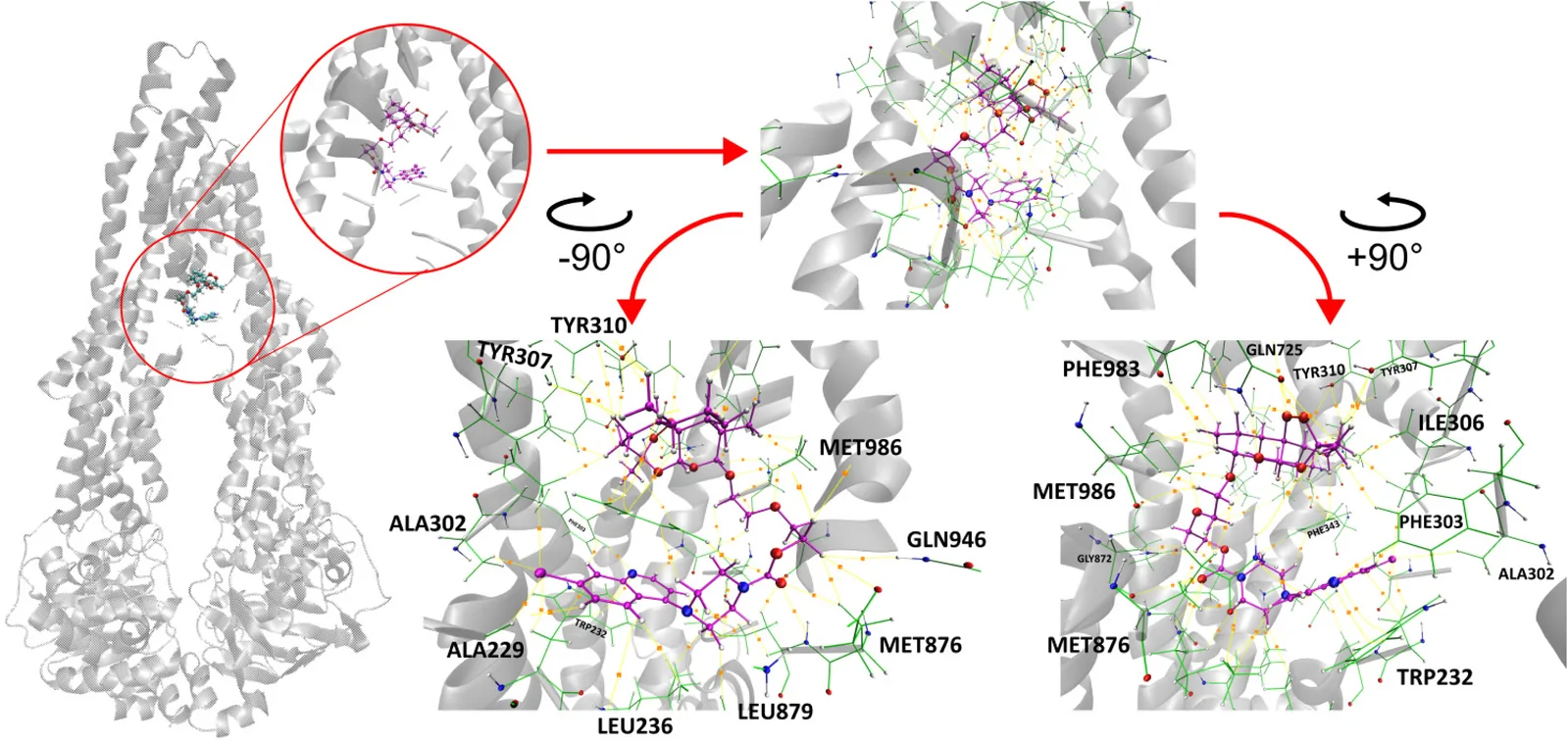
QM/MM/QTAIM analysis of the binding of compound A20 bound to the protein. The ligand is shown in violet and contacting residues within 5 Å in green. Bond paths and bond critical points (BCPs) corresponding to the non-covalent interactions are depicted as small orange spheres.

**Fig. 10:**
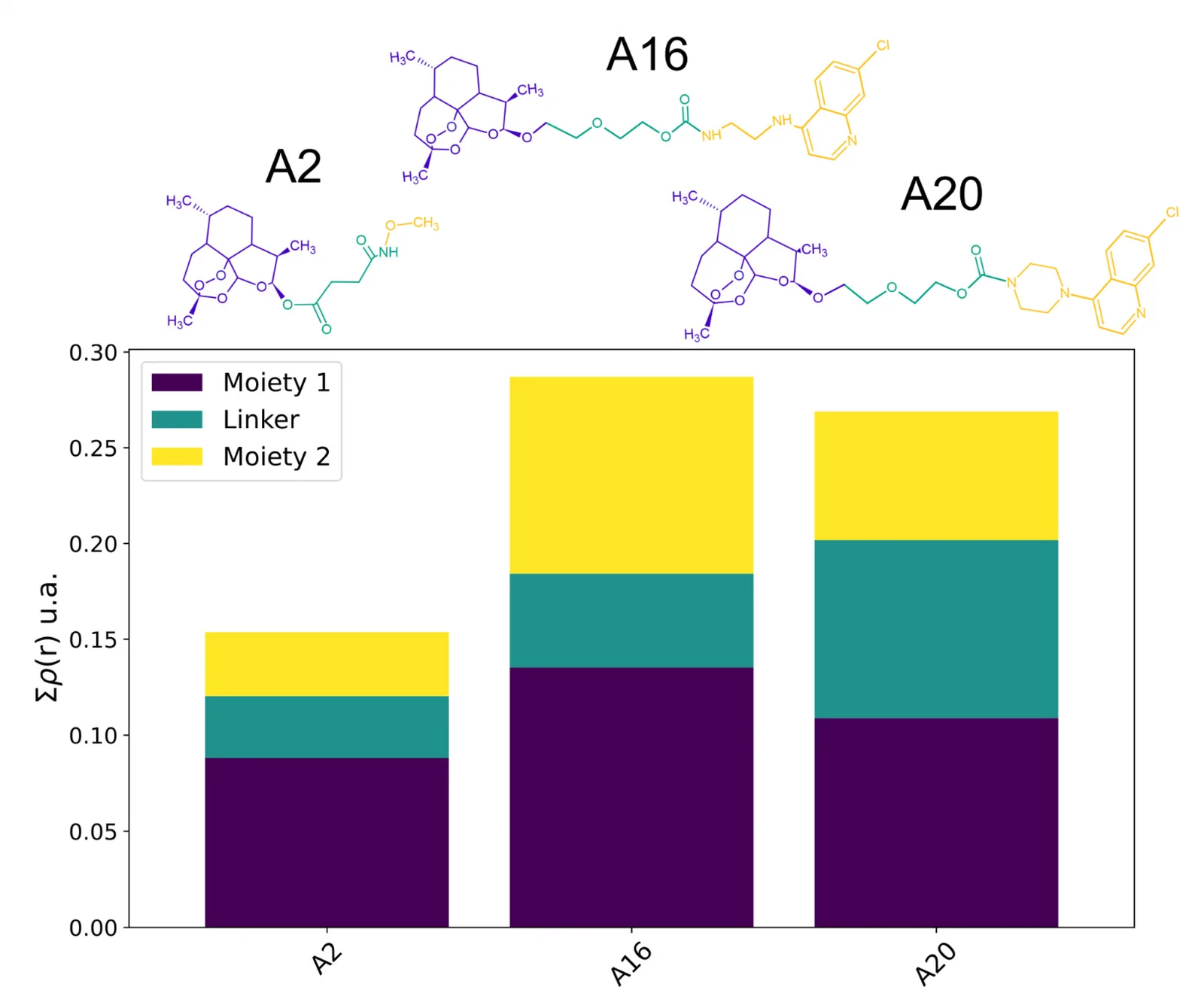
Decomposition of the contribution to the total Σ*ρ*(*r*) for compounds A2, A16 and A20.

An even more detailed description emerges from the residue-by-residue decomposition of the QTAIM electron density (Fig. 11). Overall, the interaction pattern closely resembles that obtained from the MM-PBSA decomposition (Fig. 6). However, interactions involving extended π systems and polarizable sulfur atoms are expected to be better reproduced. The contributions of Ala229 and Trp232 from TMH4 to the bindings of compounds A16 and A20 become more evident by this model, which was consistent with the highly polarizable and electron-rich character of the haloquinoline ring (Table S2). The extra contacts observed for compound A20 within residues Leu 879 and Met876 from TMH10, appear as the main difference with respect to compound A16 and resembles the interactions with tariquidar.^37^ Additional interactions observed exclusively for compound A20 with Leu879 and Met876 from TMH10 represent the main difference relative to compound A16 and resemble contacts previously reported for the potent P-gp inhibitor tariquidar.^37^ Likewise, the contributions of Leu339, Phe343, Gln347 from TMH6, Gln725 from TMH7, Phe983 and in particular Met986 from TMH12, clearly revealed a balance between the strength of the contacts involving each of the homologous halves (TMHs 4–6 and TMHs 7,12). This interaction pattern is consistent with that previously observed for the most active compounds from several chemically distinct families of P-gp modulators previously studied.^20,37,72^ In contrast, the absence of these key interactions in compound A2 is again evident from its substantially lower Σ*ρ*(*r*) values.

**Fig. 11:**
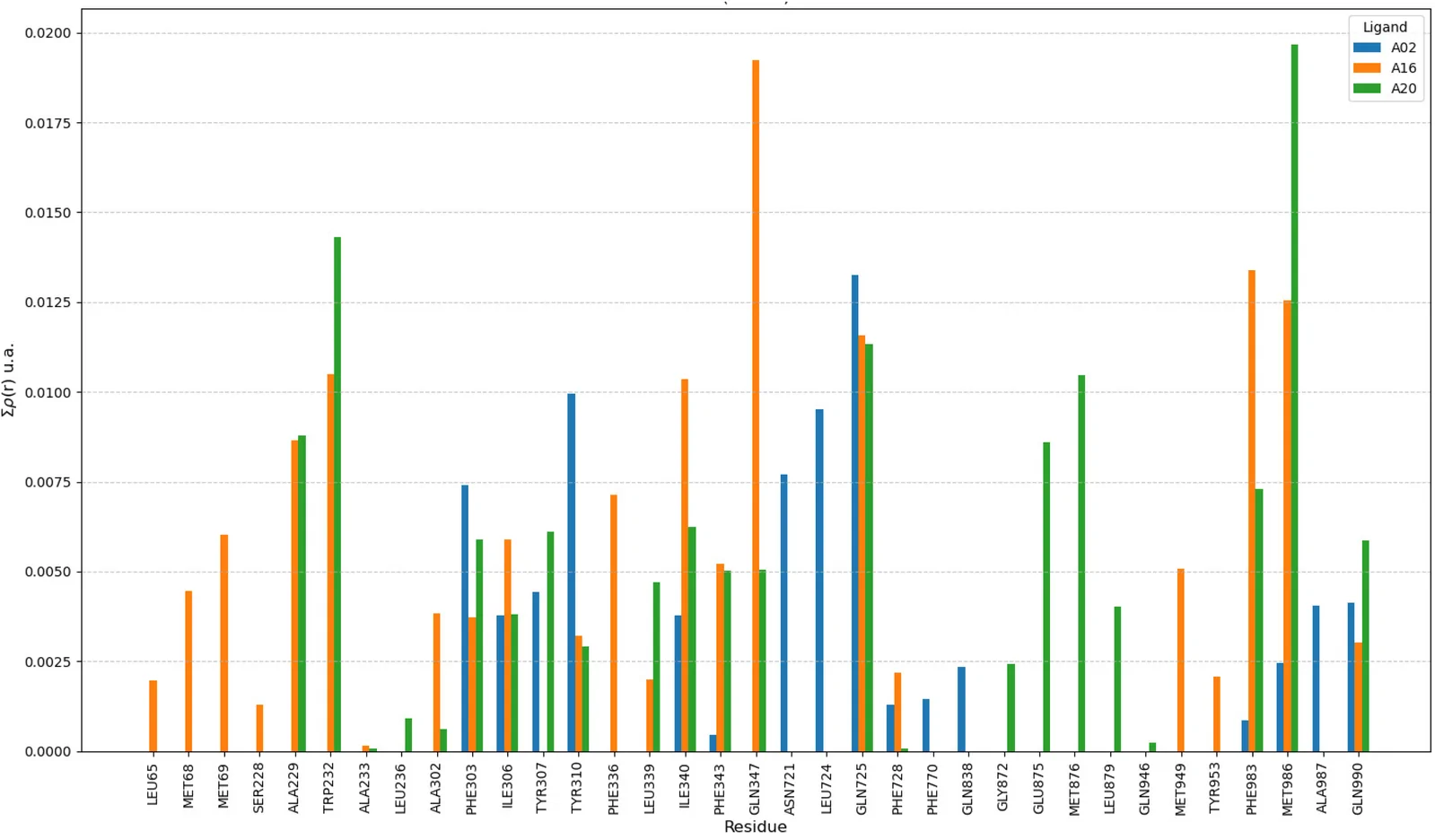
Sum of the electron densities at the bond critical points (BCPs) associated with the non-covalent interactions between the indicated residues and compounds A2 (blue), A16 (orange) and A20 (green).

## 4 Conclusions

A seris of artemisinin derivatives were designed and synthesized featuring linkers of varying lengths that connect the artemisinin core to either a methylbenzene group or nitrogen-containing heterocycles. Considering that many third-generation P-glycoprotein (P-gp) inhibitors, such as tariquidar, incorporate a tetrahydroisoquinoline scaffold,^17^ this structural moiety was selected and introduced as a secondary ring system in the designed compounds. Compounds A7, A12, A13, A14, A15, A16 and A20 demonstrated strong inhibition of P-gp-mediated Rho123 efflux. Among these, compounds A16 and A20 were the most potent, exhibiting low MEC values. Moreover, both compounds significantly increased the intracellular accumulation of Dox, thereby sensitizing K562/Dox to the cytotoxic drug at nanomolar concentrations. Importantly, neither compound affected the proliferation of normal PBMCs at the active concentrations, highlighting compounds A16 and A20 as promising scaffolds for overcoming P-gp-associated MDR. Despite the potential of P-gp inhibitors, previous clinical candidates have been limited by toxicity and drug–drug interactions.^73,74^ Although P-gp inhibition can effectively reverse MDR, systemic inhibition may also increase the exposure and toxicity of co-administered P-gp substrate drugs. Recent studies have highlighted the potential of natural products to alter drug exposure when co-administered with conventional medications, for example, Li *et al.* reported that co-administration of Chuanxiong and warfarin altered the pharmacokinetics of senkyunolide I.^74^ These findings emphasize the importance of carefully evaluating the pharmacokinetic, safety, and interaction profiles of compounds A16 and A20 to maximize MDR reversal with potential drug–drug interaction risks and establish their translational feasibility. Structural analysis revealed that the presence of a quinoline moiety and an extended linker length enhanced anti-P-gp activity. Similarly, incorporating three *O*-methyl groups further improved inhibitory potency, consistent with previous reports.^20,37^ Molecular dynamics simulations support the hypothesis that the modulating activity of these artemisinin derivatives is closely related to their ability to adopt a compact and stable conformation within the P-gp binding pocket. In particular, maintaining the characteristic “U”-shaped conformation, persistent interactions with key residues, and simultaneous occupation of both hydrophobic and polar regions appear to be critical determinants distinguishing active from inactive compounds. Notably, interactions occurring near TMH12, a region functionally linked to the catalytic domains, suggest a possible influence on the conformational transitions that regulate transport activity. This observation may provide a useful basis for future large-scale simulations of related systems.

The agreement between calculated binding energies, predicted interaction patterns, and experimental data underscores the importance of integrating modeling and simulation approaches to rationalize biological activity. These findings not only deepen our understanding of the mechanism of action of the most promising derivatives but also establish concrete structural criteria to guide the rational design of novel P-gp modulators with enhanced selectivity and efficacy. Furthermore, detailed binding mode analyses, now complemented by first-principles DFT and multiscale calculations, confirm the hypotheses proposed in previous studies^20,37^ regarding the key structural determinants required for potent modulating activity.

## Ethical statement

All experiments were performed in accordance with the international ethical principles established in the Declaration of Helsinki and to Good Clinical Practice (GCP) guidelines. It also complies with Provincial Law No. 9694 on Health Research of the Province of Córdoba, Resolution No. 1644 of the Ministry of Health of the Province of Córdoba, and ANMATP rovisions No. 1480/2011 and No. 7516/2025, as applicable to research involving human subjects. Personal data are processed in accordance with National Law No. 25,326 on the Protection of Personal Data and Law No. 26,529 on Patients' Rights. Experiments were approved by the ethics committee at Institutional Committee for Ethics in Health Research (Comité Institucional de Ética de Investigación en Salud-CIEIS), Clínica Universitaria Reina Fabiola, Universidad Católica de Córdoba. Informed consents were obtained from human participants of this study.

## Author contributions

Jerónimo Laiolo: investigation, formal analysis. Artemis Tsirogianni: investigation, formal analysis. Cecilia L. Barbieri: software, formal analysis. Mariana Belén Joray: investigation. Dafni G. Graikioti: investigation, formal analysis, writing – editing. Dimitra Toumpa: investigation. Jazmín Lavallén: investigation. Nicolás A. Rodriguez: software, formal analysis. D. Mariano A. Vera: writing – original draft, writing – editing, funding acquisition, formal analysis, software, data curation. Constantinos M. Athanassopoulos: supervision, writing – original draft, funding acquisition, formal analysis, conceptualization, data curation. María Cecilia Carpinella: supervision, writing – original draft, writing – editing, funding acquisition, formal analysis, conceptualization, data curation.

## Conflicts of interest

The authors declare no competing interests.

## Abbreviations

COMU: (((*Z*)-(1-cyano-2-ethoxy-2-oxoethylidene)amino)oxy)-*N*,*N*-methyl(morpholin-4-yl)methaniminium hexafluorophosphate
DFT: density functional theory
DIPEA: *N*,*N*-diisopropylethylamine
DMAP: 4-(dimethylamino) pyridine
DMF: dimethylformamide
DMSO: dimethyl sulfoxide
EDCI: ethyldimethylaminopropyl carbodiimide hydrochloride
HBTU: [Dimethylamino-(3-oxidobenzotriazol-3-ium-1-yl)methylidene]-dimethylazanium hexafluorophosphate
HOBt: 1-hydroxybenzotriazole
MDR: multidrug resistance
QTAIM: quantum theory of atoms in molecules

## Supplementary Material

RA-016-D6RA07164C-s001

## Data Availability

All data supporting this study are included in the article and its supplementary information (SI). Additional data are available from the corresponding author upon reasonable request. Supplementary information is available. See DOI: https://doi.org/10.1039/d6ra07164c.
