# Supplementary material for: Design, synthesis and biological evaluation of novel artemisinin derivatives as potent P-glycoprotein modulators for overcoming multidrug resistance
Source: RSC Adv. 2026 Oct 26. Online ahead of print. doi: 10.1039/d6ra07164c (PMC13702068; doi:10.1039/d6ra07164c)
Supplement: Supplementary file 1 [file RA-OLF-D6RA07164C-s001.pdf]

Supplementary Information (SI) for RSC Advances.
This journal is © The Royal Society of Chemistry

Supplementary Data

Design, synthesis and biological evaluation of novel artemisinin derivatives as potent P-glycoprotein inhibitors for overcoming multidrug resistance

Jerónimo Laiolo[a, e, †], Artemis Tsirogianni[b, †], Cecilia L. Barbieri[c, †], Mariana Belén Joray[d], Dafni G. Graikioti[b], Dimitra Toumpa[b,f], Jazmín Lavallén[d], Nicolás A. Rodriguez[c], D. Mariano A. Vera[c, ***], Constantinos M. Athanassopoulos[b, **], María C. Carpinella[d, *]

[a] *Universidad Católica de Córdoba, Córdoba, Argentina.*

[b] *Synthetic Organic Chemistry Laboratory, Department of Chemistry, University of Patras, GR-26504, Patras, Greece.*

[c] *Department of Chemistry and Biochemistry, College of Exact and Natural Sciences, Universidad Nacional de Mar del Plata - QUIAMM – INBIOTEC CONICET, Mar del Plata, Argentina*

[d] *Fine Chemical and Natural Products Laboratory, CIDIE CONICET-UCC, Universidad Católica de Córdoba, Córdoba, Argentina.*

* Corresponding author. Email: ceciliacarpinella@ucc.edu.ar (M.C. Carpinella).

** Corresponding author. Email: kath@upatras.gr (C. M. Athanassopoulos).

*** Corresponding author. Email: dmavera@yahoo.com (D.M.A. Vera).

† Equal first author contribution.

[e] Present address: *Instituto de Investigación Médica Mercedes y Martín Ferreyra, Consejo Nacional de Investigaciones Científicas y Técnicas (INIMEC-CONICET), Universidad Nacional de Córdoba, Córdoba 5016, Argentina.*

[f] Present address: *Laboratory for Advanced Fibers, Empa, Swiss Federal Laboratories for Materials Science and Technology, Lerchenfeldstrasse 5, 9014, St. Gallen, Switzerland* (DT).

Table S1. MDR-reversing activity of less active artemisinin derivatives and inactive derivatives in K562/Dox cells

Compounds	FAR						
	K562/Dox				K562		
	Concentration (μM)				Concentration (μM)		
	50	25	12.5	6.25	50	25	12.5
artemisinin	1.09±0.08				0.98±0.05		
artesunate	0.77±0.01				0.86±0.06		
DHA	0.84±0.005				0.80±0.01***		
A2	1.02±0.01				0.92±0.07		
A4	1.02±0.02				0.85±0.07		
A5	1.07±0.06				0.88±0.04		
A10	0.86±0.01				0.99±0.01		
A19	0.66±0.04				0.81±0.01***		
A3	1.28±0.04***	1.04±0.09			0.98±0.04		
A18	1.26±0.05**	0.83±0.08			0.81±0.03*		
A11	1.31±0.06*	0.80±0.04*			0.88±0.05*		
A1	1.40±0.08**	1.20±0.06*	1.11±0.04*	1.00±0.03		0.82±0.04*	
A6	1.85±0.15**	1.24±0.07**	1.11±0.01*	0.99±0.02	0.84±0.04*		

A8	3.75±0.43***	2.58±0.15**	**1.42±0.08***	1.02±0.03	0.90±0.05
A17	2.64±0.18**	1.99±0.18*	**1.45±0.01***	0.96±0.07	0.87±0.05

Fluorescence activity ratio (FAR) = mean fluorescence intensity (MFI) of rhodamine 123 (Rho123) with compound/MFI of Rho123 alone. DHA: dihydroartemisinin. The minimum effective concentrations (MECs) of each compound are shown in bold. Significant differences between treated and untreated cells were determined by using the one-tailed paired Student´s t-test ($^{***}p < 0.001$, $^{**}p < 0.01$, $^{*}p < 0.05$).

Experimental procedures

Synthesis of key intermediates

Synthesis of alcohol **A21**

To an ice-cold (0 °C) solution of DHA (0.23 g, 0.8 mmol) and diethylene glycol in CH_2Cl_2 (7 mL), $BF_3{\cdot}Et_2O$ was added dropwise, and the reaction mixture was stirred at room temperature for 1.5 h. After the completion of the reaction, the mixture was diluted with CH_2Cl_2 and was washed with $NaHCO_3$, H_2O and brine. The organic layer was dried over Na_2SO_4, filtered, and evaporated to dryness under reduced pressure. The residue was then subjected to FCC using PhMe/EtOAc 1:1 as eluent, affording corresponding alcohol **A21** in 72% yield; yellow oil; R_f (PhMe/EtOAc 1:1)=0.21; ^1H NMR ($CDCl_3$, 600 MHz) δ 5.31 (d, *J*=24.8 Hz, 1H),

4.49 (d, *J*=9.3 Hz, 1H), 4.06 – 4.01 (m, 1H), 3.73 – 3.69 (m, 4H), 3.61 (t, *J*=6 Hz, 2H), 2.45 – 2.41 (m, 1H), 2.39 – 2.34 (m, 1H), 2.01 (dq, *J*=14.0 Hz, 4.5 Hz, 1H), 1.90 – 1.86 (m, 1H), 1.78 – 1.74 (m, 1H), 1.70 – 1.67 (m, 1H), 1.56 – 1.52 (m, 1H), 1.49 – 1.46 (m, 1H), 1.43 (s, 2H), 1.28 – 1.25 (m, 2H), 0.95 (d, *J*=6.0 Hz, 3H), 0.89 (d, *J*=7.1 Hz, 3H) ppm; ^{13}C NMR ($CDCl_3$, 151 MHz) δ 104.3, 100.4, 91.2, 80.3, 72.3, 70.4, 68.2, 61.7, 52.6, 51.6, 45.3, 37.4, 36.3, 34.2, 32.4, 26.0, 24.7, 22.1, 20.2, 12.5 ppm; ESI-MS (30eV) m/z: 395.45 $[M+Na]^+$, 411.38 $[M+K]^+$.

Activation of alcohol **A21**

To an ice-cold (0 °C) solution of alcohol **A21** and 4-nitro-phenyl-chloroformate in CH_2Cl_2, Et_3N was added dropwise, and the reaction mixture was stirred at room temperature for 24h. After the completion of the reaction, the mixture was diluted with CH_2Cl_2 and was washed with 5% aqueous citric acid, H_2O and brine. The organic layer was dried over Na_2SO_4, filtered, and evaporated to dryness under reduced pressure. The residue was then subjected to FCC using PhMe/EtOAc 9:1 as eluent, affording corresponding activated alcohol **A22** in 80% yield; yellow oil; R_f (PhMe/EtOAc 9:1)=0.26; ^{1}H NMR ($CDCl_3$, 600 MHz) δ 8.28 (d, *J*=9.2 Hz, 2H), 7.39 (d, *J*=9.2 Hz, 2H), 5.38 (d, *J*=2.8 Hz, 1H), 4.51 (d, *J*=3.6 Hz, 1H), 4.43 (t, *J*=4.6 Hz, 2H), 4.07 – 4.05 (m, 1H), 3.83 – 3.82 (m, 2H), 3.75 – 3.70 (m, 3H), 2.44 – 2.40 (m, 1H), 2.38 – 2.25 (m, 1H), 2.04 – 2.00 (m, 1H), 1.89 – 1.85 (m, 1H), 1.76 – 1.73 (m, 1H), 1.68 – 1.65 (m, 1H), 1.56 – 1.52 (m, 1H), 1.43 (s, 3H), 1.29 – 1.24 (m, 3H), 1.02 – 0.98 (m, 1H) 0.93 (d, *J*=6.1 Hz, 3H), 0.89 (d, *J*=7.1 Hz, 3H) ppm; ^{13}CNMR ($CDCl_3$, 151 MHz) δ 155.5, 145.4, 125.3,

121.8, 104.3, 100.3, 91.2, 80.3, 70.8, 68.6, 68.1, 51.6, 45.3, 37.3, 36.3, 34.2, 32.5, 26.0, 24.7, 22.1, 20.2, 12.5 ppm; ESI-MS (30eV) m/z: 560.27 $[M+Na]^+$, 576.33 $[M+K]^+$.

Experimental NMR spectra

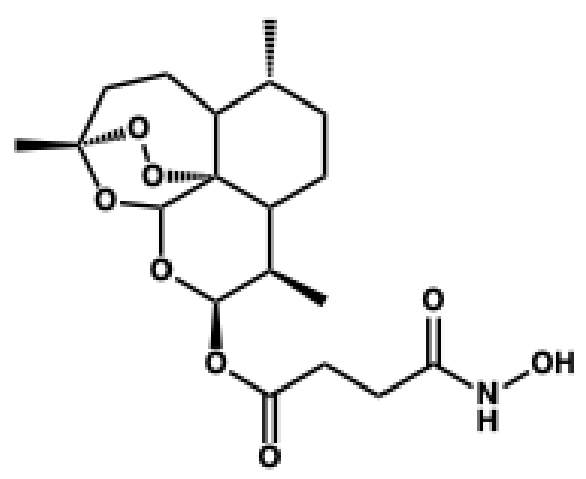

A1

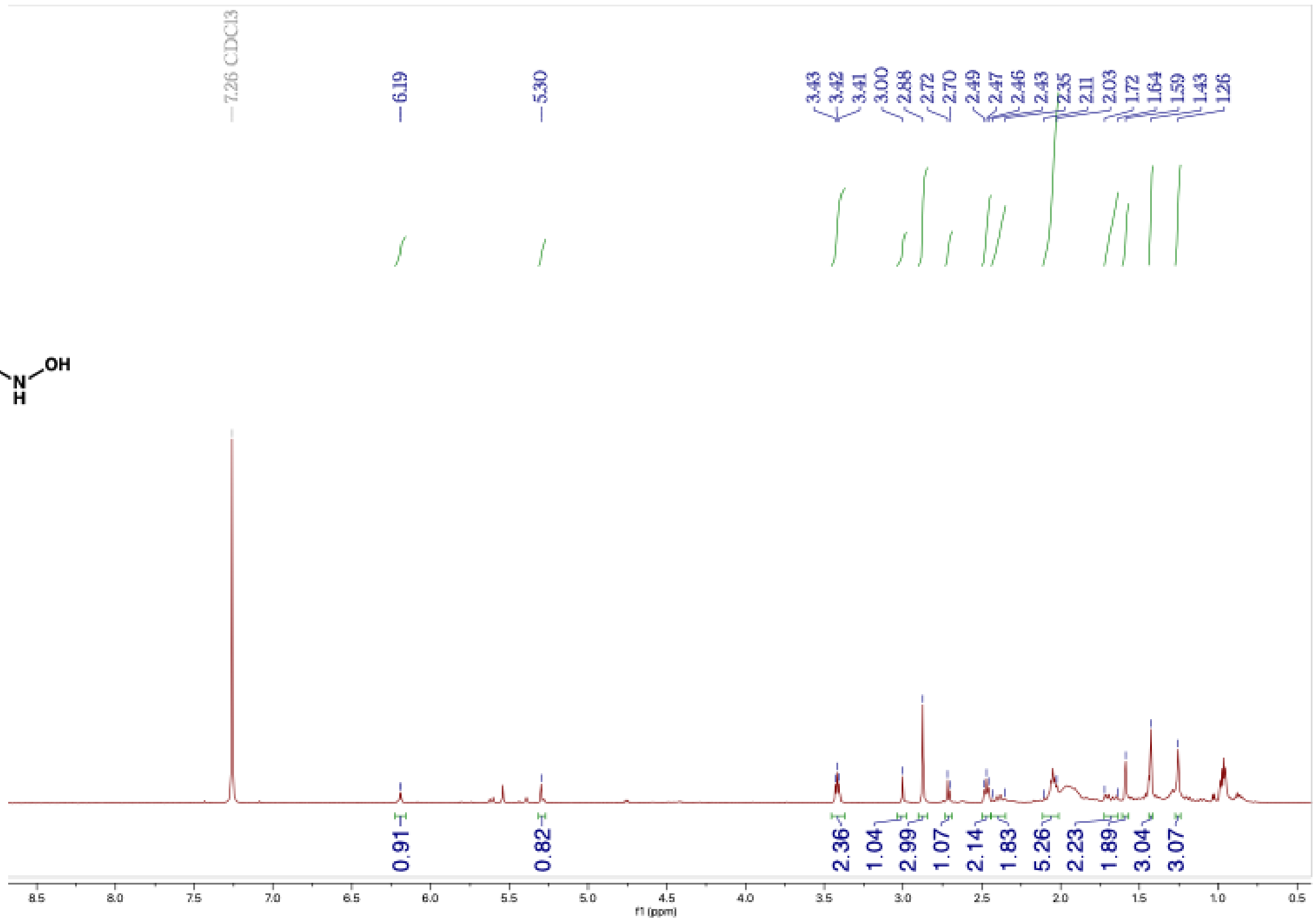

Fig. S1. ^{1}H NMR spectrum of compound **A1**.

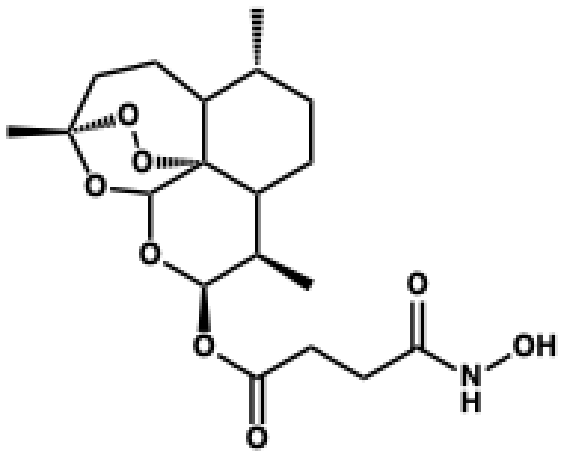

A1

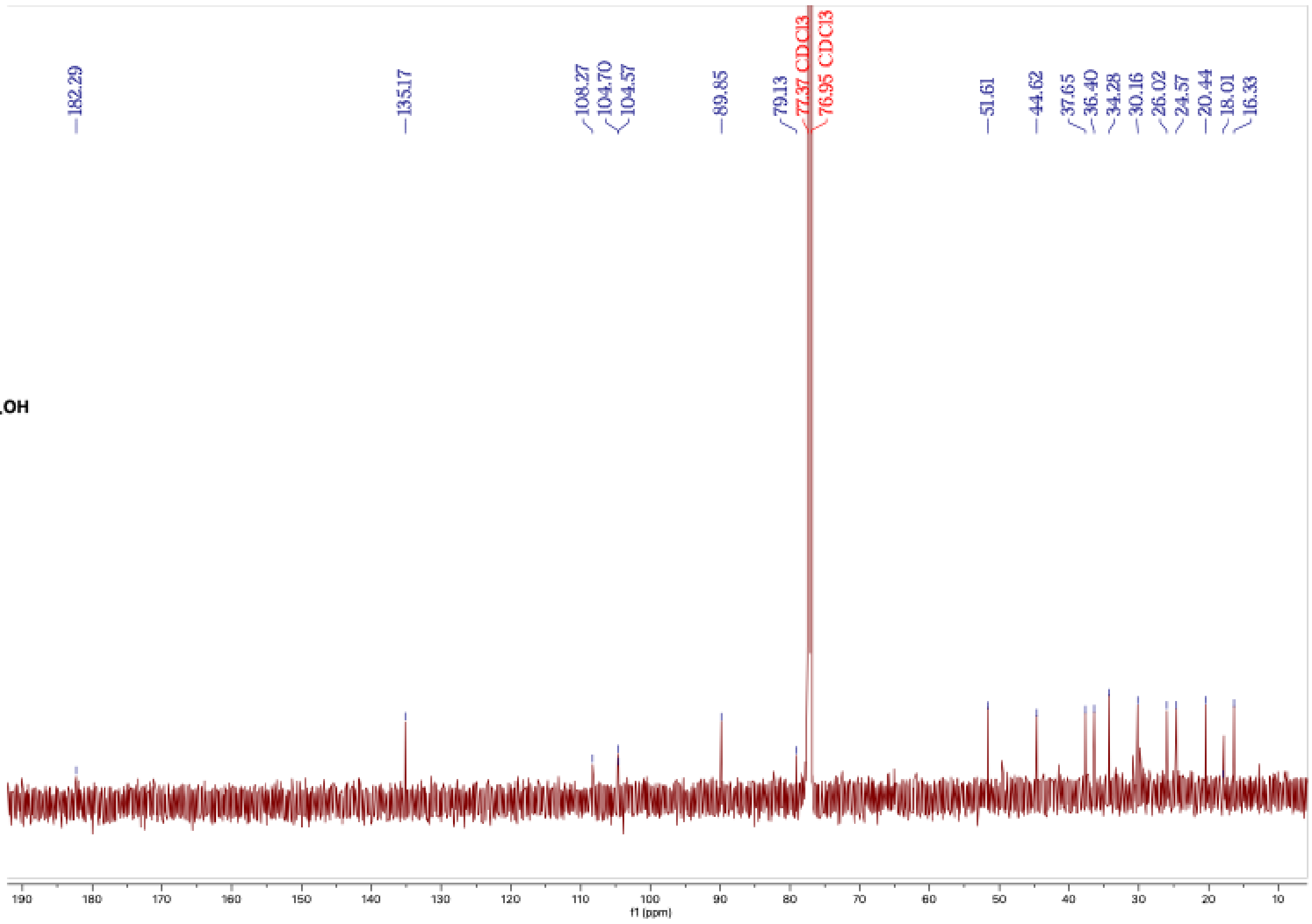

Fig. S2. ^{13}C NMR spectrum of compound **A1**.

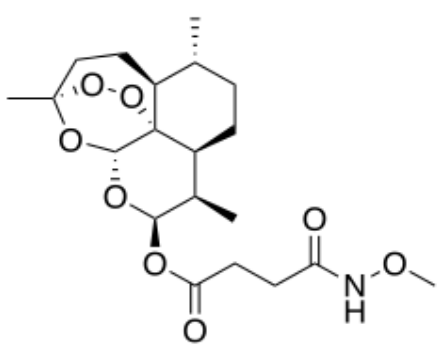

A2

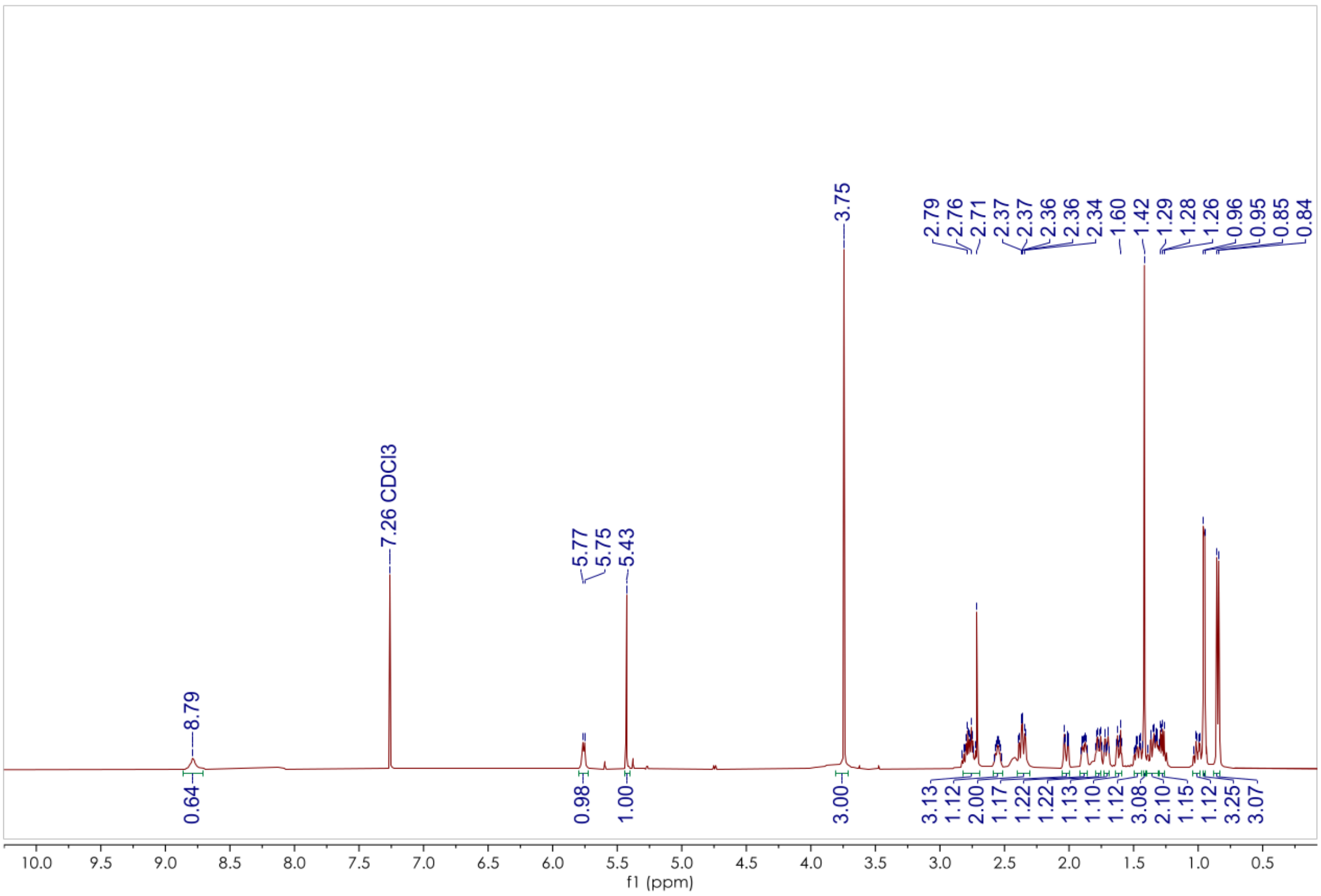

Fig. S3. ^{1}H NMR spectrum of compound **A2**.

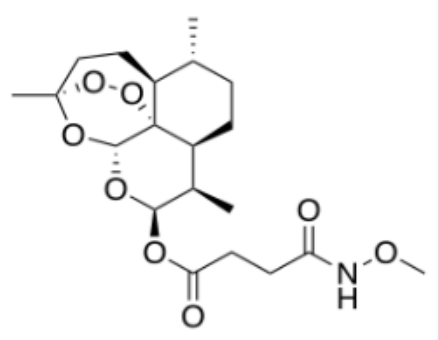

A2

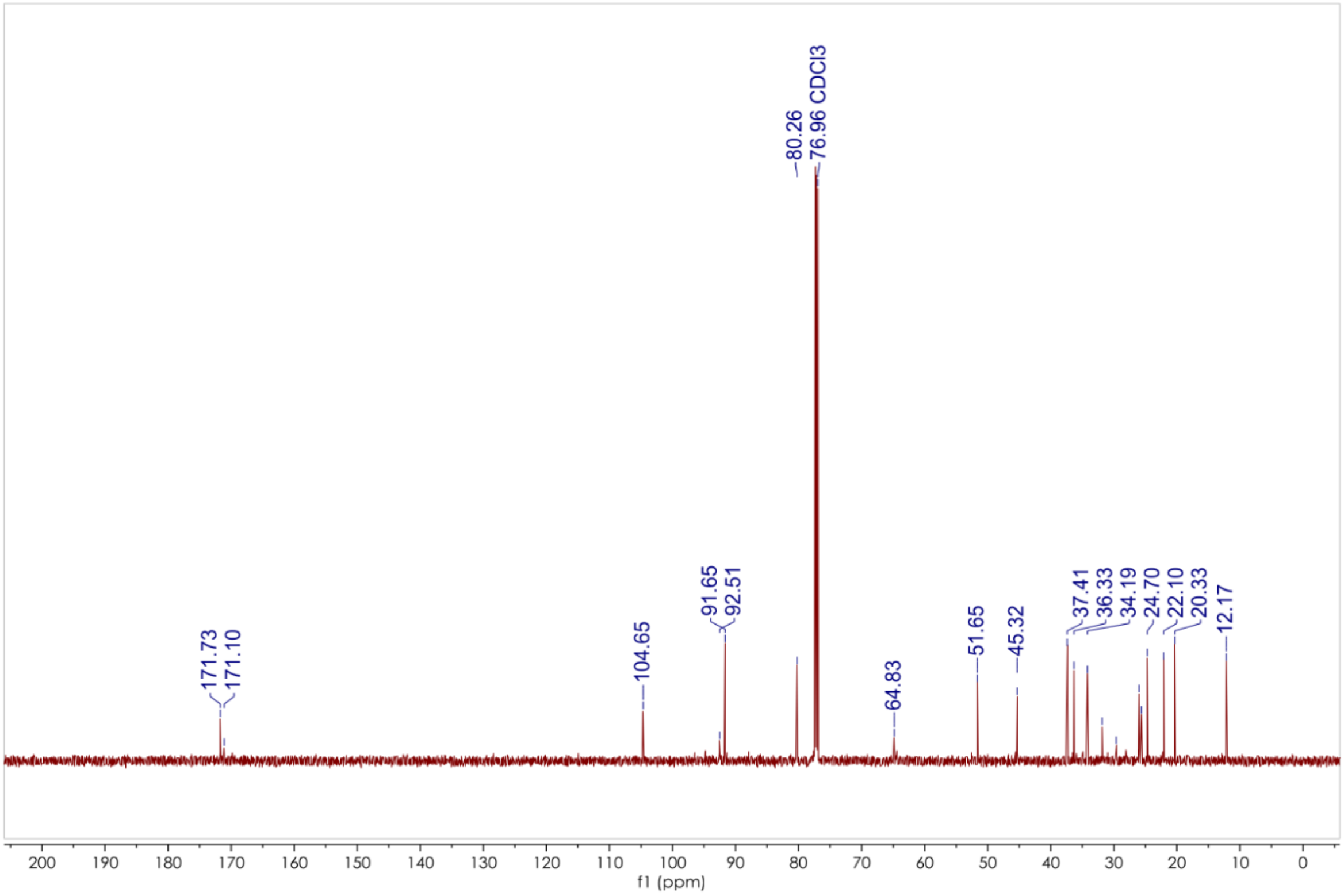

Fig. S4. ^{13}C NMR spectrum of compound **A2**.

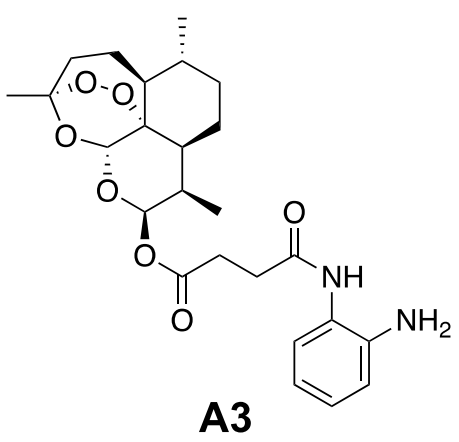

A3

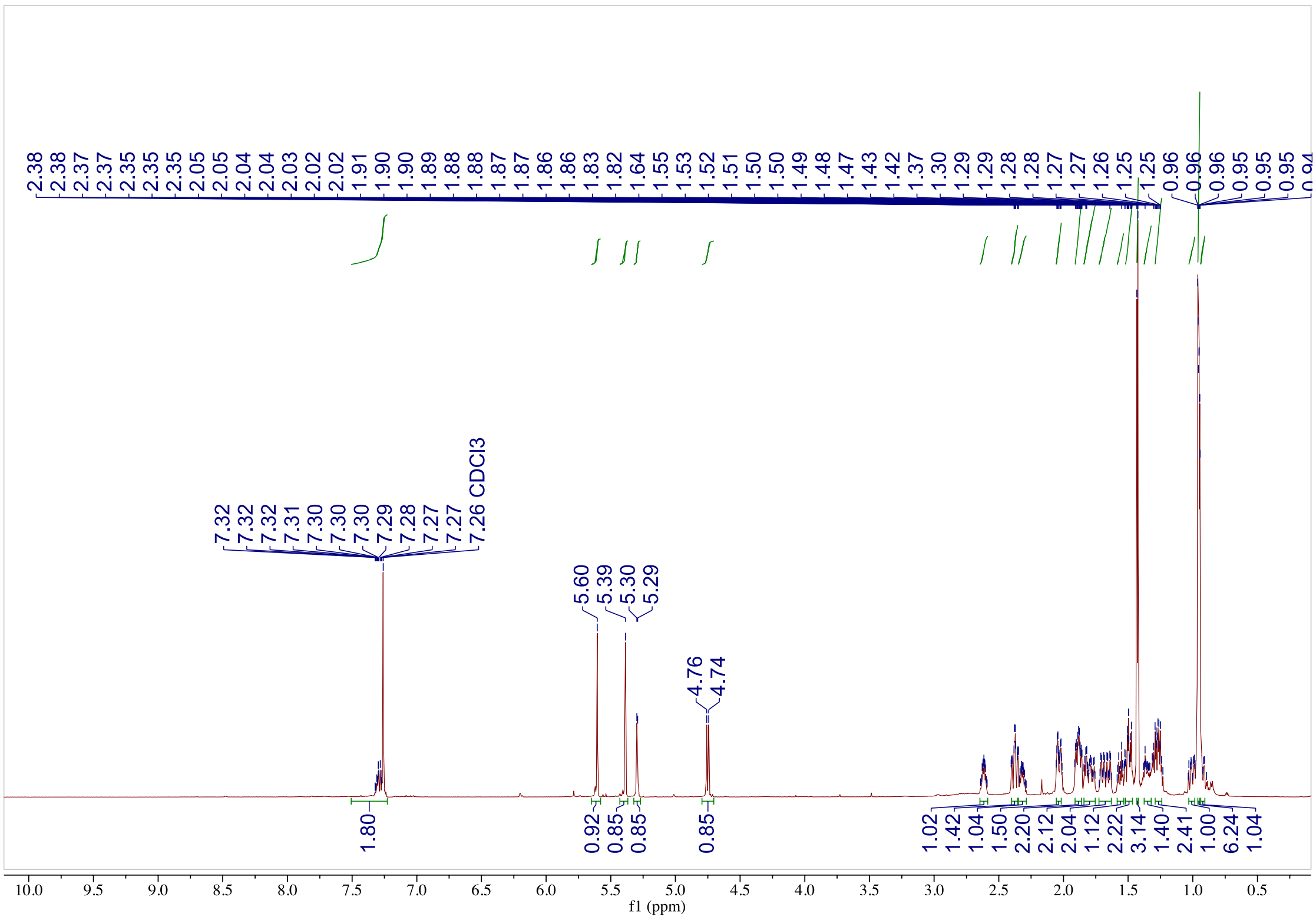

Fig. S5. ^{1}H NMR spectrum of compound **A3**.

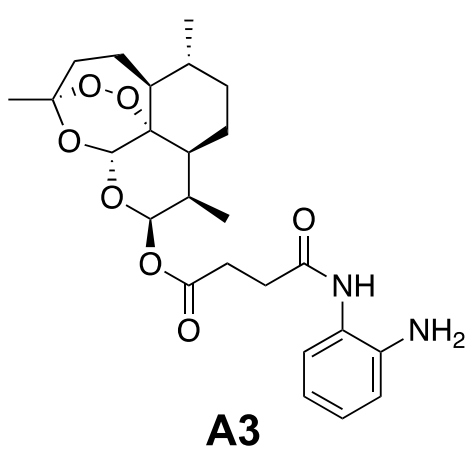

A3

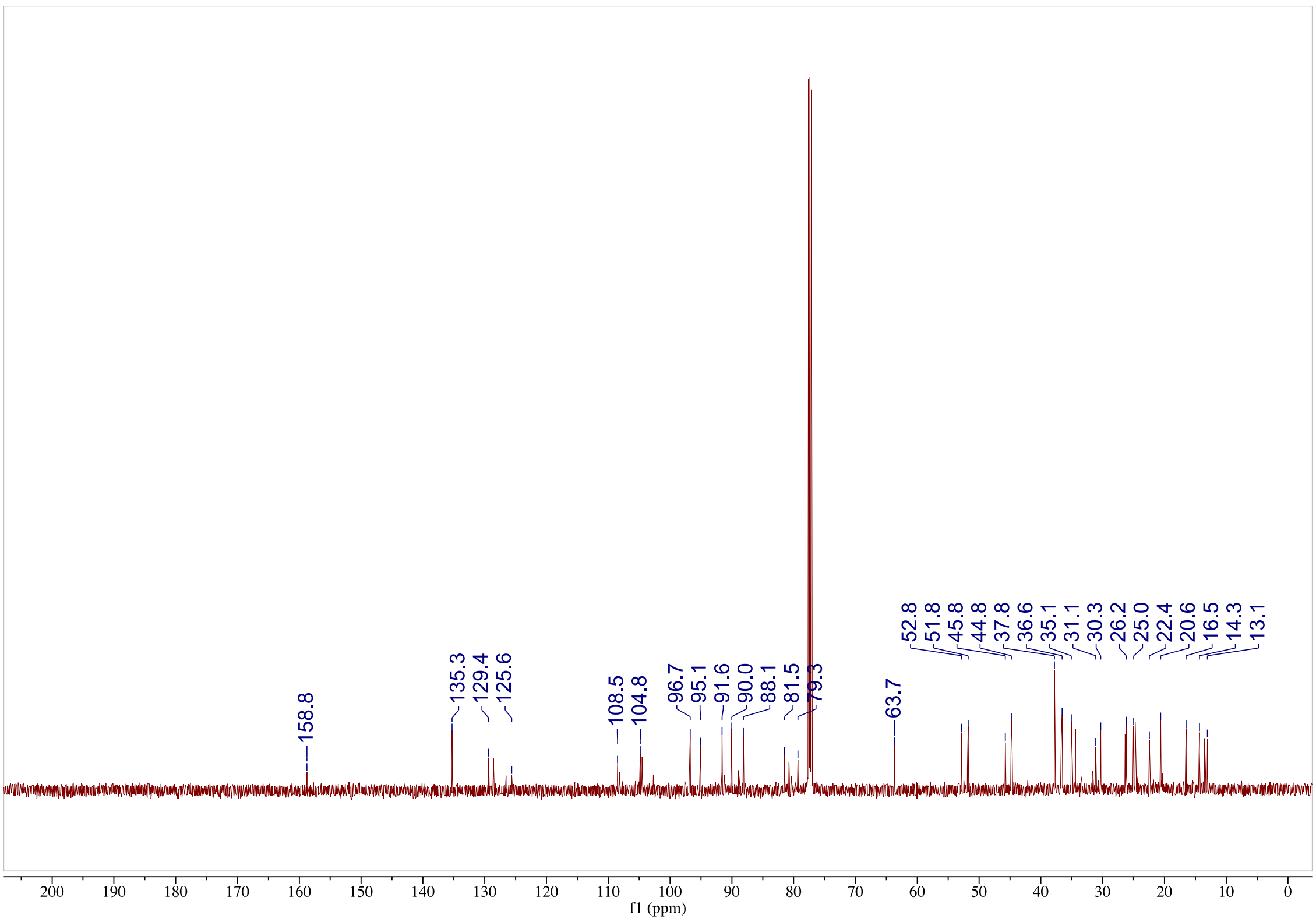

Fig. S6. ^{13}C NMR spectrum of compound **A3**.

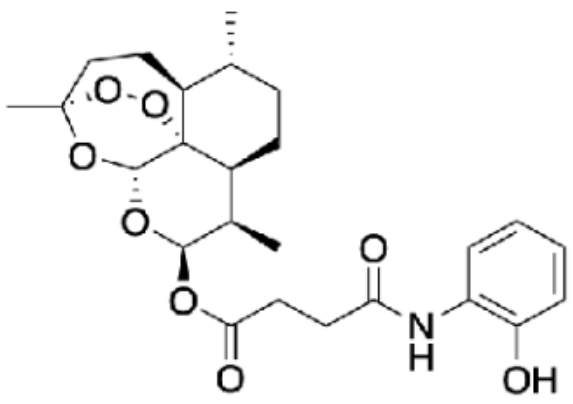

A4

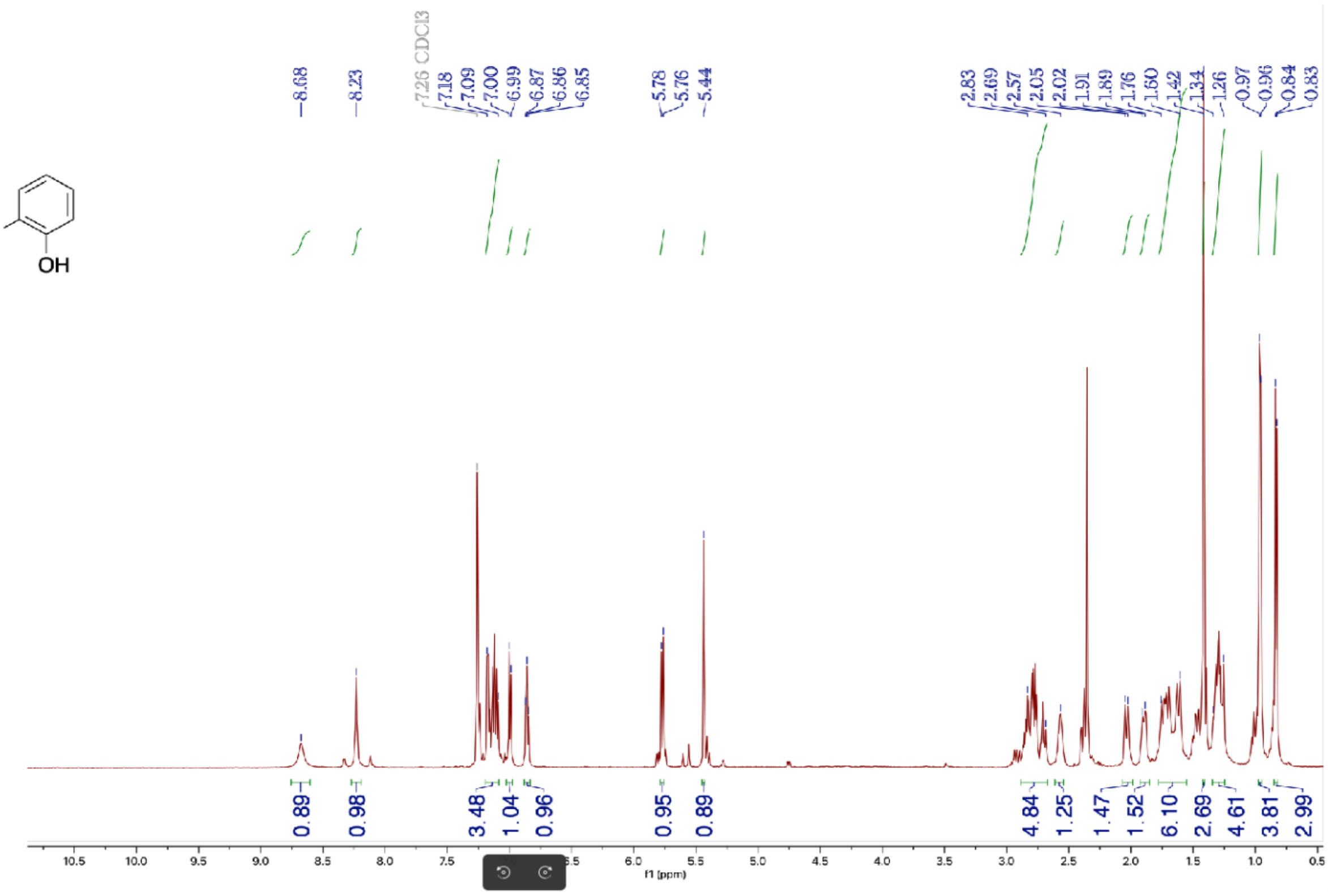

8.68
8.23
7.26 CDCl3
7.18
7.09
7.00
6.99
6.87
6.86
6.85
5.78
5.76
5.44
2.83
2.69
2.57
2.05
2.02
1.91
1.89
1.76
1.60
1.42
1.34
1.26
0.97
0.96
0.84
0.83
0.89
0.98
3.48
1.04
0.96
0.95
0.89
4.84
1.25
1.47
1.52
6.10
2.69
4.61
3.81
2.99
f1 (ppm)

Fig. S7. ^{1}H NMR spectrum of compound **A4**.

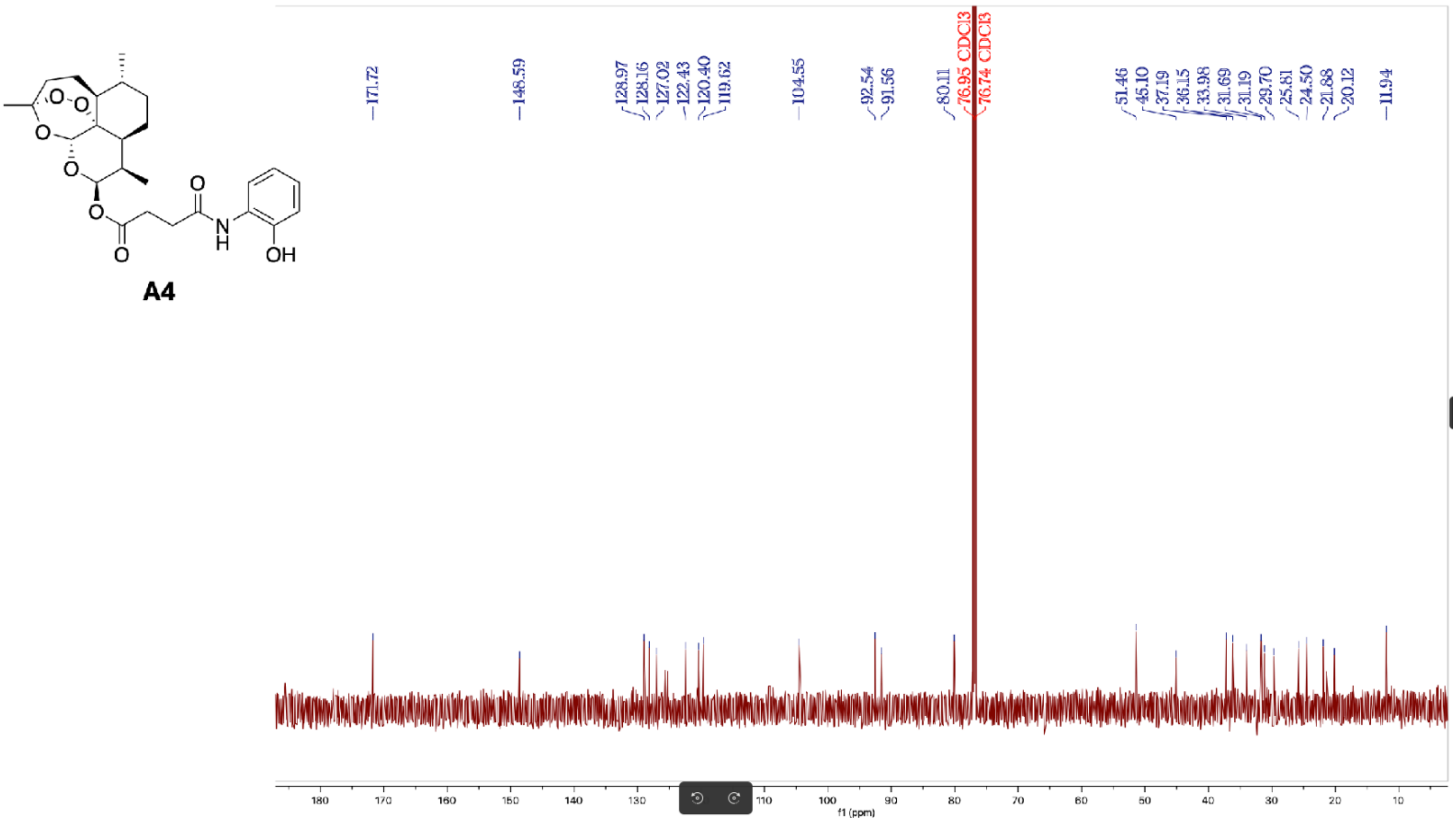

Fig. S8. ^{13}C NMR spectrum of compound **A4**.

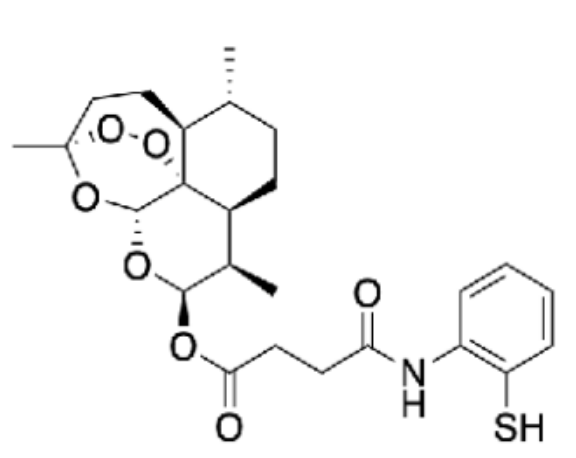

A5

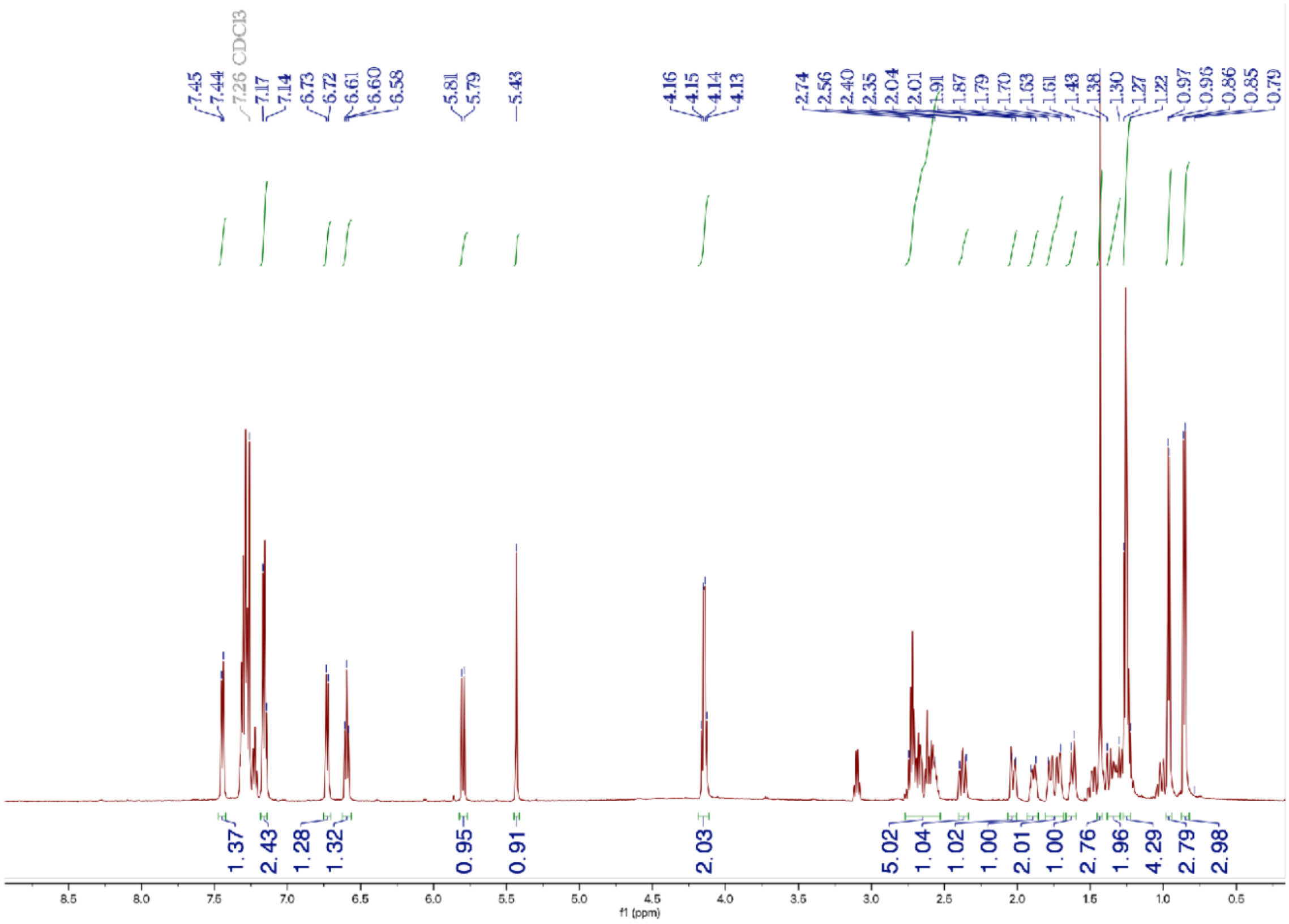

7.45
7.44
7.26 CDCl3
7.17
7.14
6.73
6.72
6.61
6.60
6.58
5.81
5.79
5.43
4.16
4.15
4.14
4.13
2.74
2.56
2.40
2.35
2.04
2.01
1.91
1.87
1.79
1.70
1.63
1.61
1.43
1.38
1.30
1.27
1.22
0.97
0.96
0.86
0.85
0.79
1.37
2.43
1.28
1.32
0.95
0.91
2.03
5.02
1.04
1.02
1.00
2.01
1.00
2.76
1.96
4.29
2.79
2.98
f1 (ppm)

Fig. S9. ^{1}H NMR spectrum of compound **A5**.

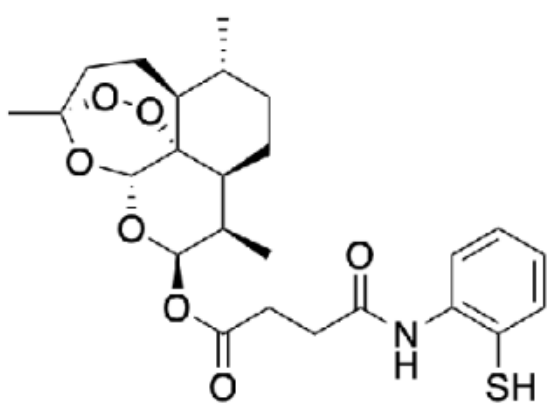

A5

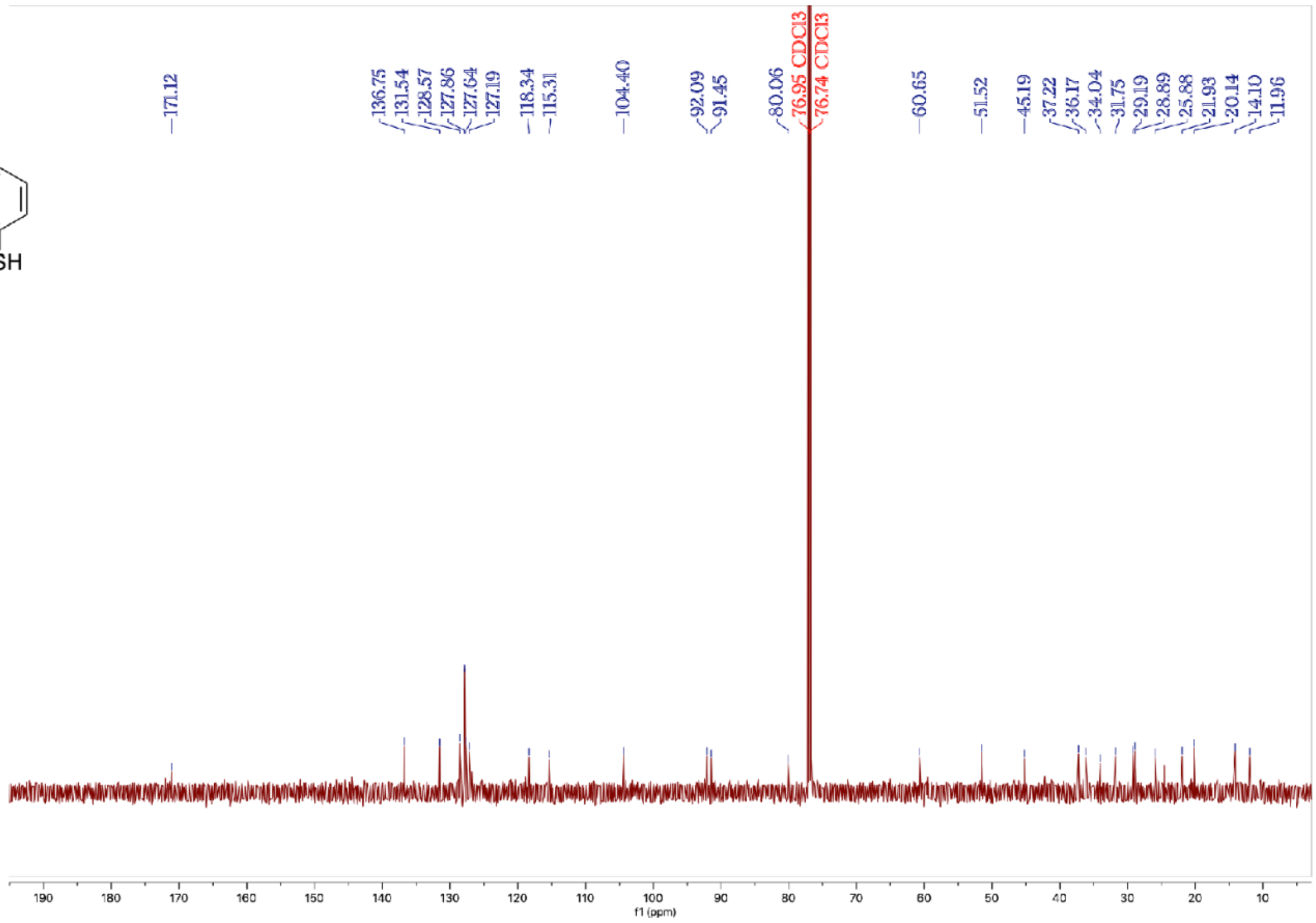

Fig. S10. ^{13}C NMR spectrum of compound **A5**.

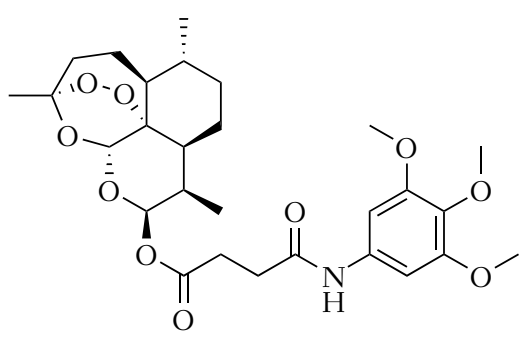

A6

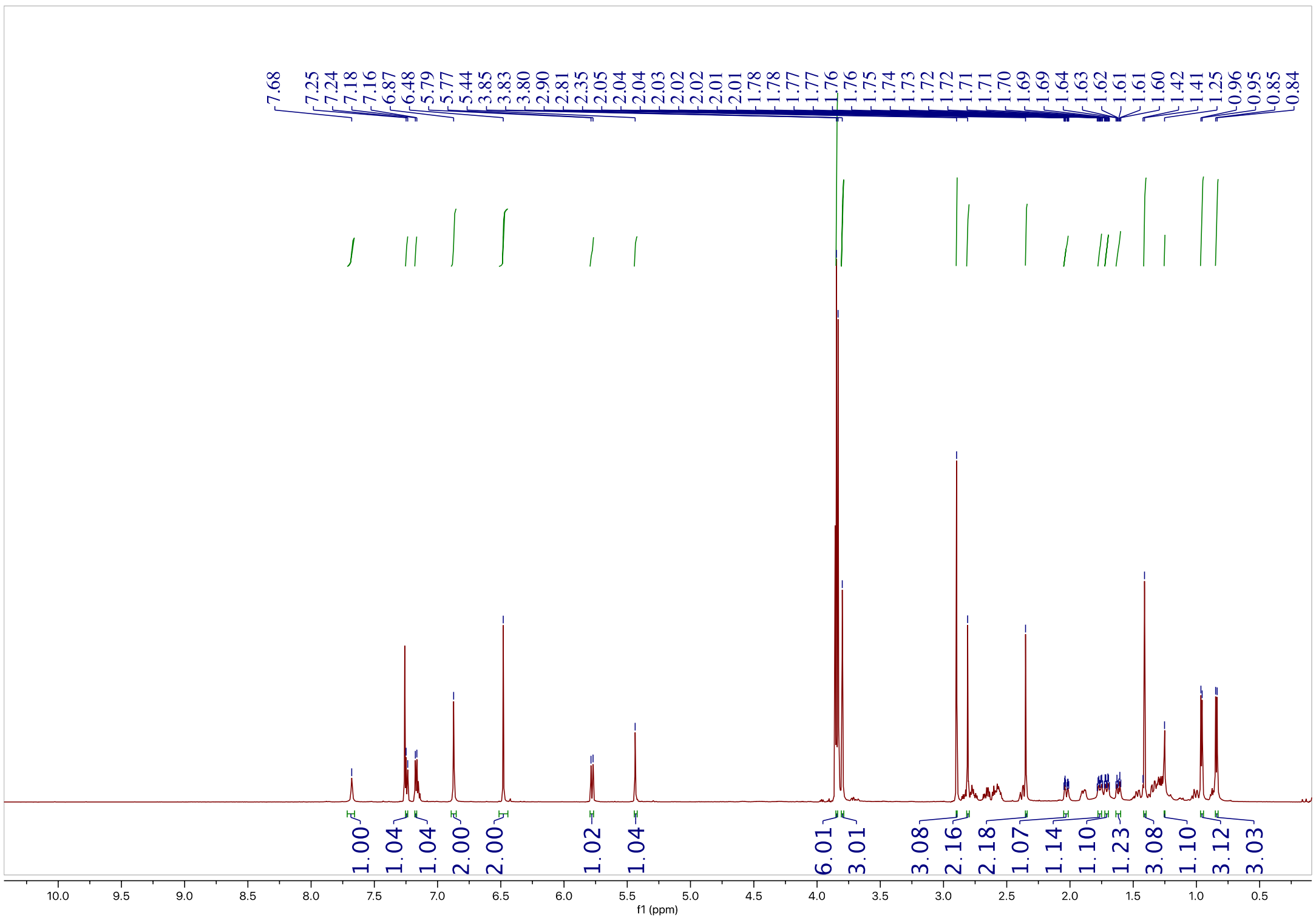

Fig. S11. ¹H NMR spectrum of compound **A6**.

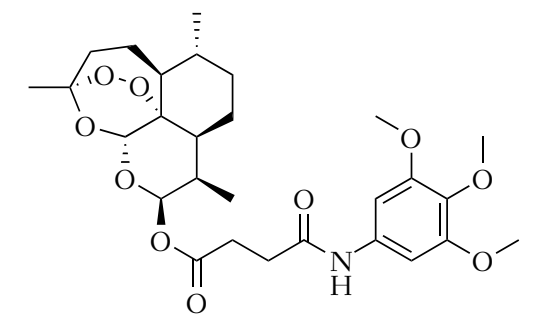

A6

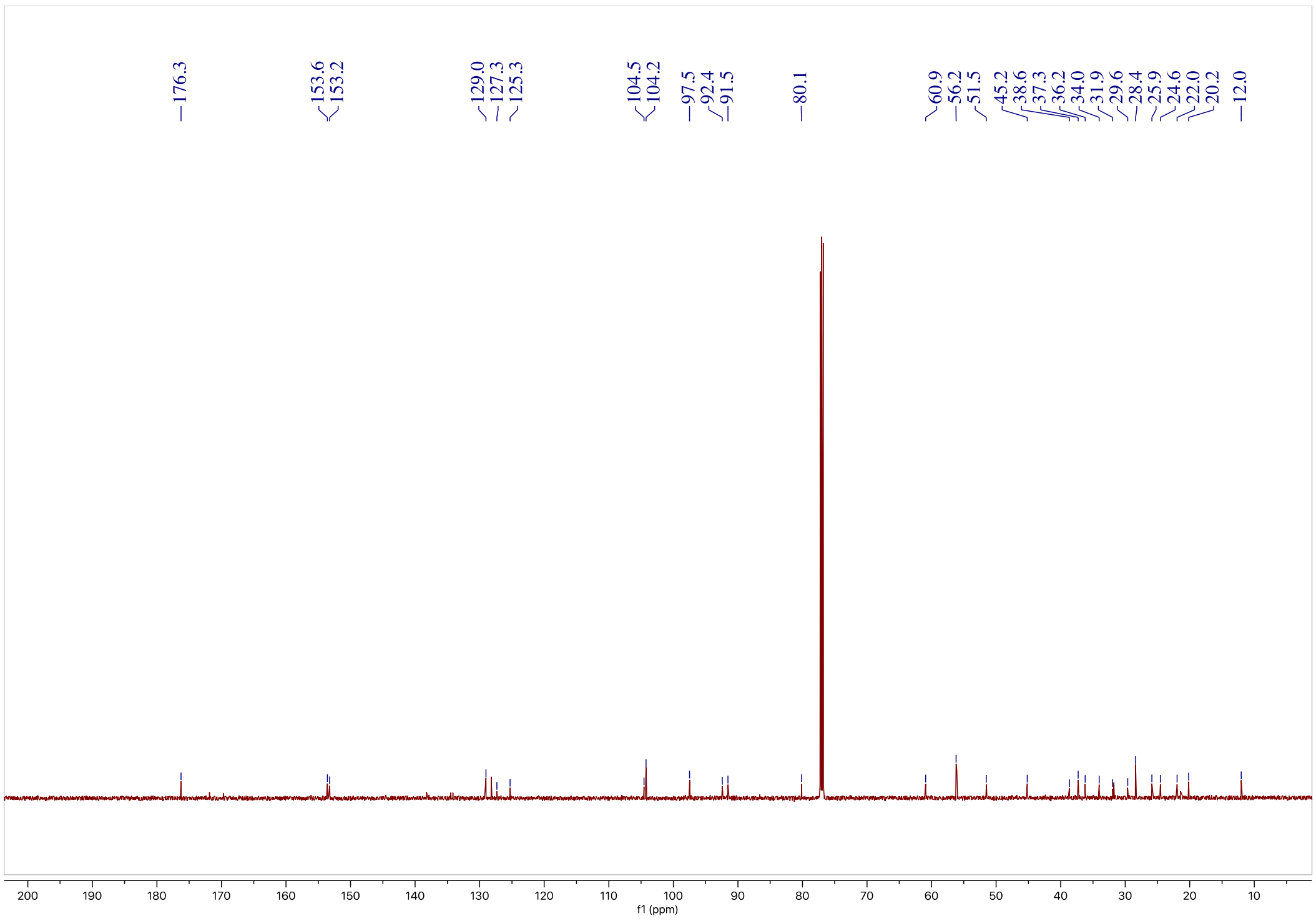

Fig. S12. ¹³C NMR spectrum of compound **A6**.

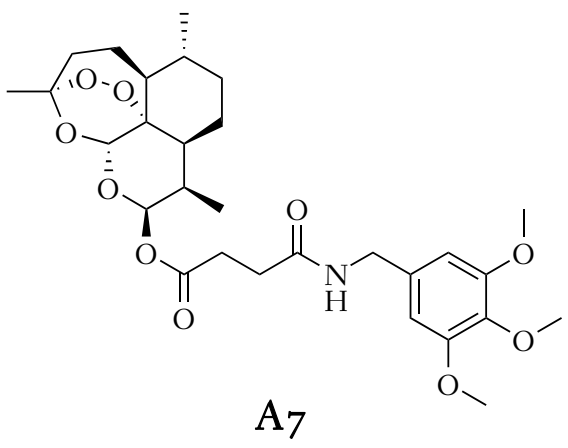

A7

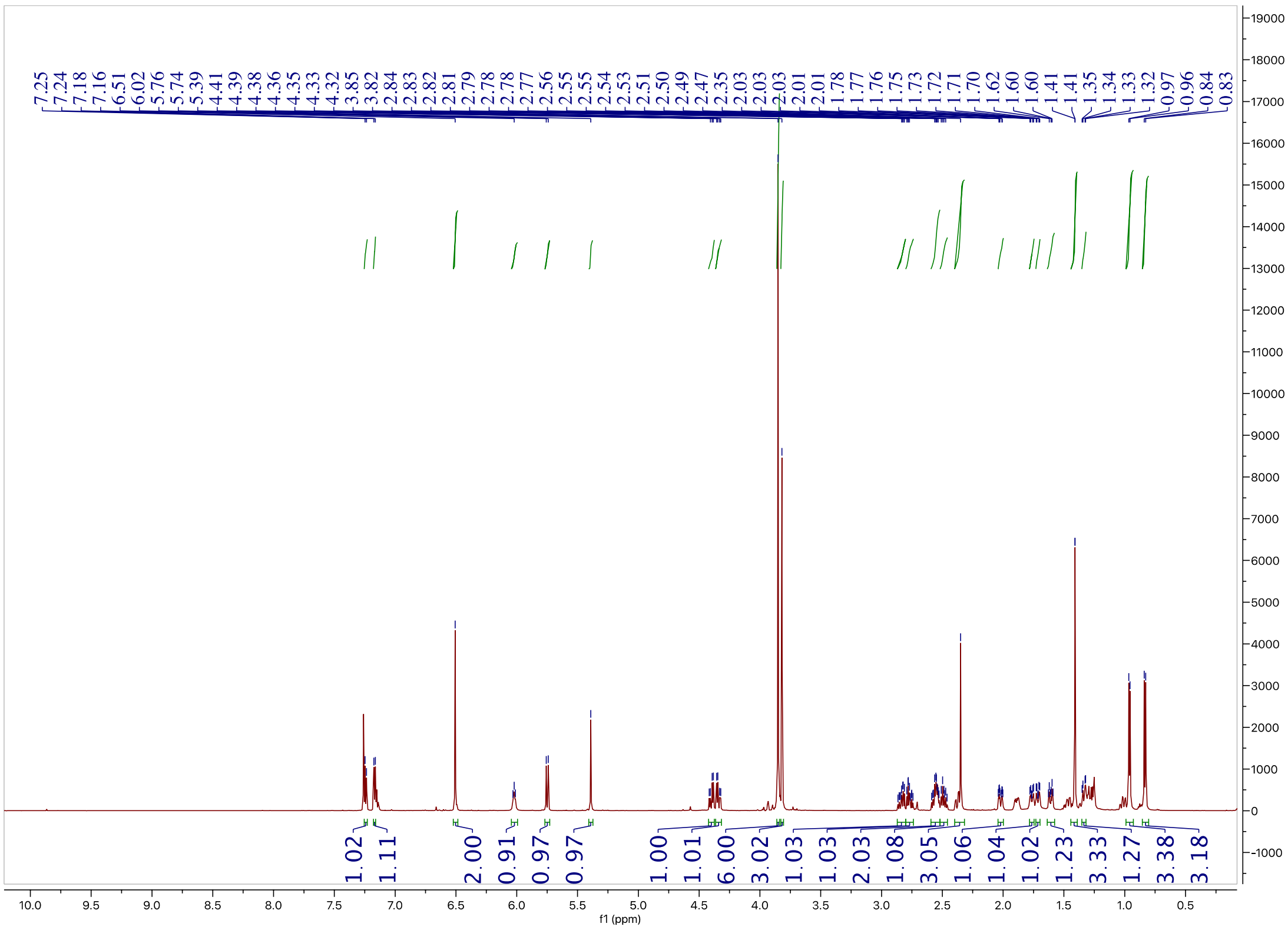
7.25 7.24 7.18 7.16 6.51 6.02 5.76 5.74 5.39 4.41 4.39 4.38 4.36 4.35 4.33 4.32 3.85 3.82 2.84 2.83 2.82 2.81 2.79 2.78 2.77 2.56 2.55 2.55 2.54 2.53 2.51 2.50 2.49 2.47 2.35 2.03 2.03 2.03 2.01 2.01 1.78 1.77 1.76 1.75 1.73 1.72 1.71 1.70 1.62 1.60 1.60 1.41 1.41 1.35 1.34 1.33 1.32 0.97 0.96 0.84 0.83
1.02 1.11 2.00 0.91 0.97 0.97 1.00 1.01 6.00 3.02 1.03 1.03 2.03 1.08 3.05 1.06 1.04 1.02 1.23 3.33 1.27 3.38 3.18
f1 (ppm)

Fig. S13. ^{1}H NMR spectrum of compound **A7**.

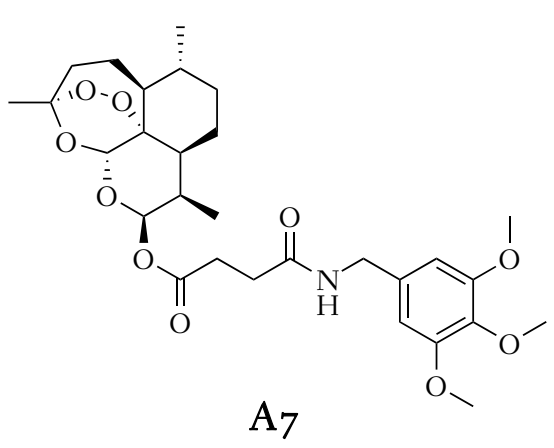

A7

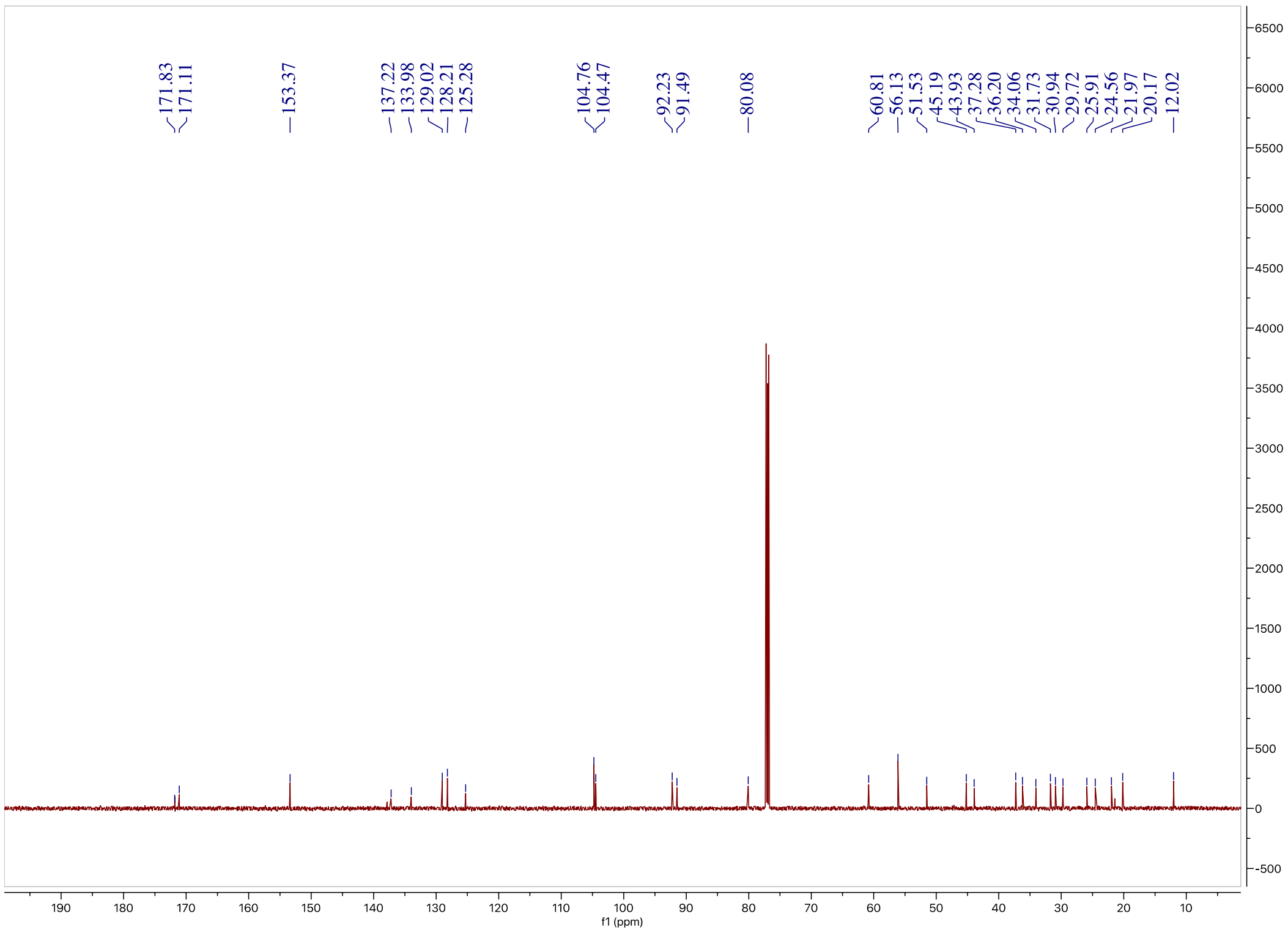

Fig. S14. ^{13}C NMR spectrum of compound **A7**.

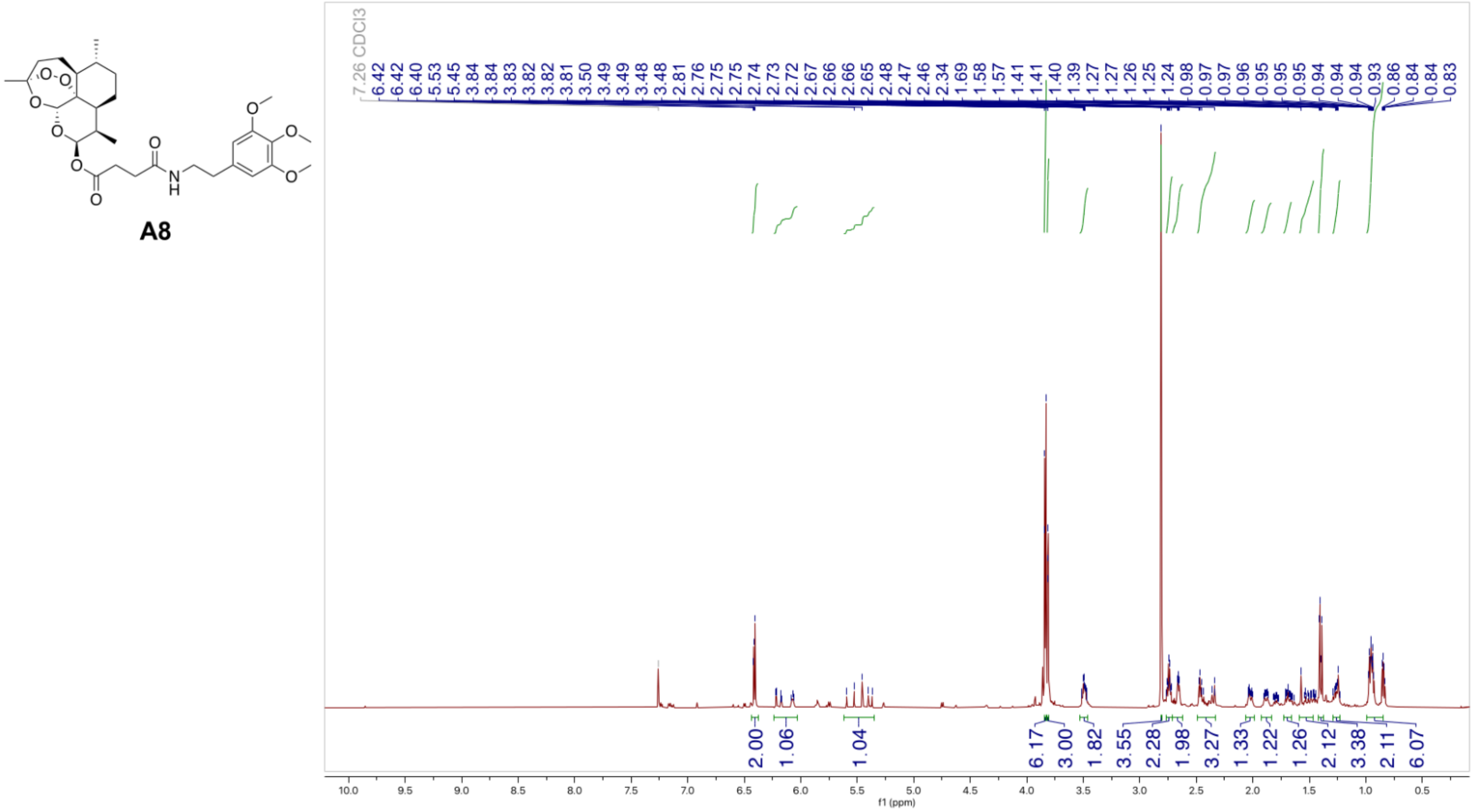

Fig. S15. ^{1}H NMR spectrum of compound **A8**.

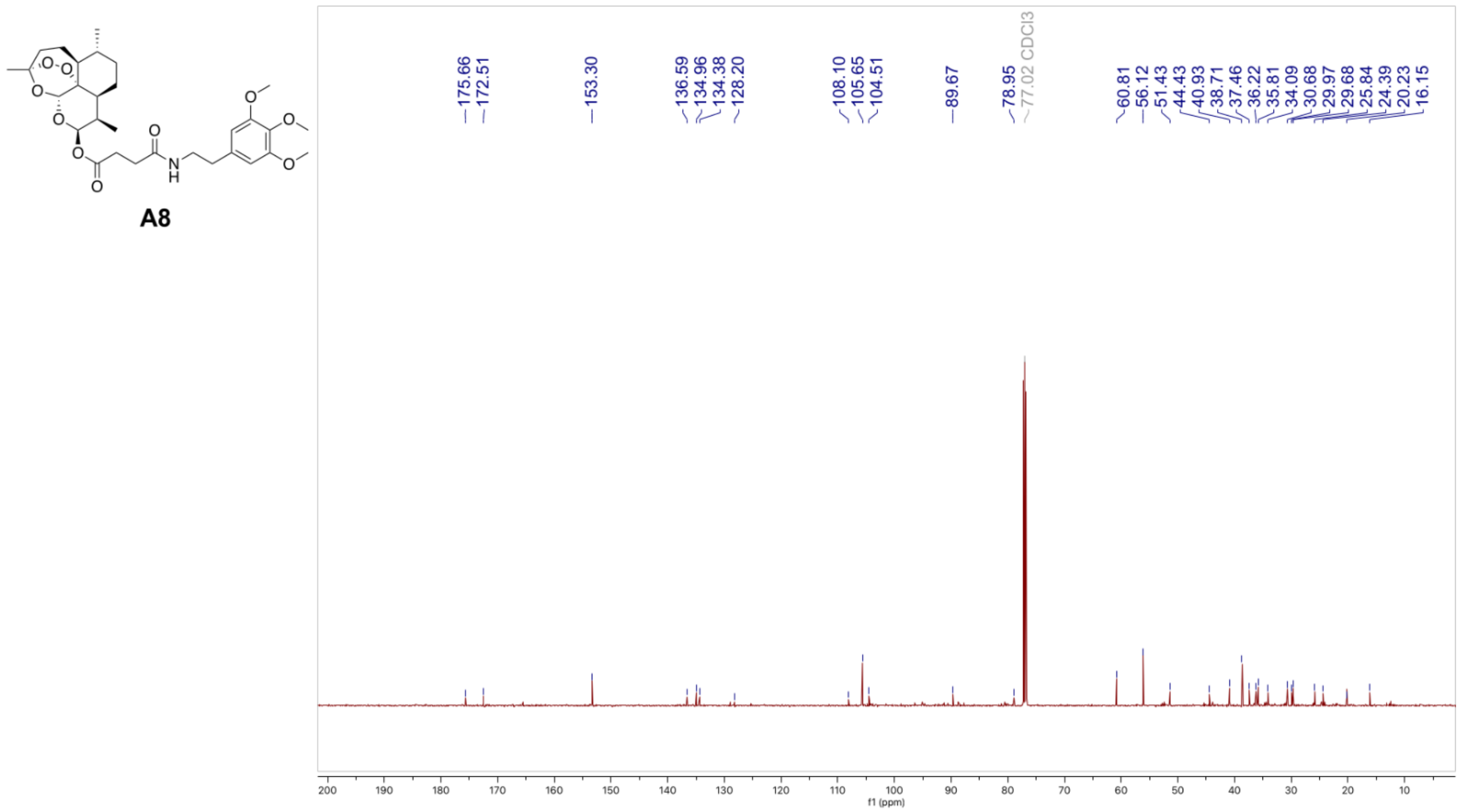

Fig. S16. ^{13}C NMR spectrum of compound **A8**.

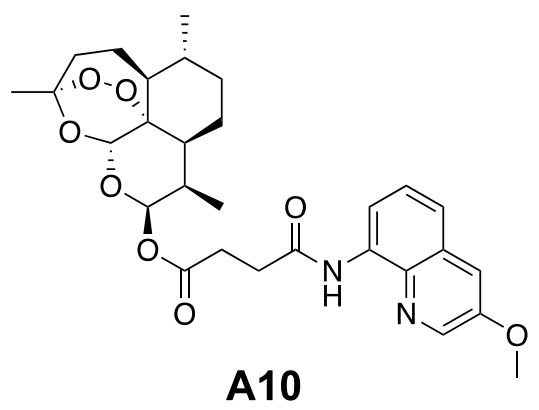

A10

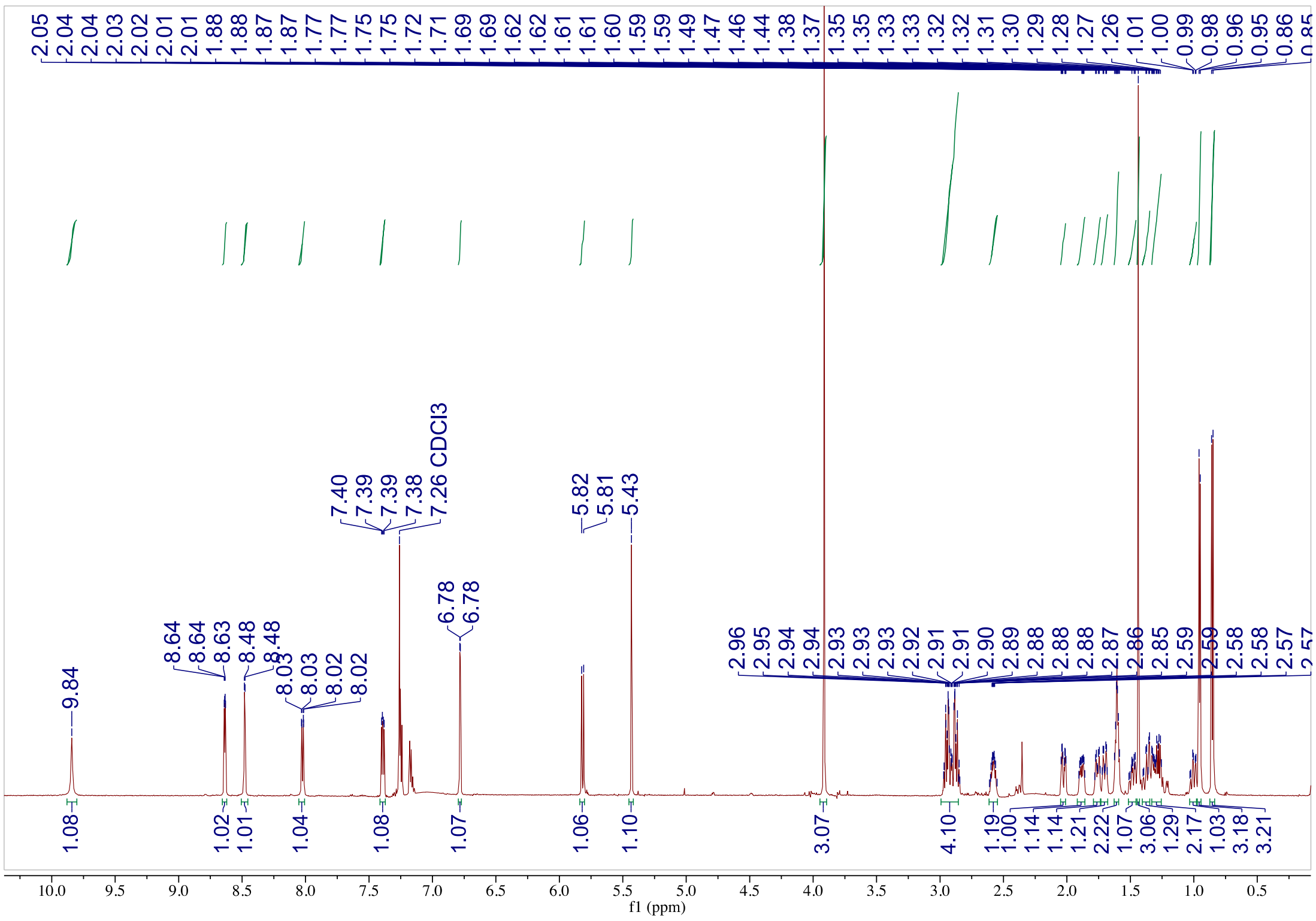

Fig. S17. ^{1}H NMR spectrum of compound **A10**.

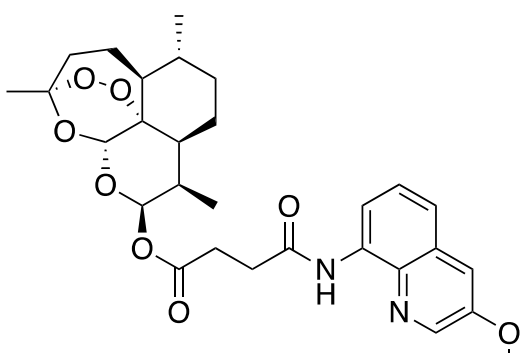

A10

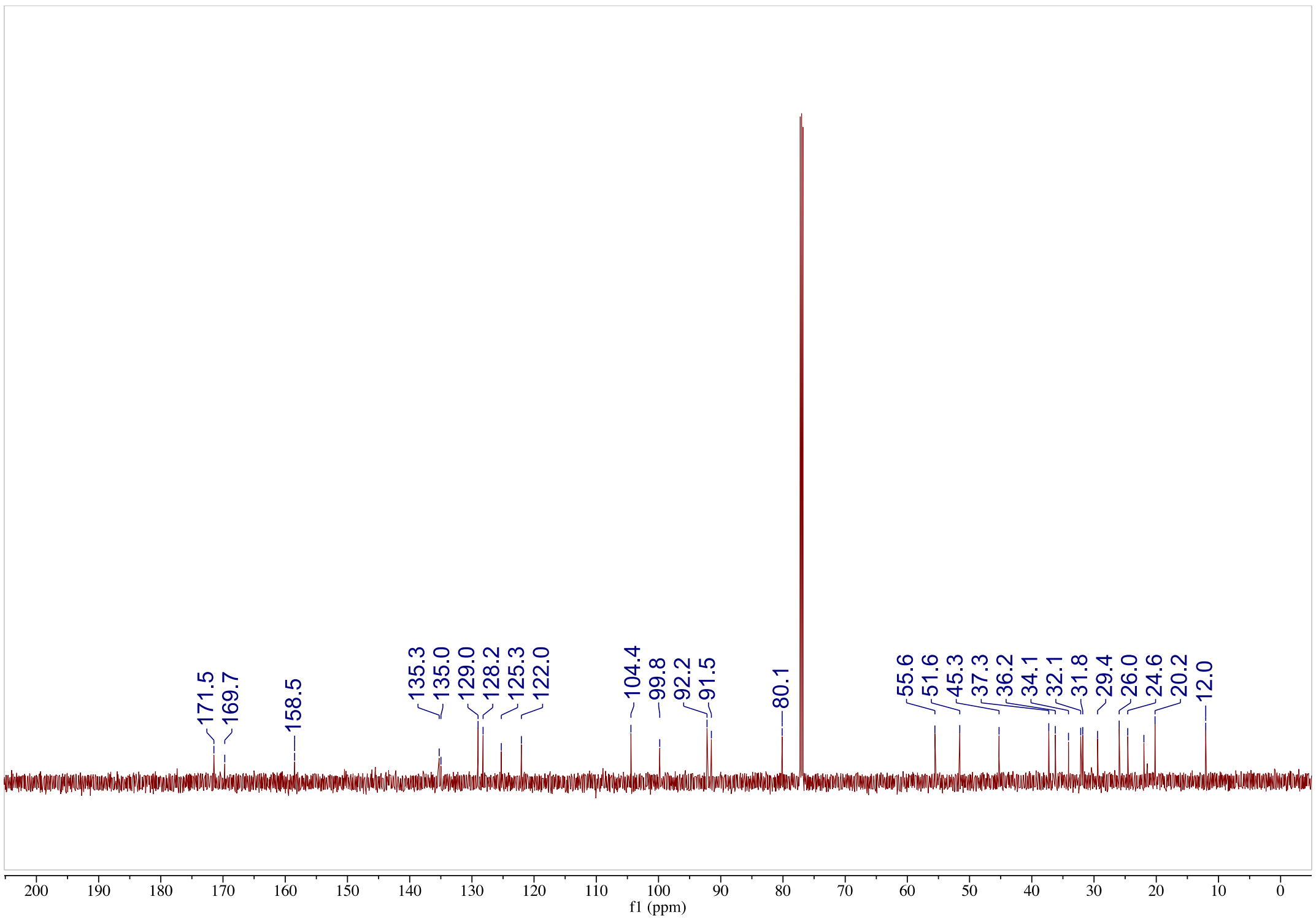

Fig. S18. ^{13}C NMR spectrum of compound **A10**.

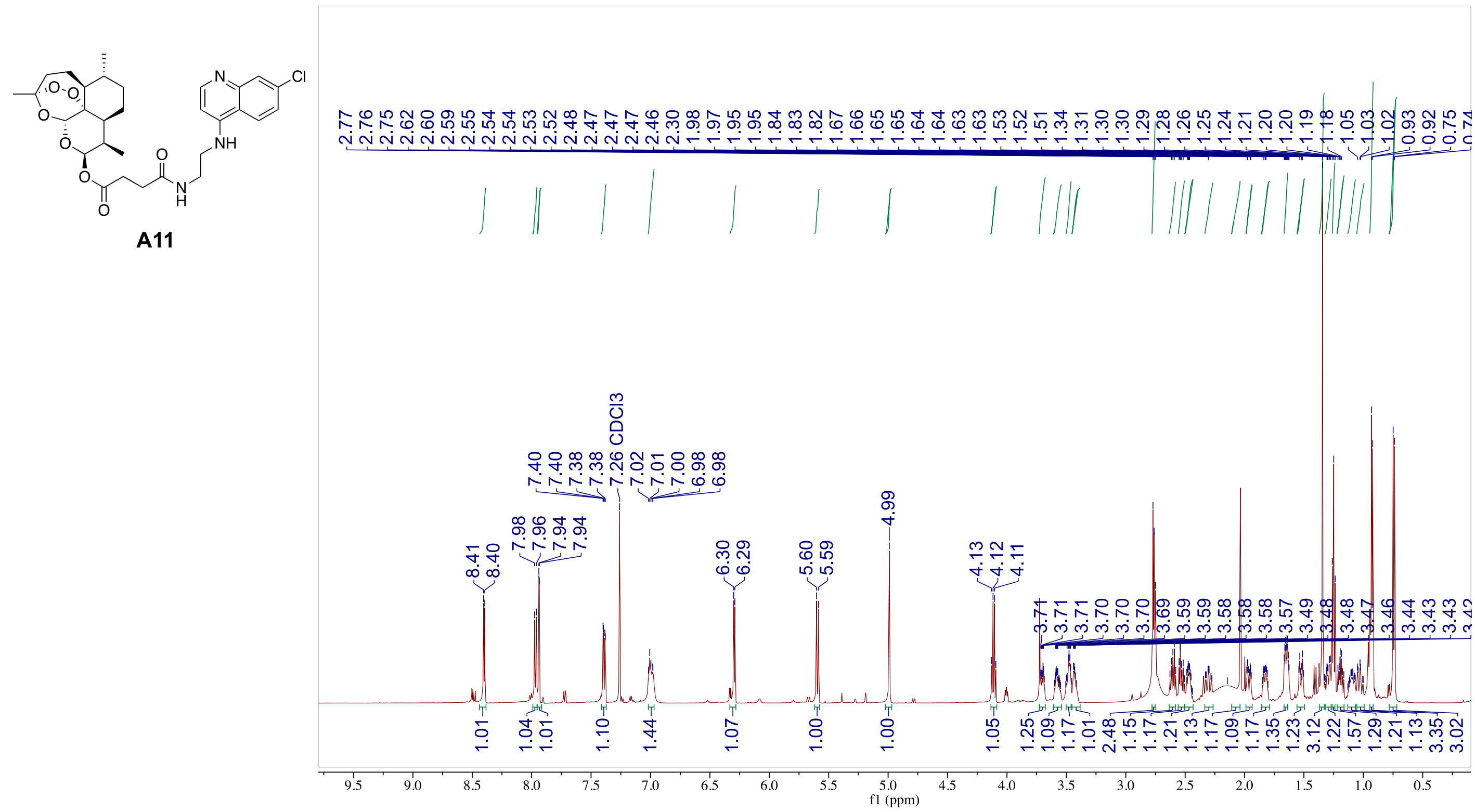

Fig. S19. ^{1}H NMR spectrum of compound **A11**.

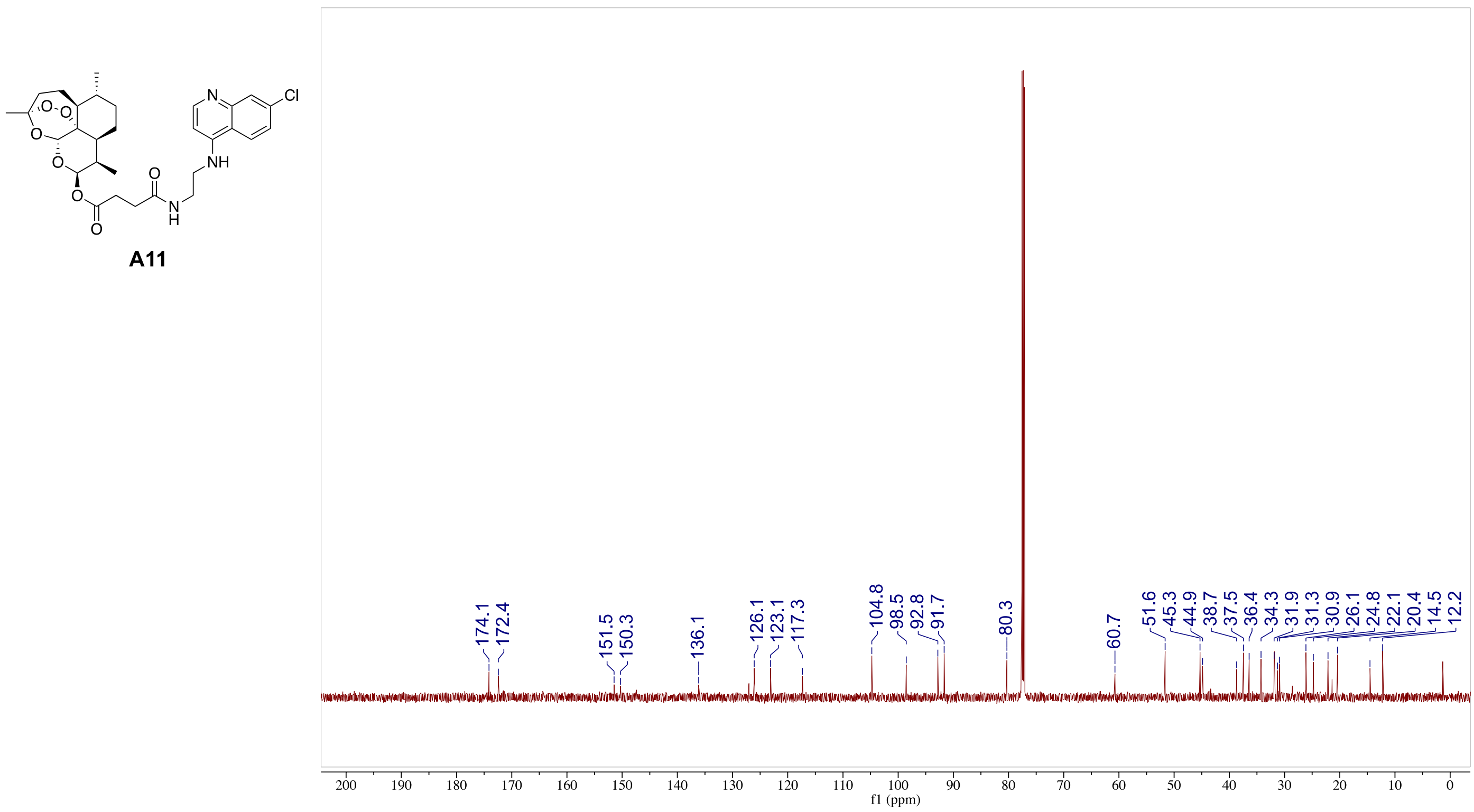

Fig. S20. ^{13}C NMR spectrum of compound **A11**.

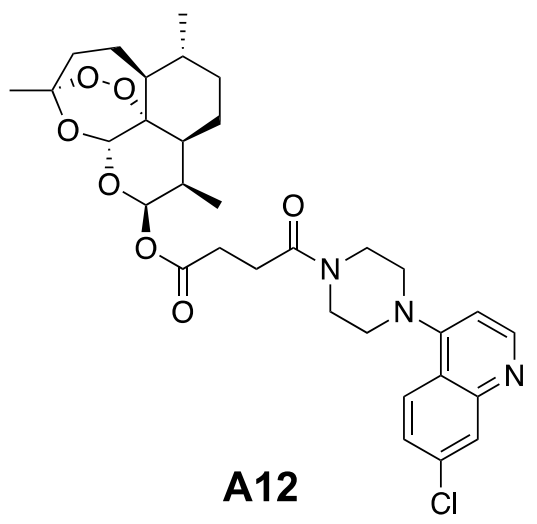

A12

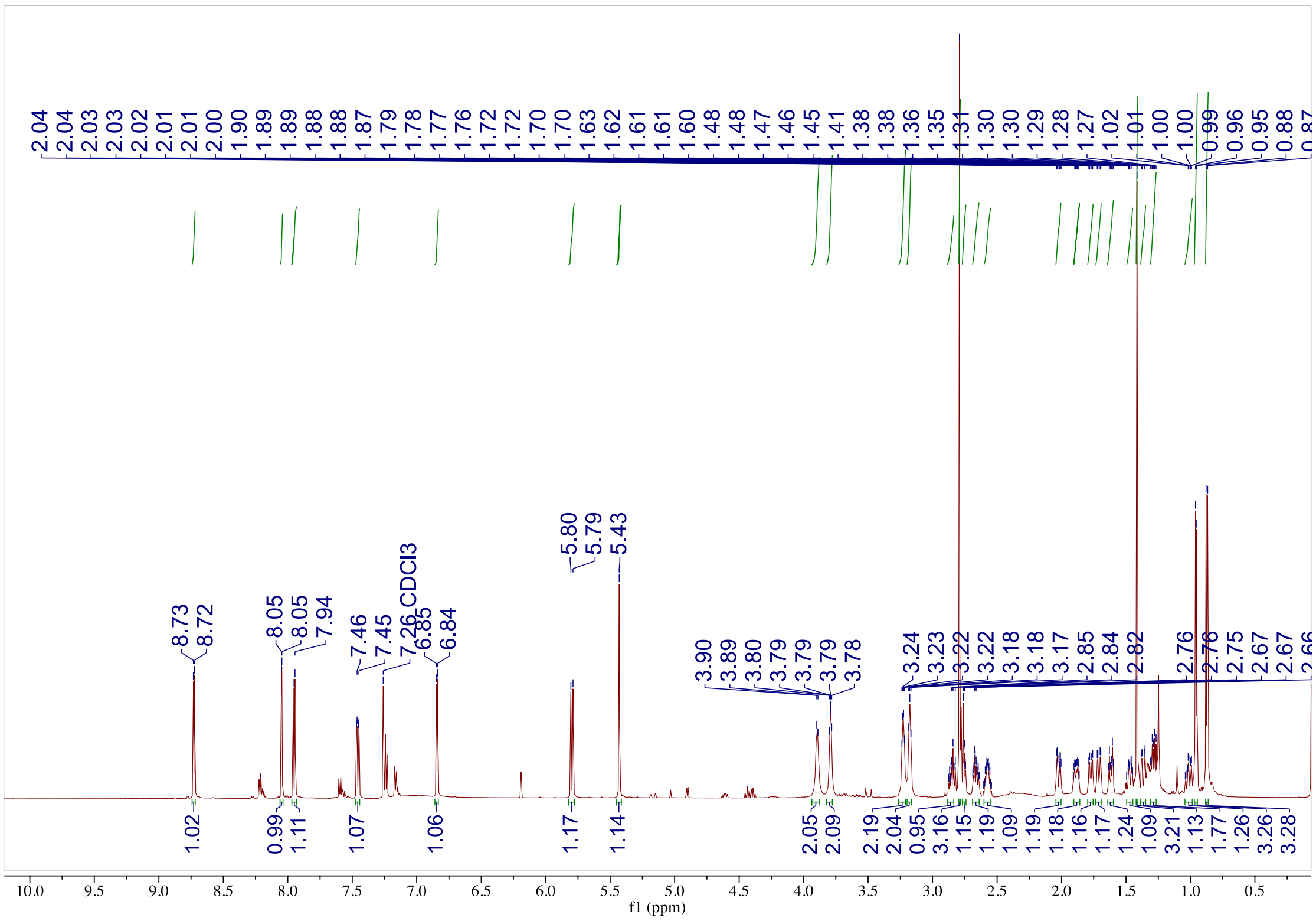

Fig. S21. ¹H NMR spectrum of compound **A12**.

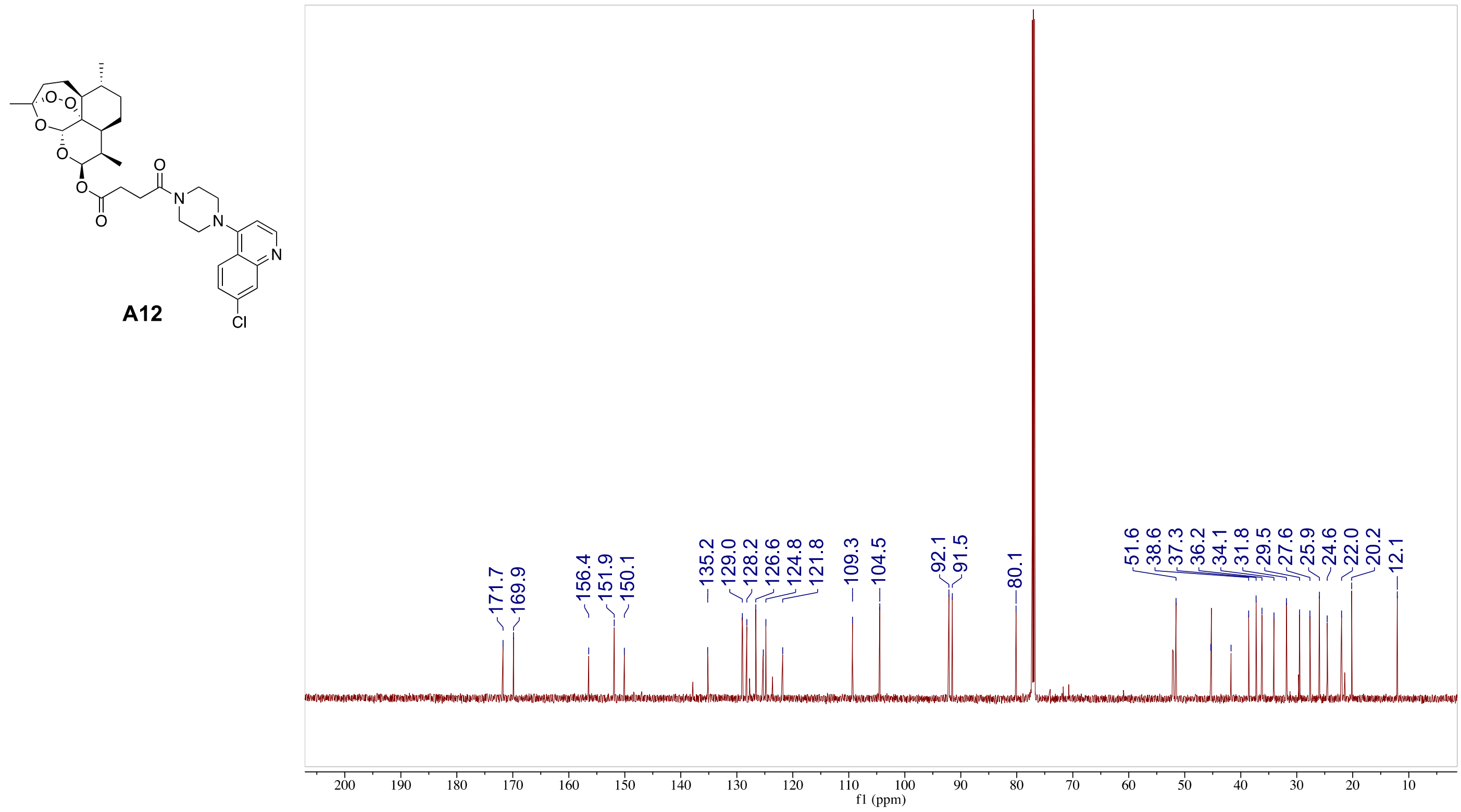

Fig. S22. ¹³C NMR spectrum of compound **A12**.

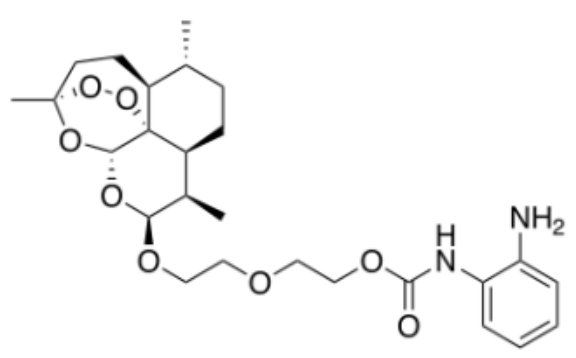

A13

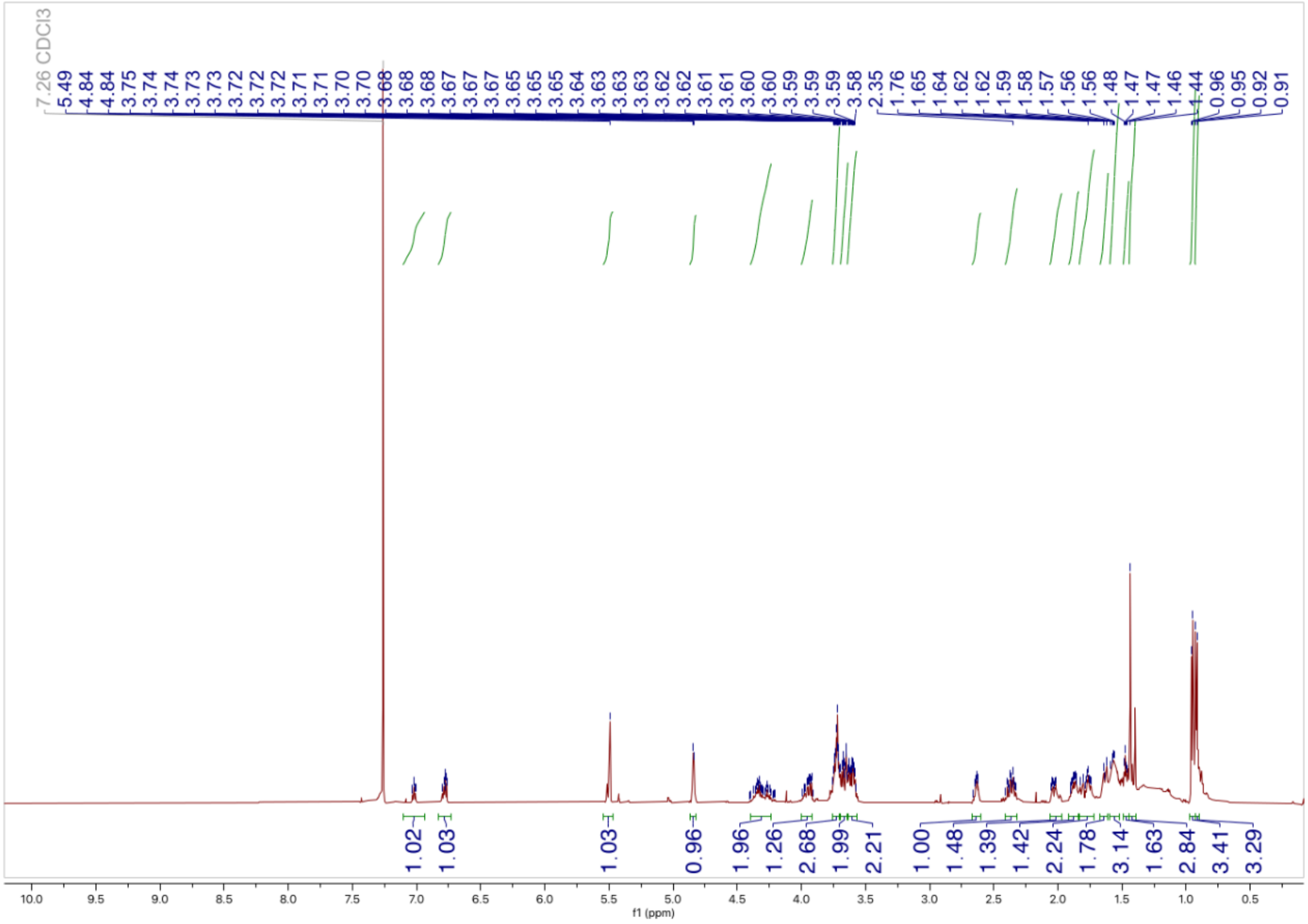

Fig. S23. ^{1}H NMR spectrum of compound **A13**.

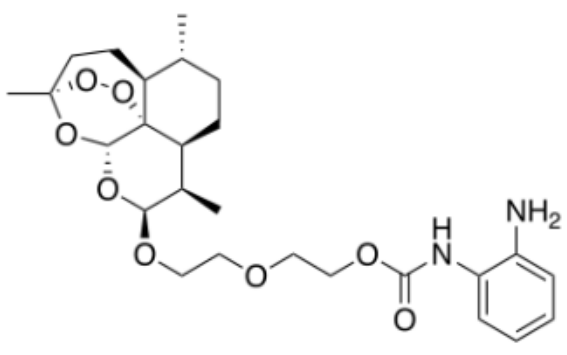

A13

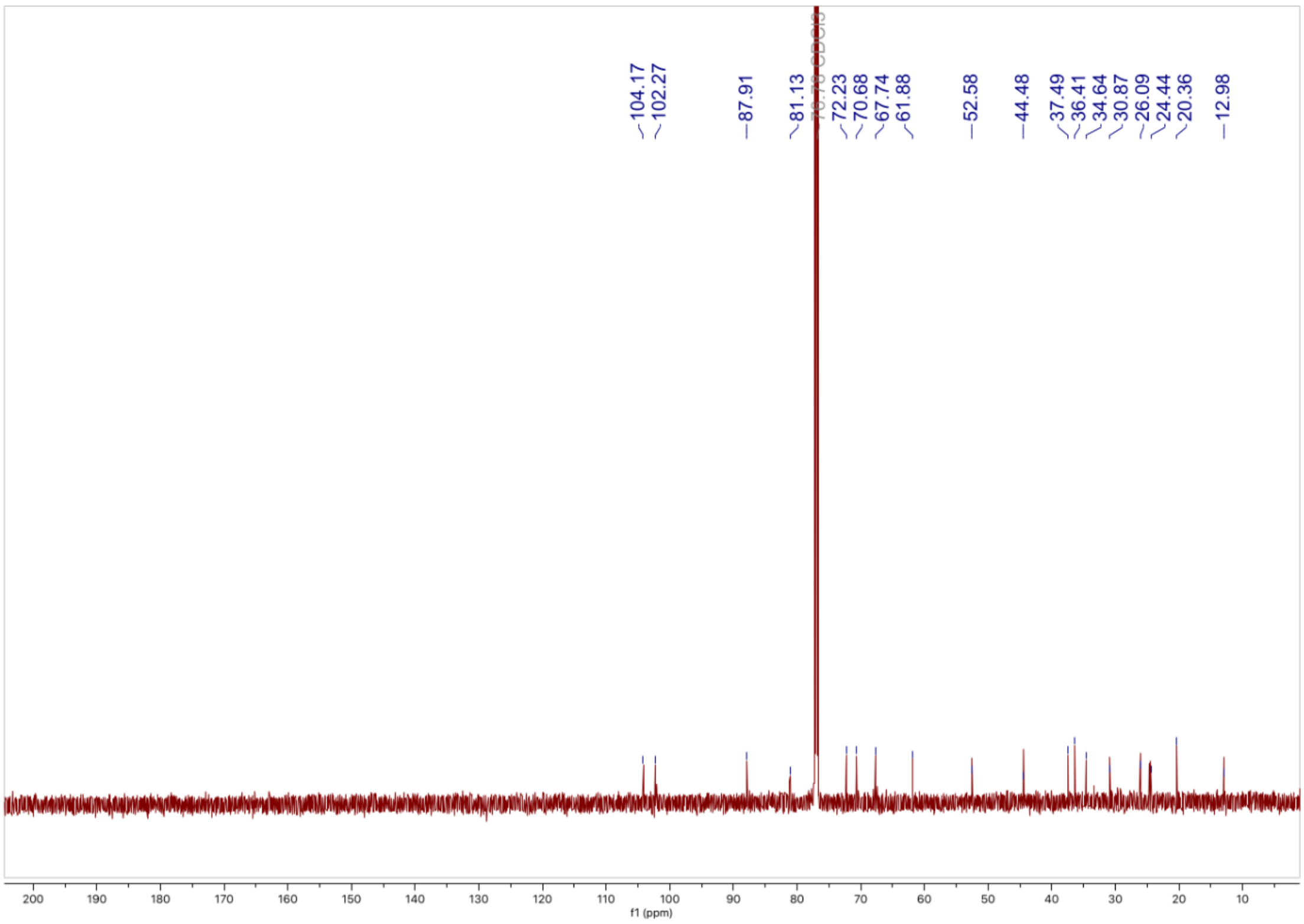

Fig. S24. ^{13}C NMR spectrum of compound **A13**.

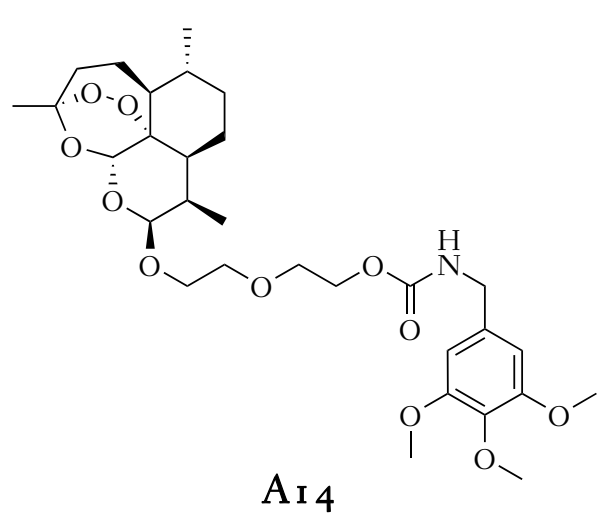

A14

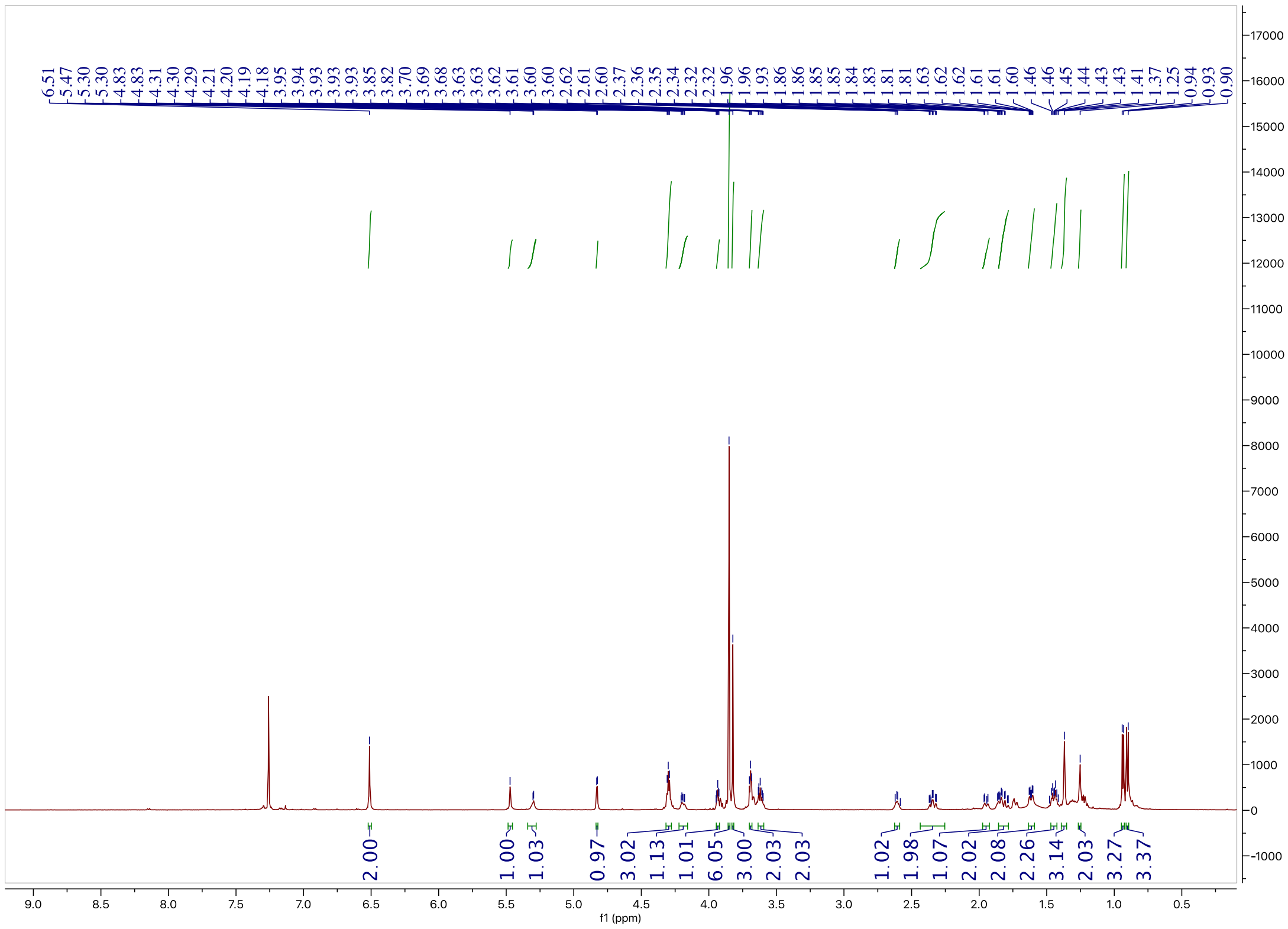

Fig. S25. ^{1}H NMR spectrum of compound **A14**.

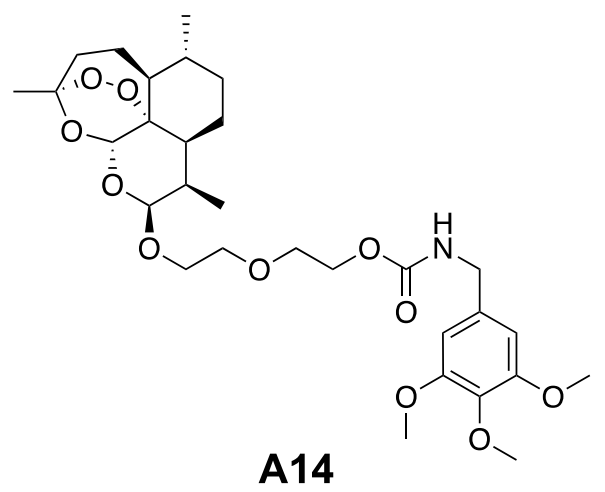

A14

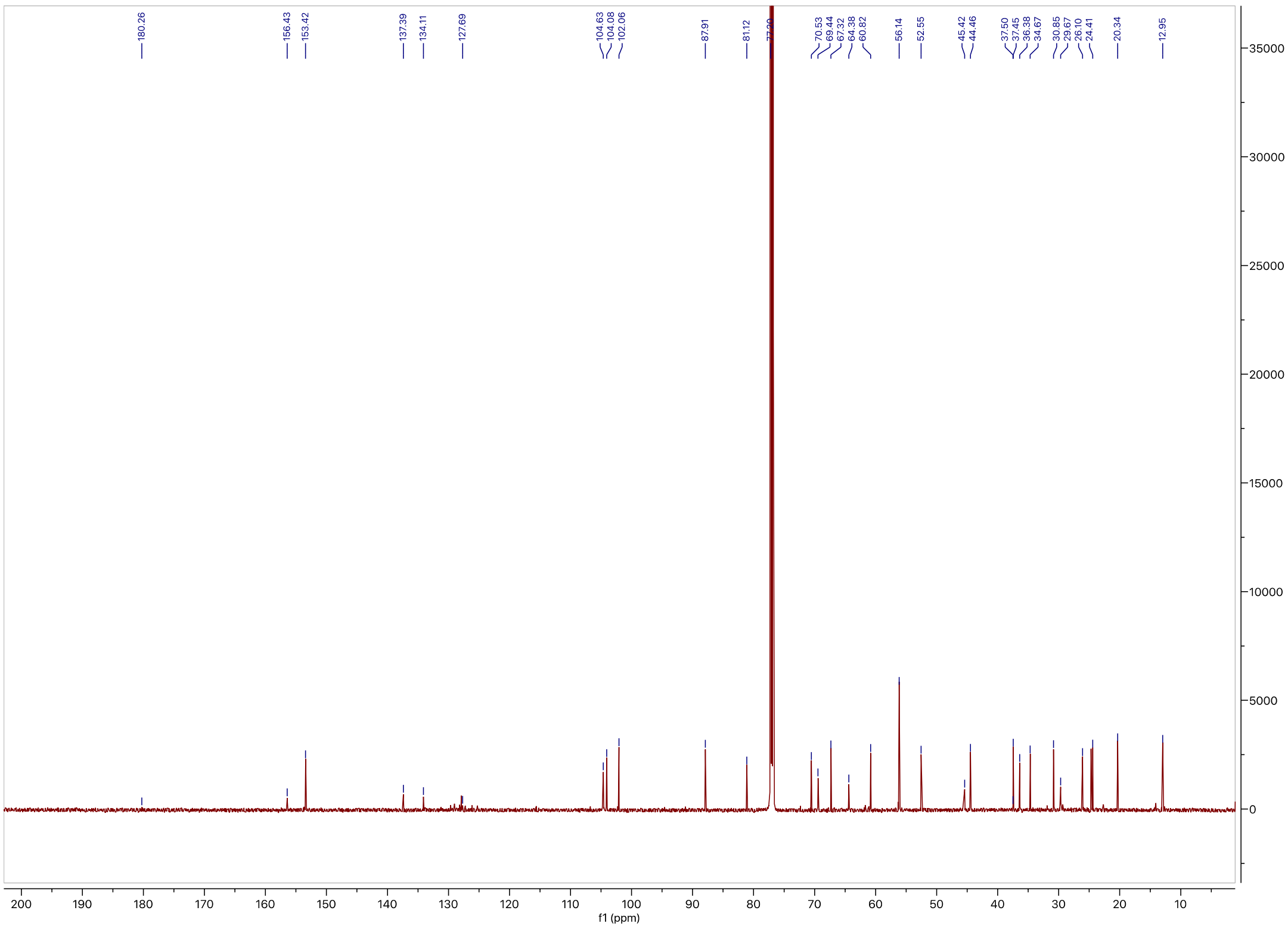

Fig. S26. ^{13}C NMR spectrum of compound **A14**.

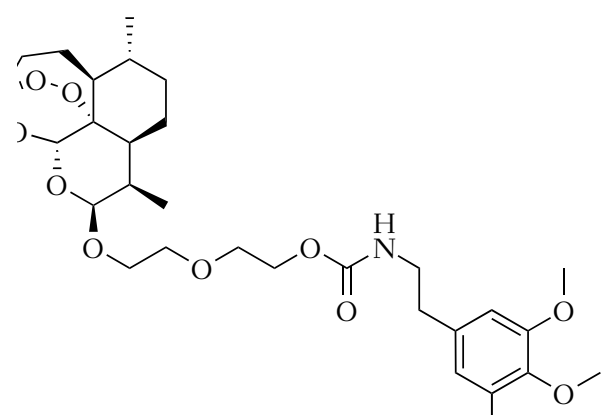

A15

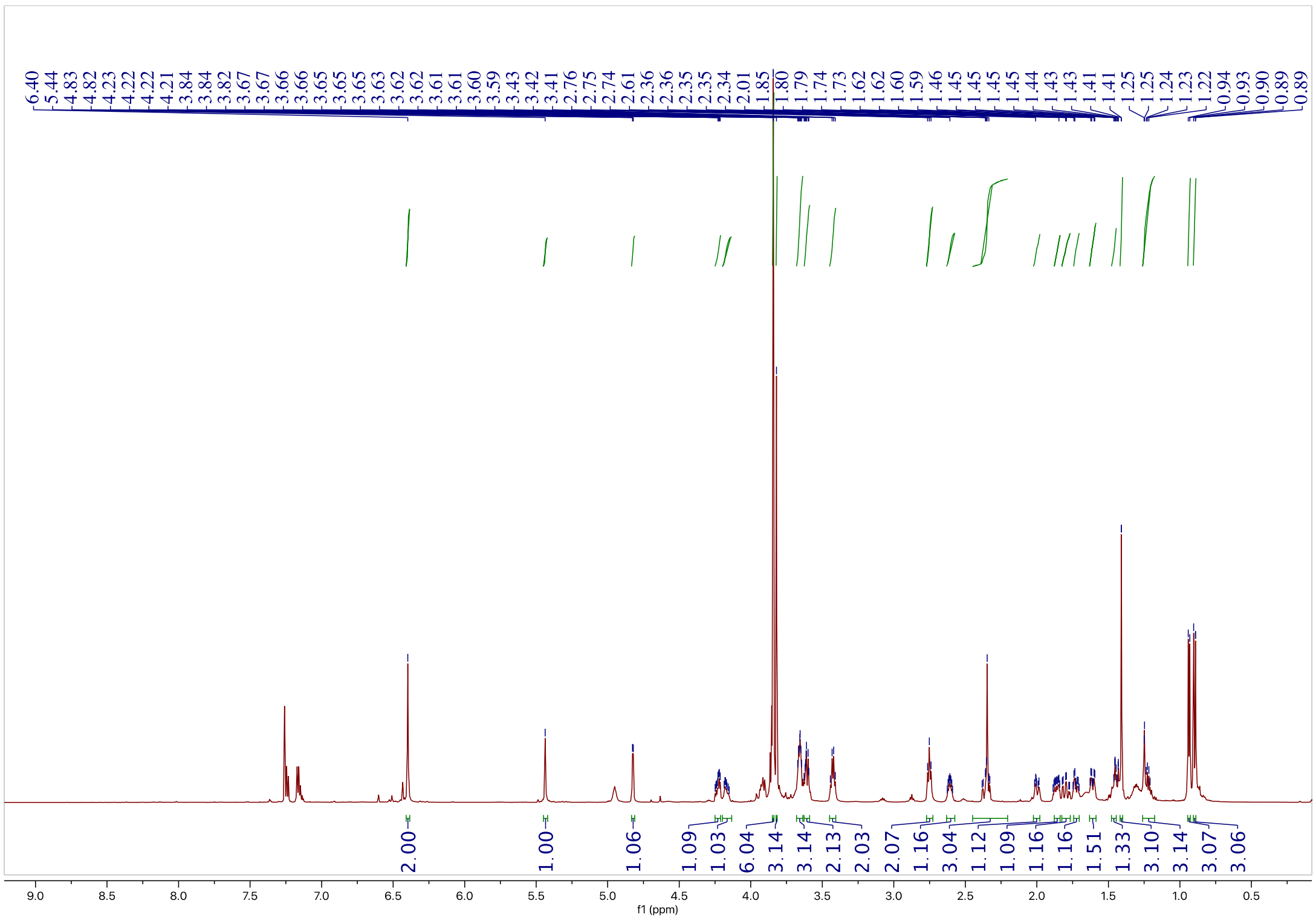

Fig. S27. ^{1}H NMR spectrum of compound **A15**.

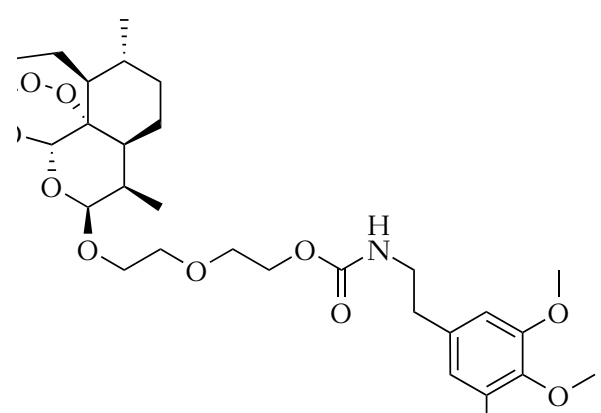

A15

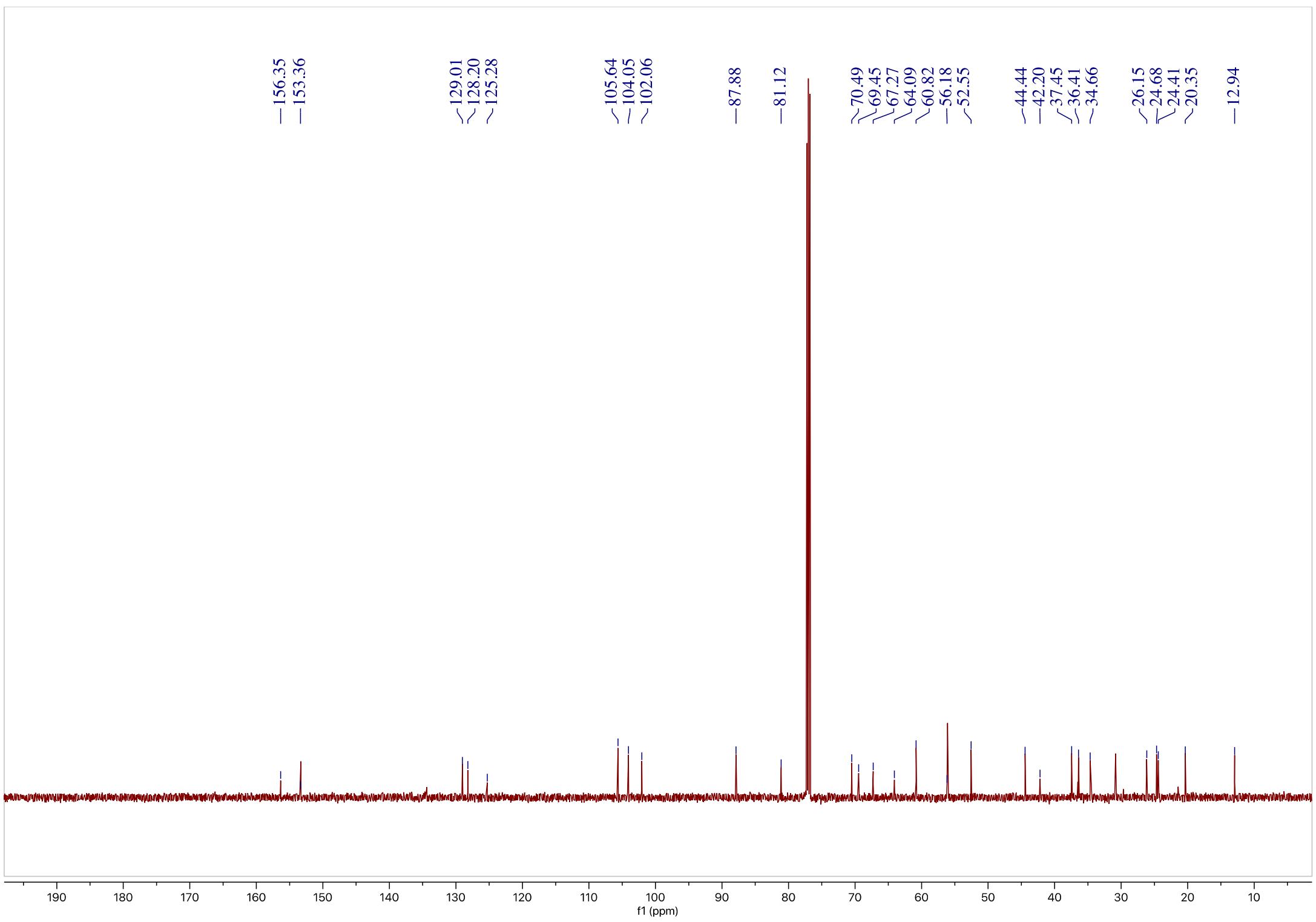

Fig. S28. ^{13}C NMR spectrum of compound **A15**.

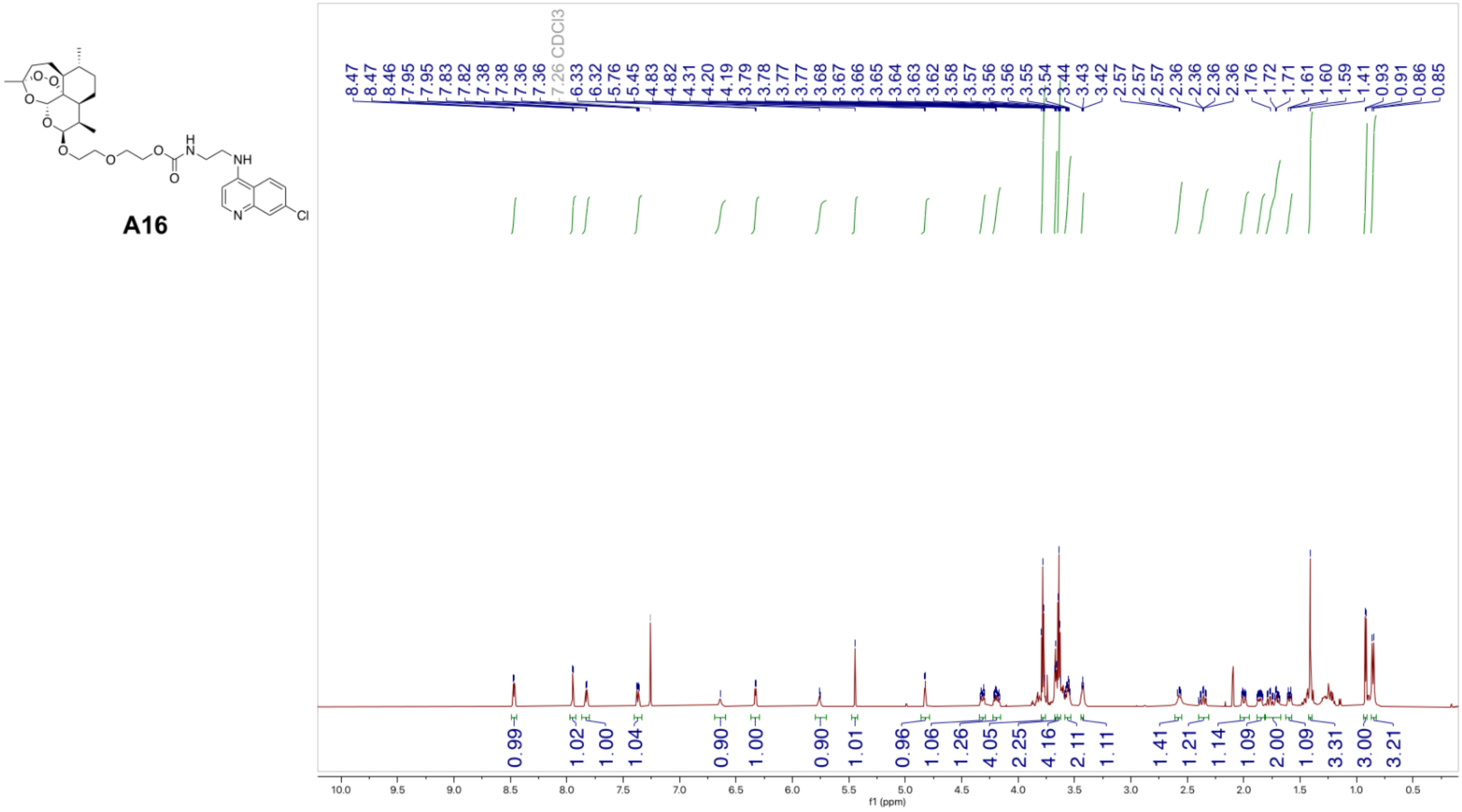

Fig. S29. ¹H NMR spectrum of compound **A16**.

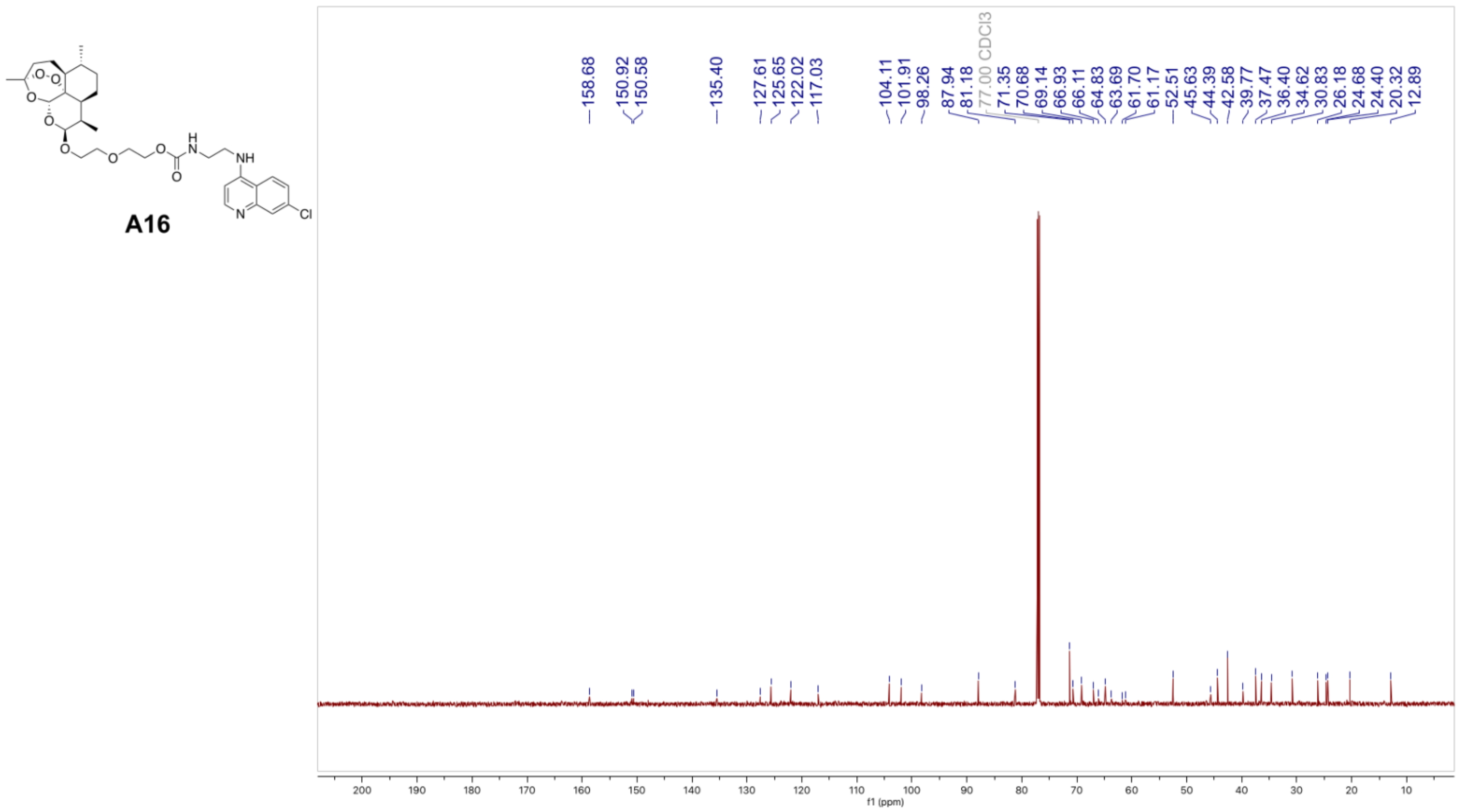

Fig. S30. ¹³C NMR spectrum of compound **A16**.

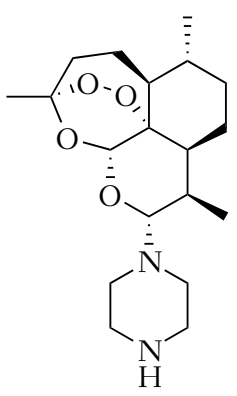

A17

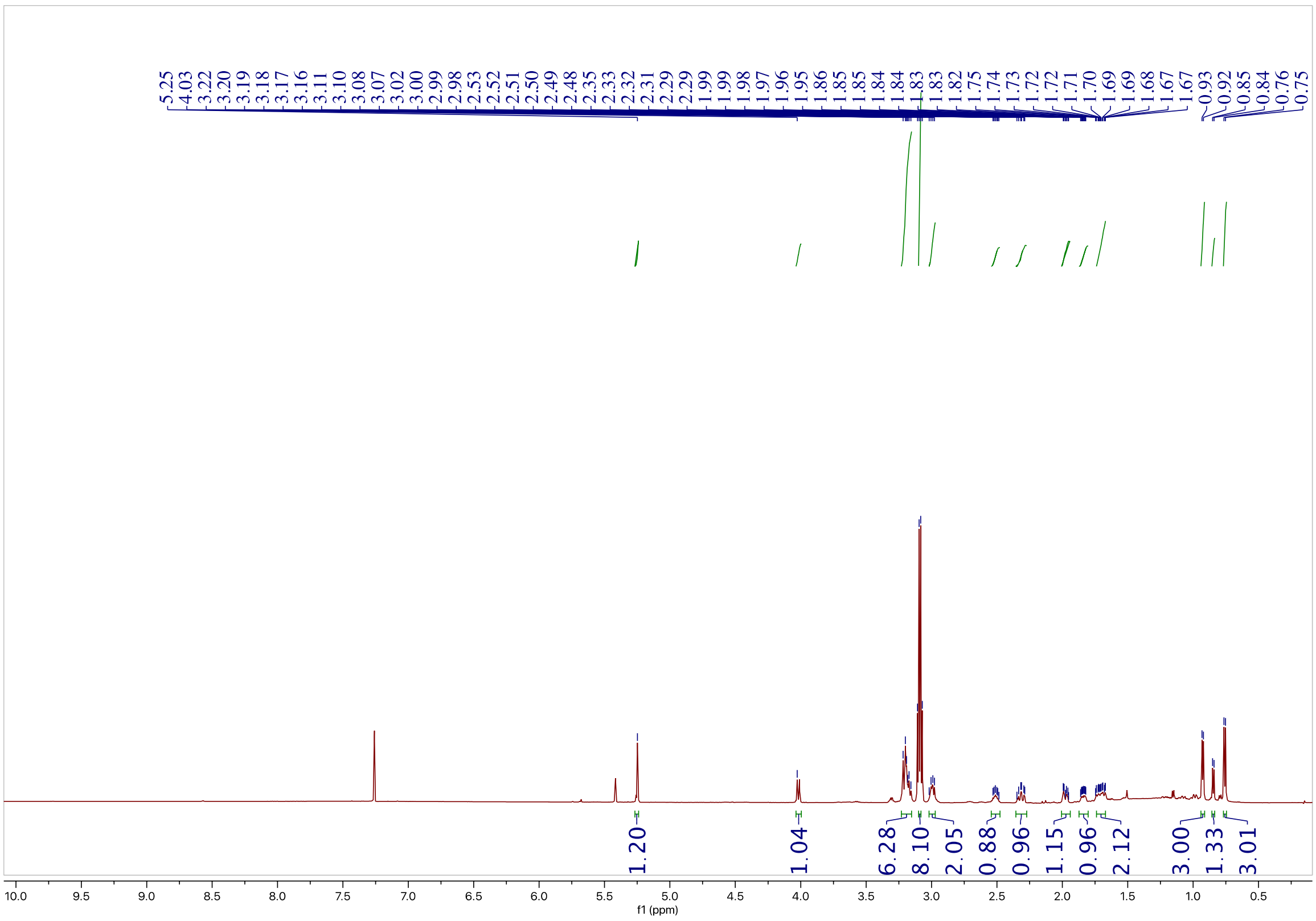

Fig. S31. ^{1}H NMR spectrum of compound **A17**.

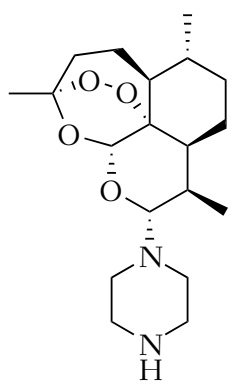

A17

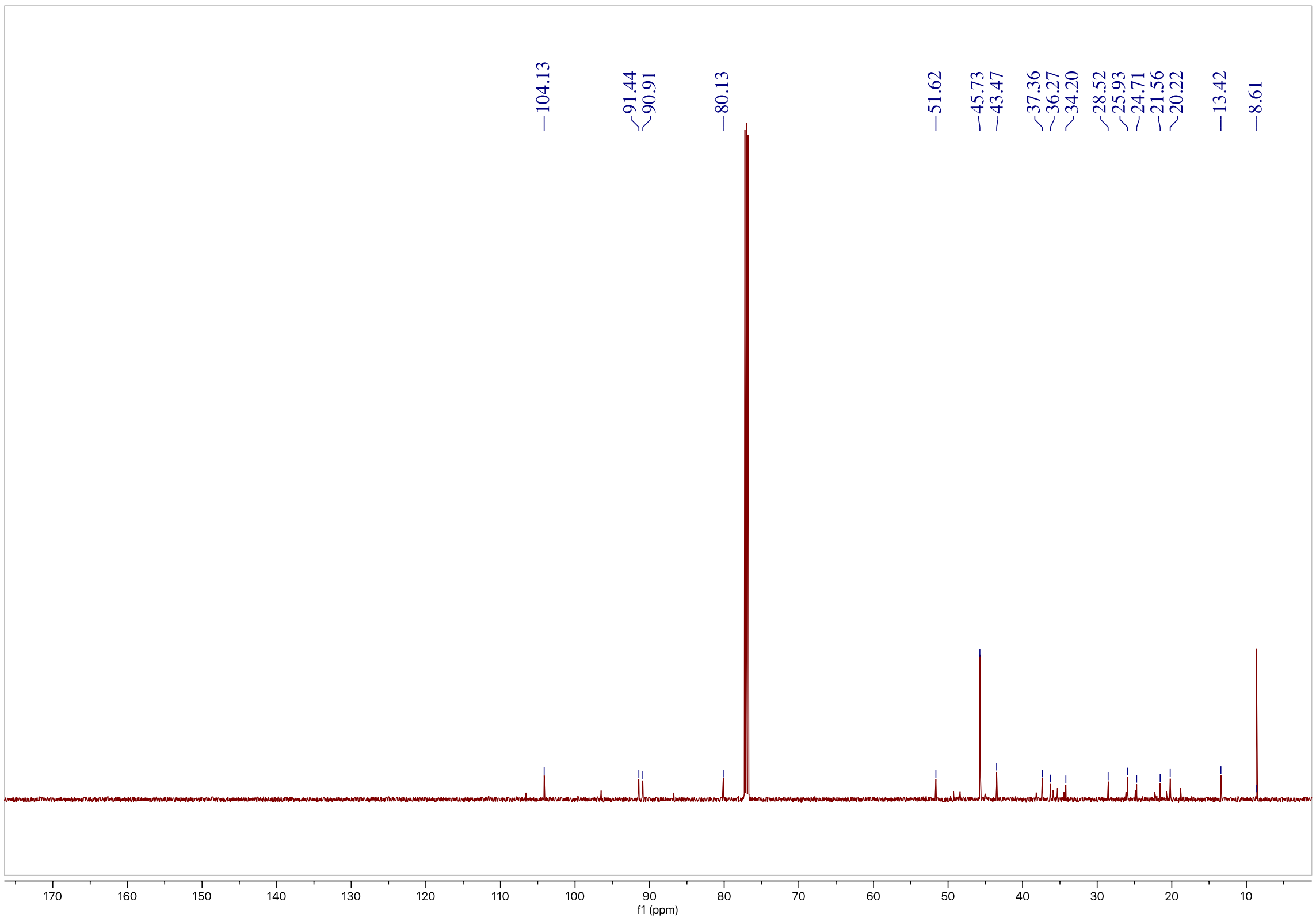

Fig. S32. ^{13}C NMR spectrum of compound **A17**.

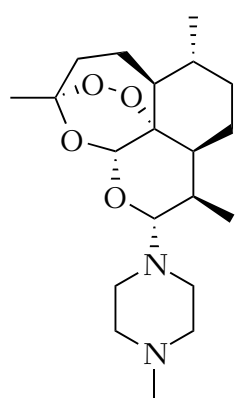

A18

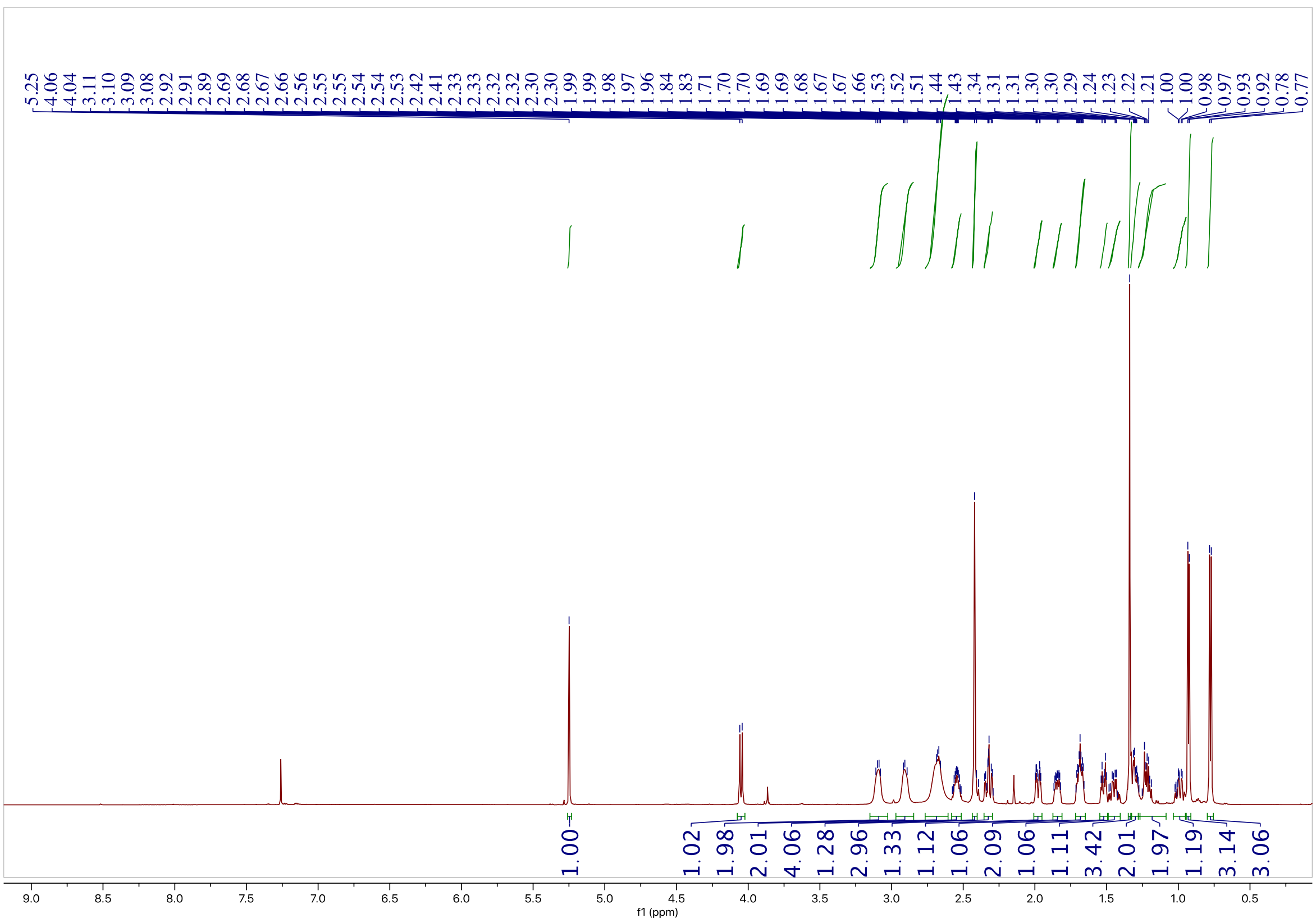

Fig. S33. ^{1}H NMR spectrum of compound **A18**.

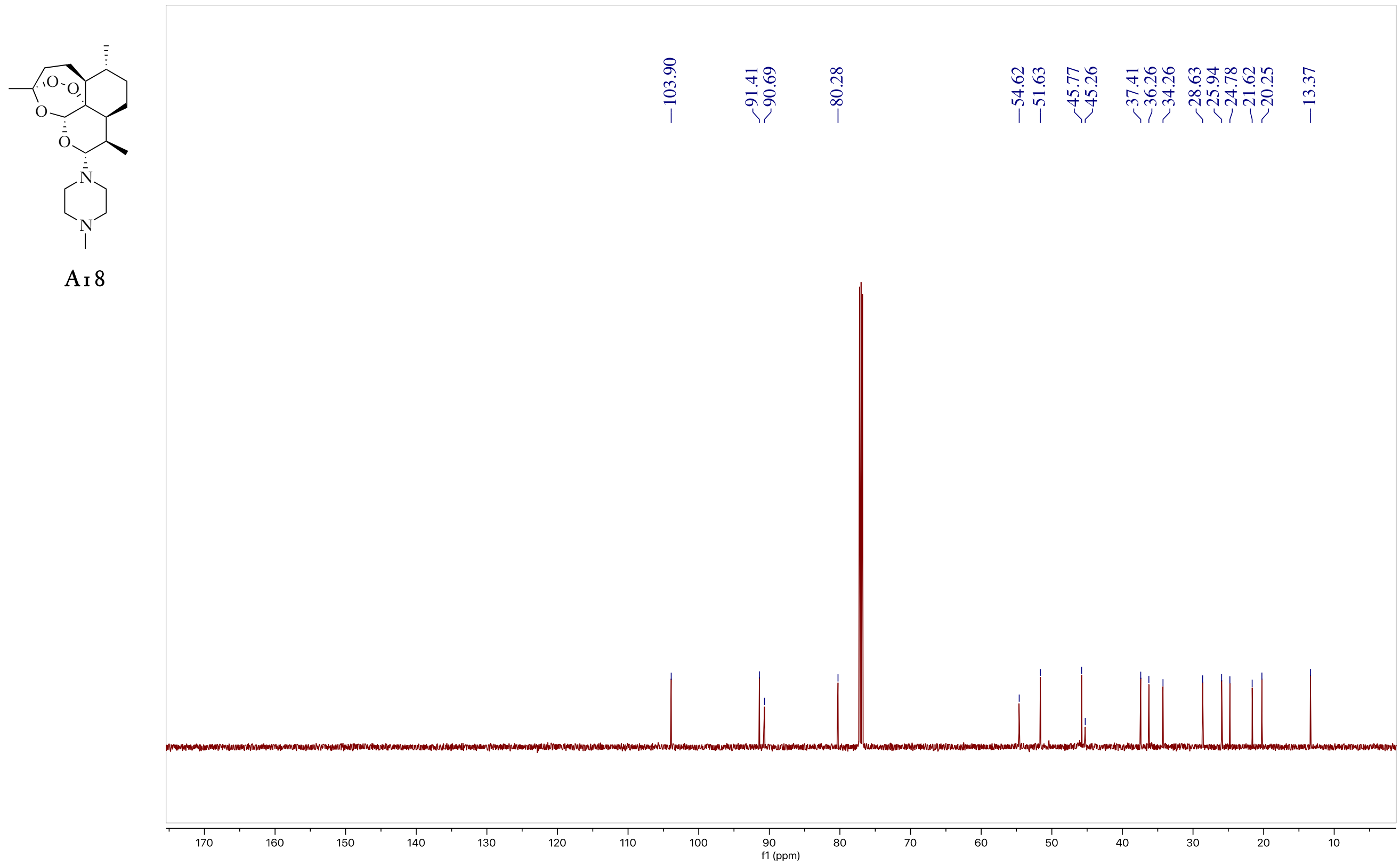

Fig. S34. ^{13}C NMR spectrum of compound **A18**.

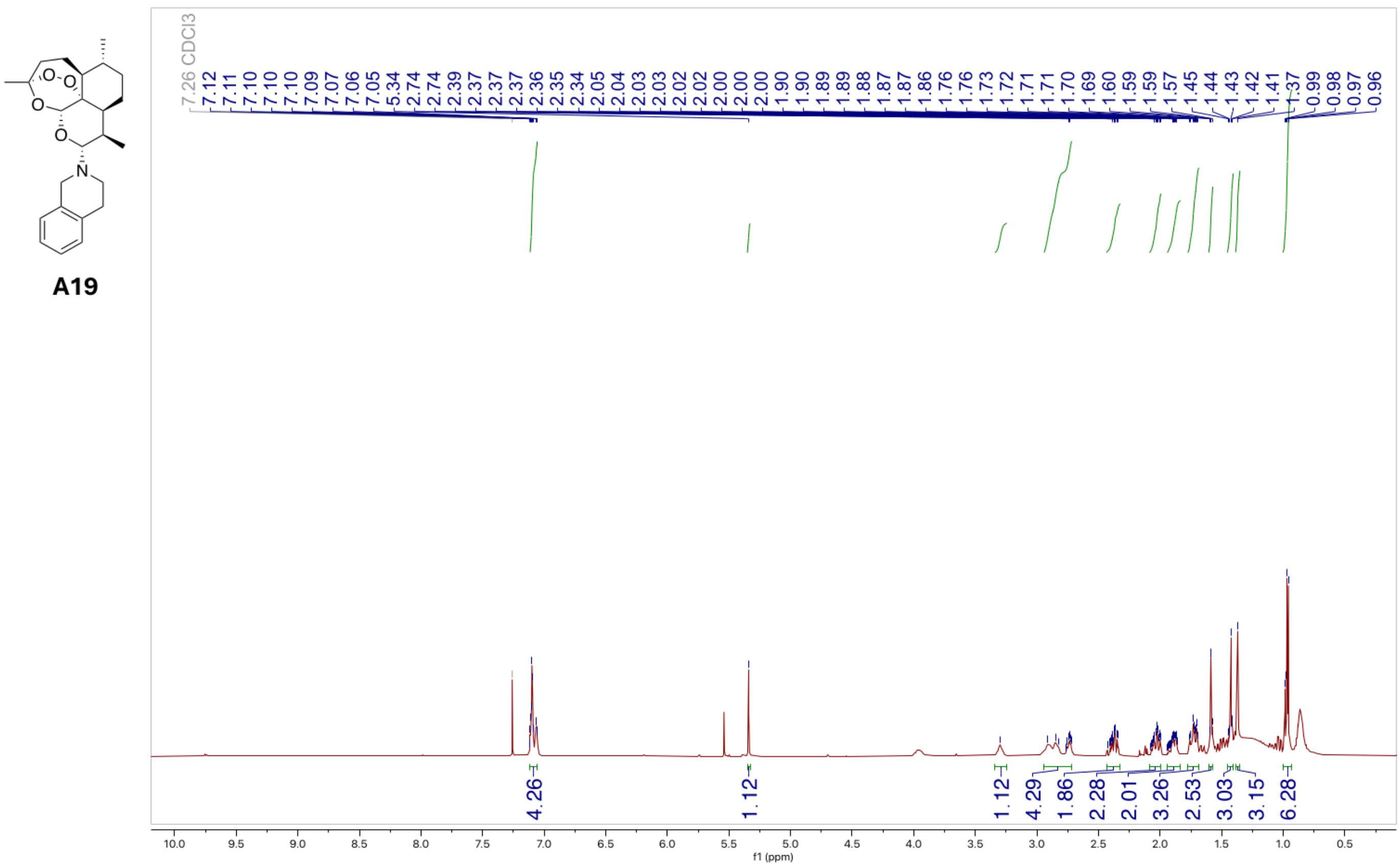

Fig. S35. ^{1}H NMR spectrum of compound **A19**.

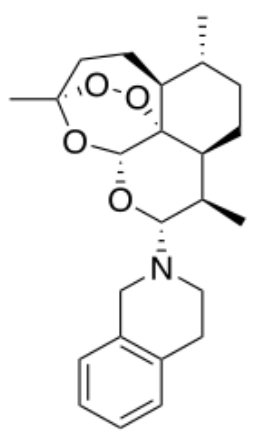

A19

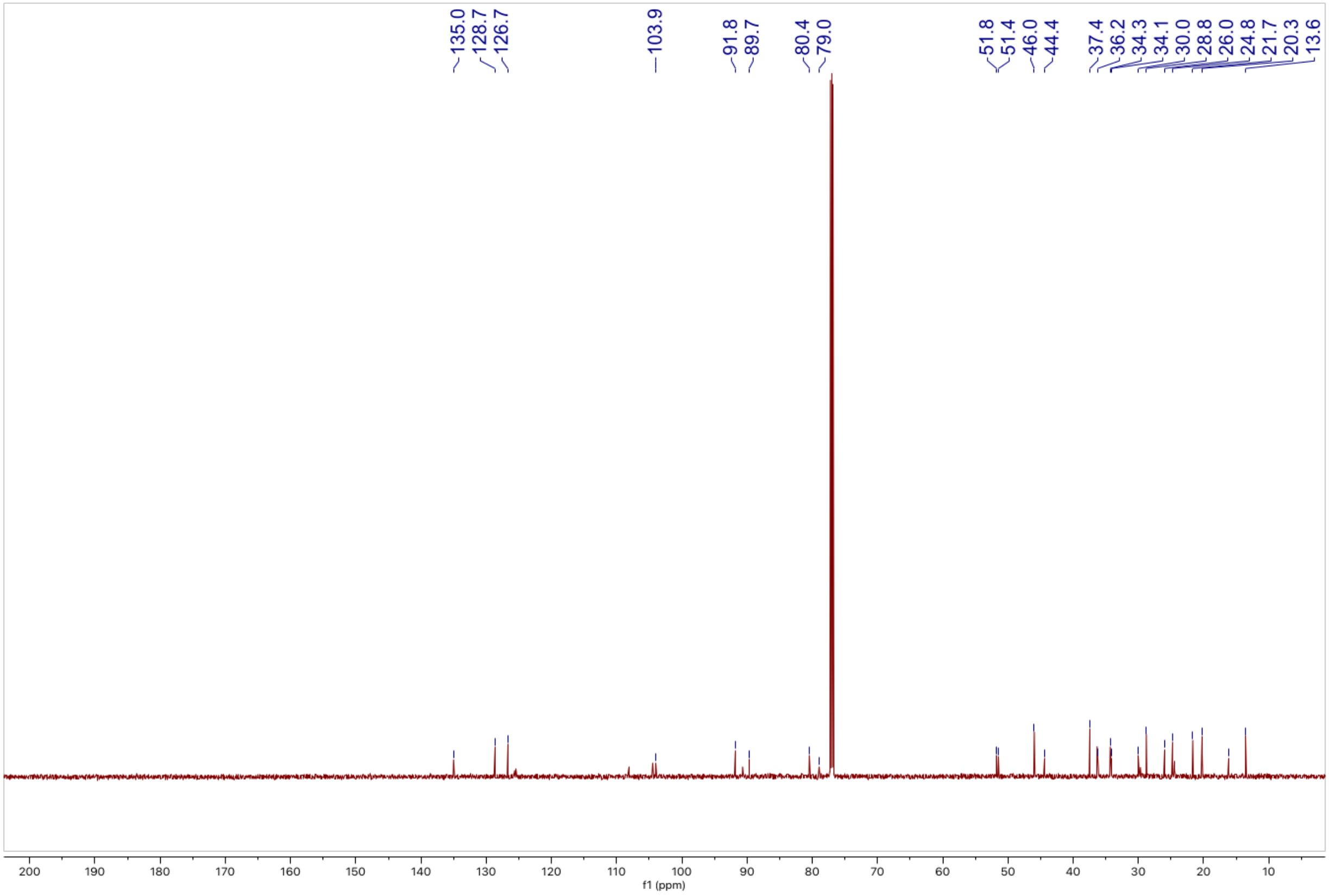

Fig. S36. ^{13}C NMR spectrum of compound **A19**.

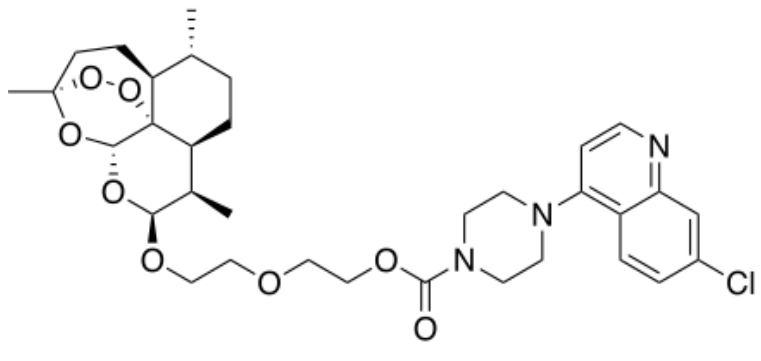

A20

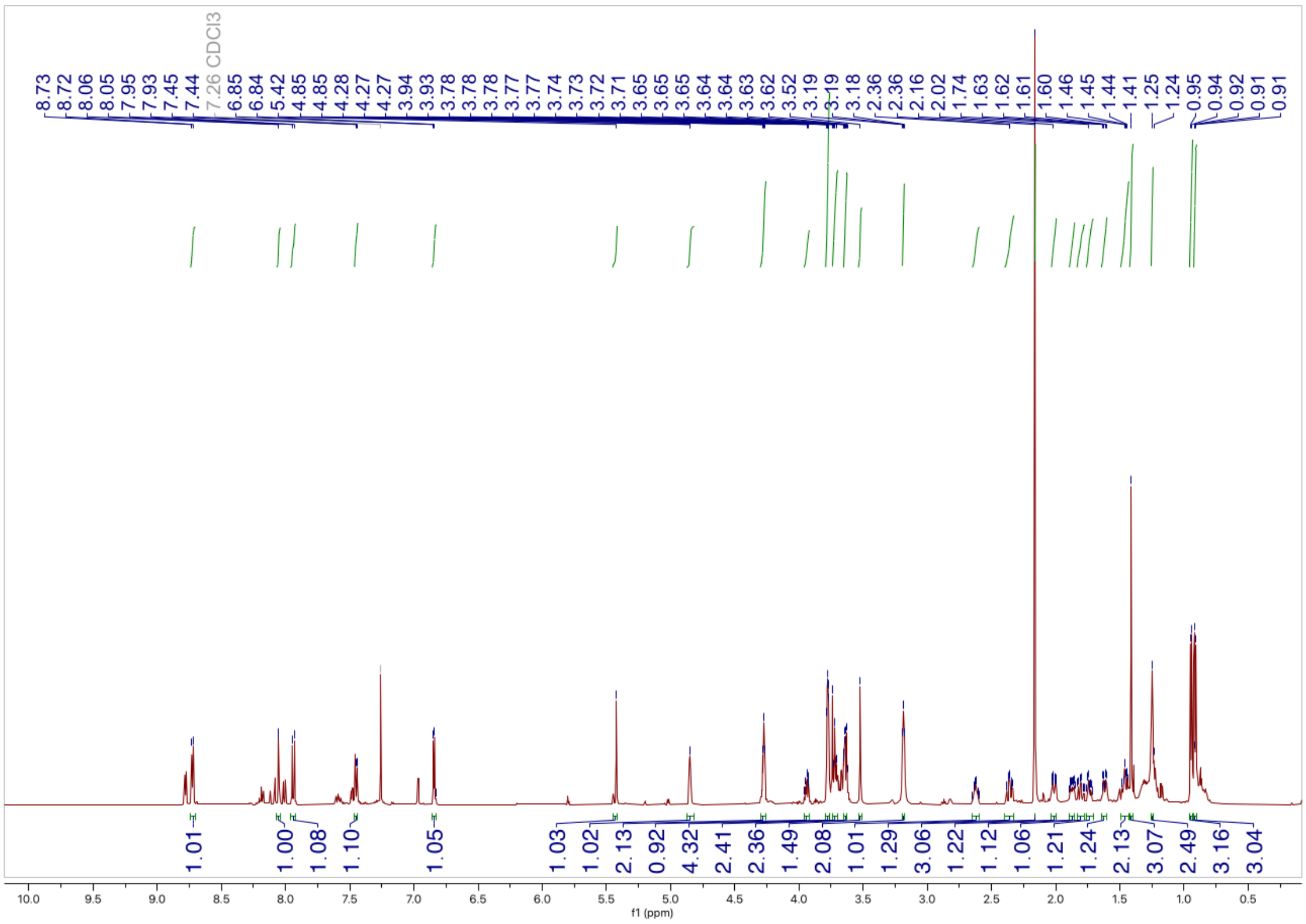

Fig. S37. ^{1}H NMR spectrum of compound **A20.**

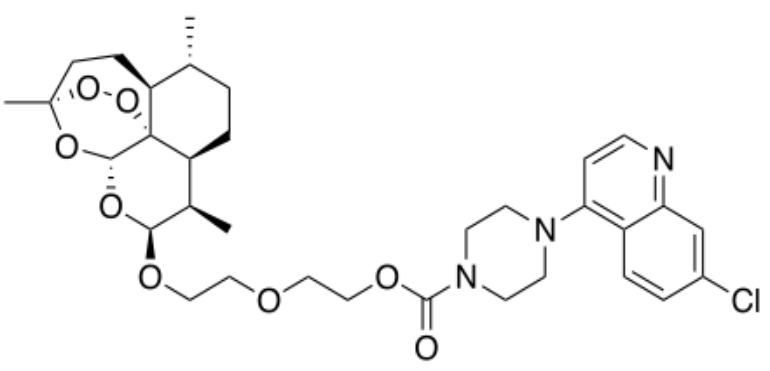

A20

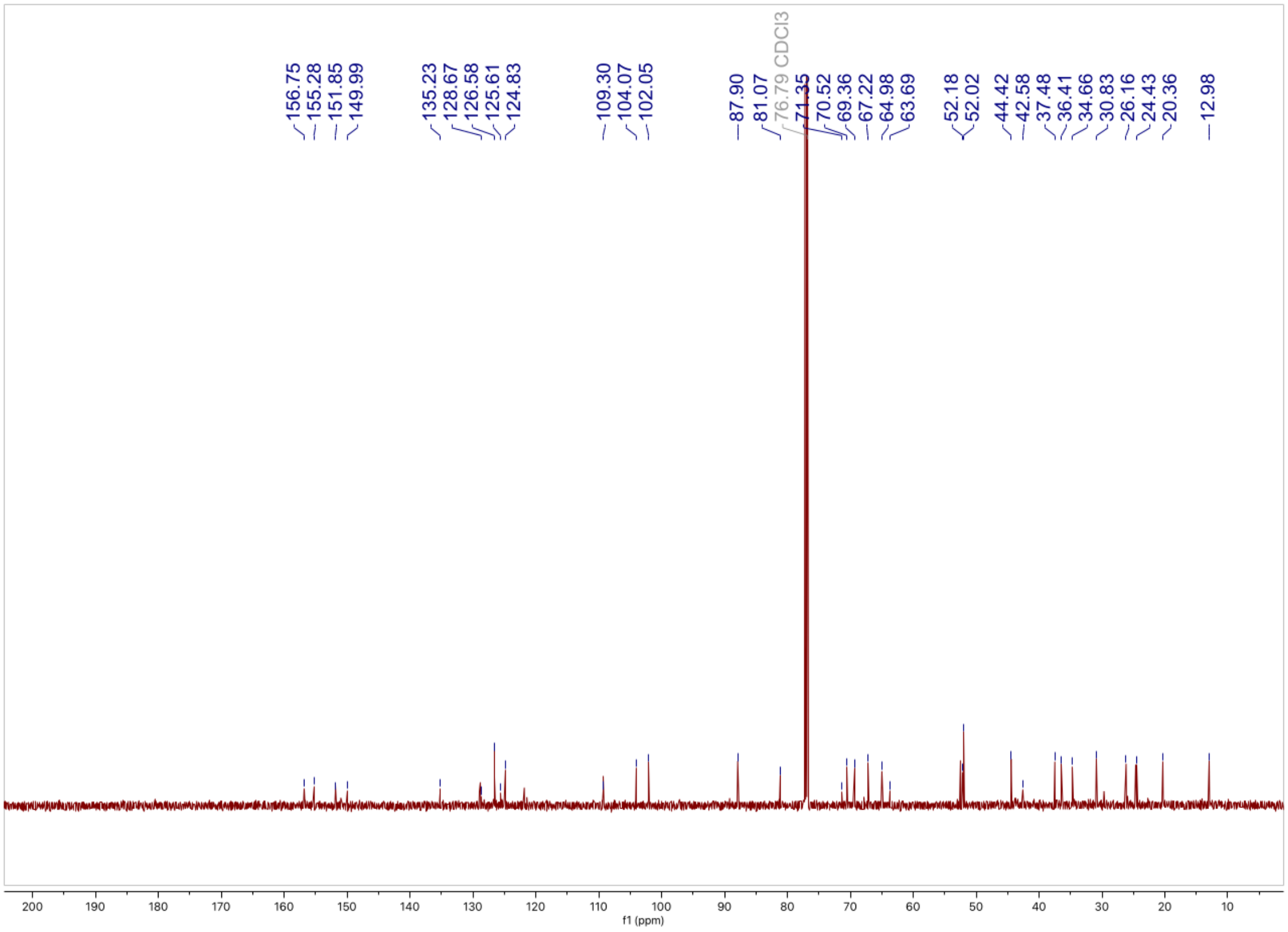

Fig. S38. ^{13}C NMR spectrum of compound **A20.**

Experimental HPLC chromatograms

A1

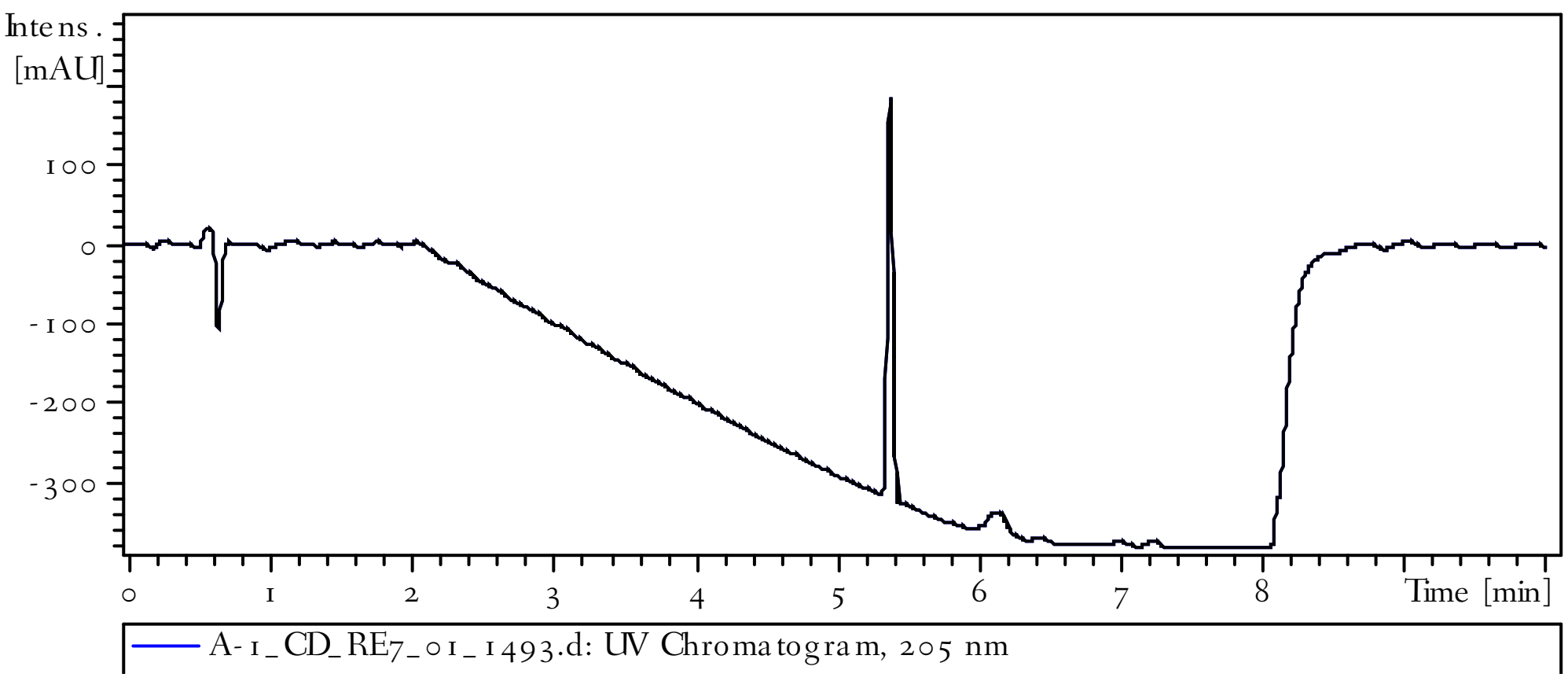

t_R:5.4 min, 95.5%

Fig. S39. HPLC chromatogram of compound **A1**.

A2

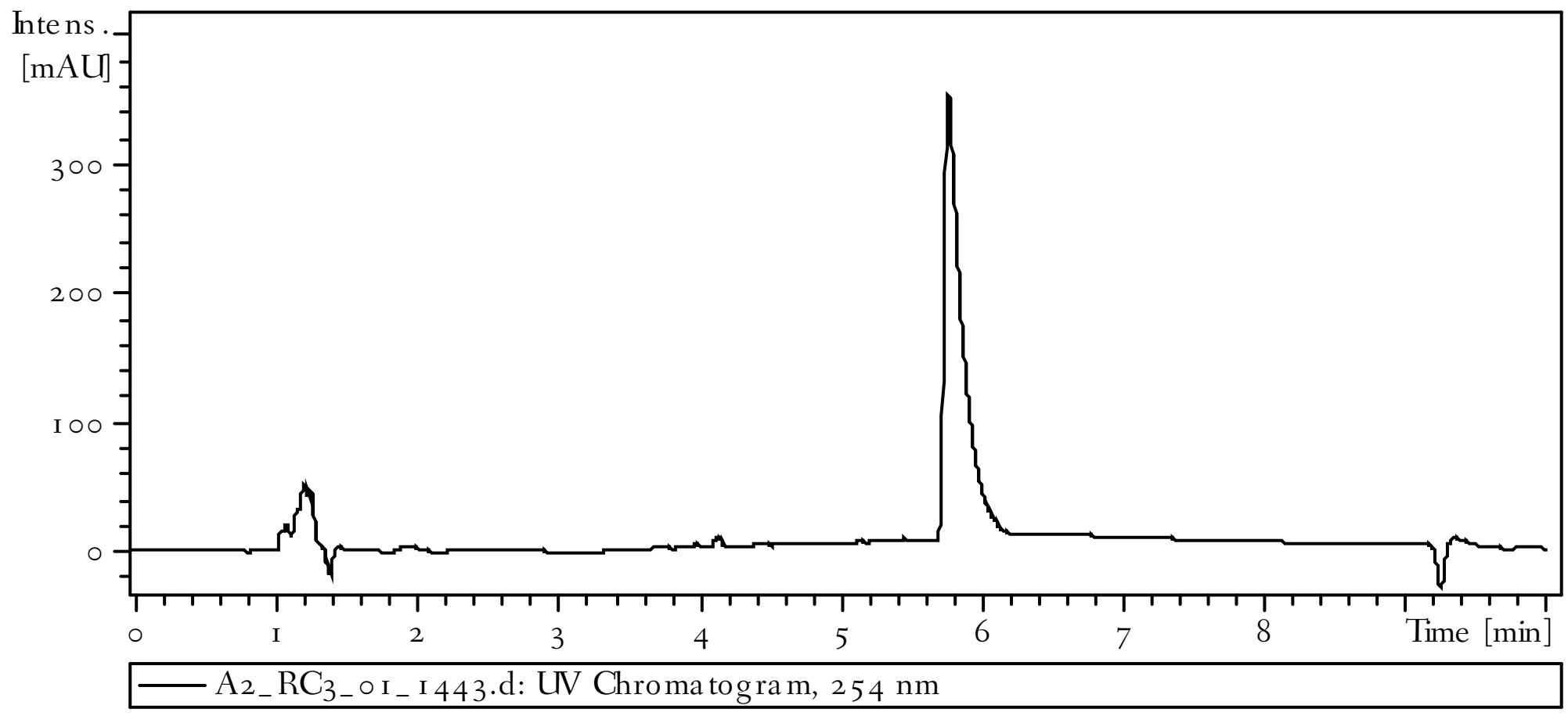

t_R:5.78 min, 97.7%

Fig. S40. HPLC chromatogram of compound **A2**.

A3

D:\Data\Data\2026\Athanassopoulos_26\UHPLC\06072026\A-3_RB5_01_1410.d
DAD (214.000000)

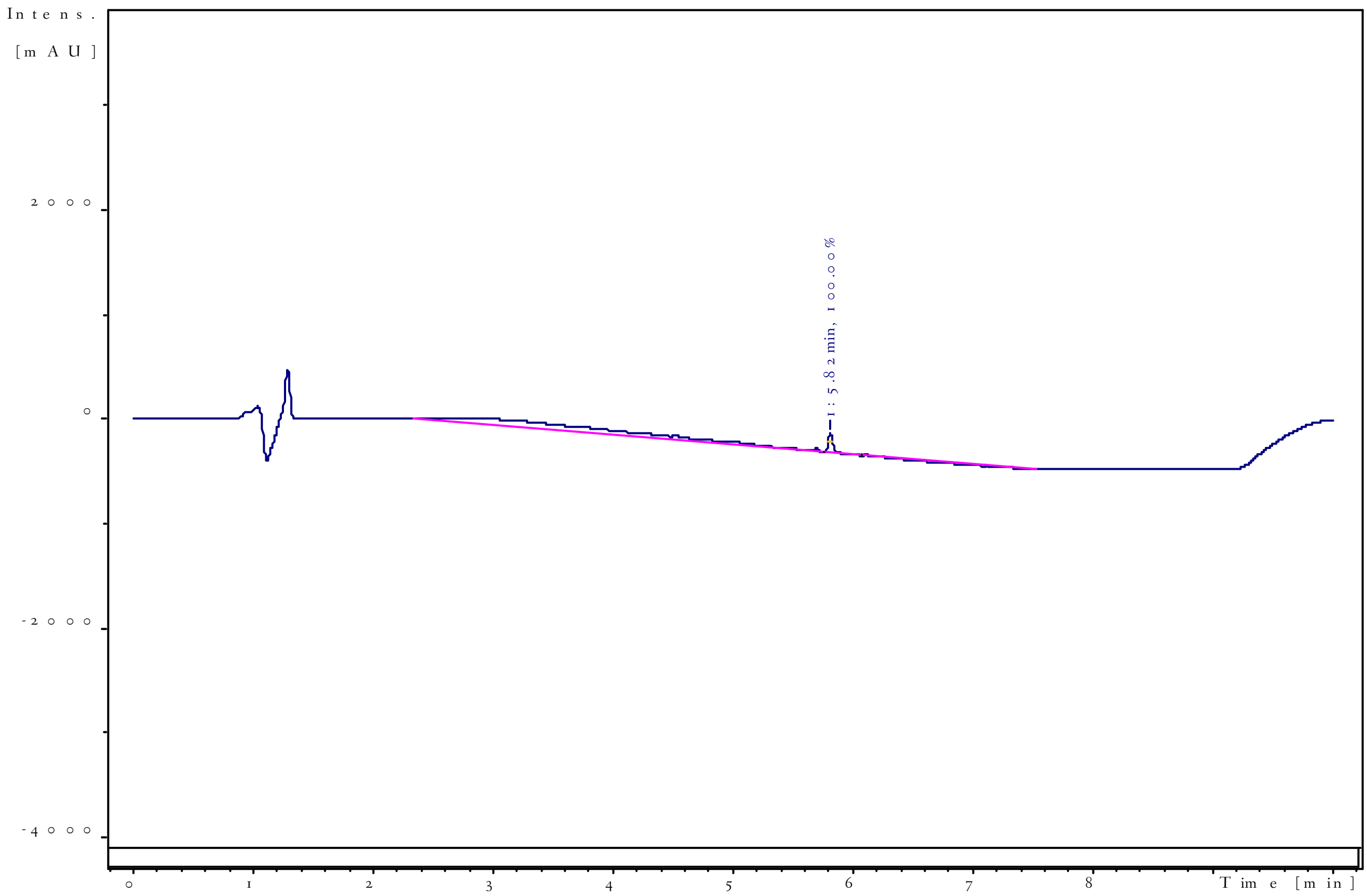

No	Time [min]	Intensity [mAu]	Integral [%]	Integral [mAU]
1	5.82	-150.45	100.00	1763.83

Fig. S41. HPLC chromatogram of compound **A3**.

A4

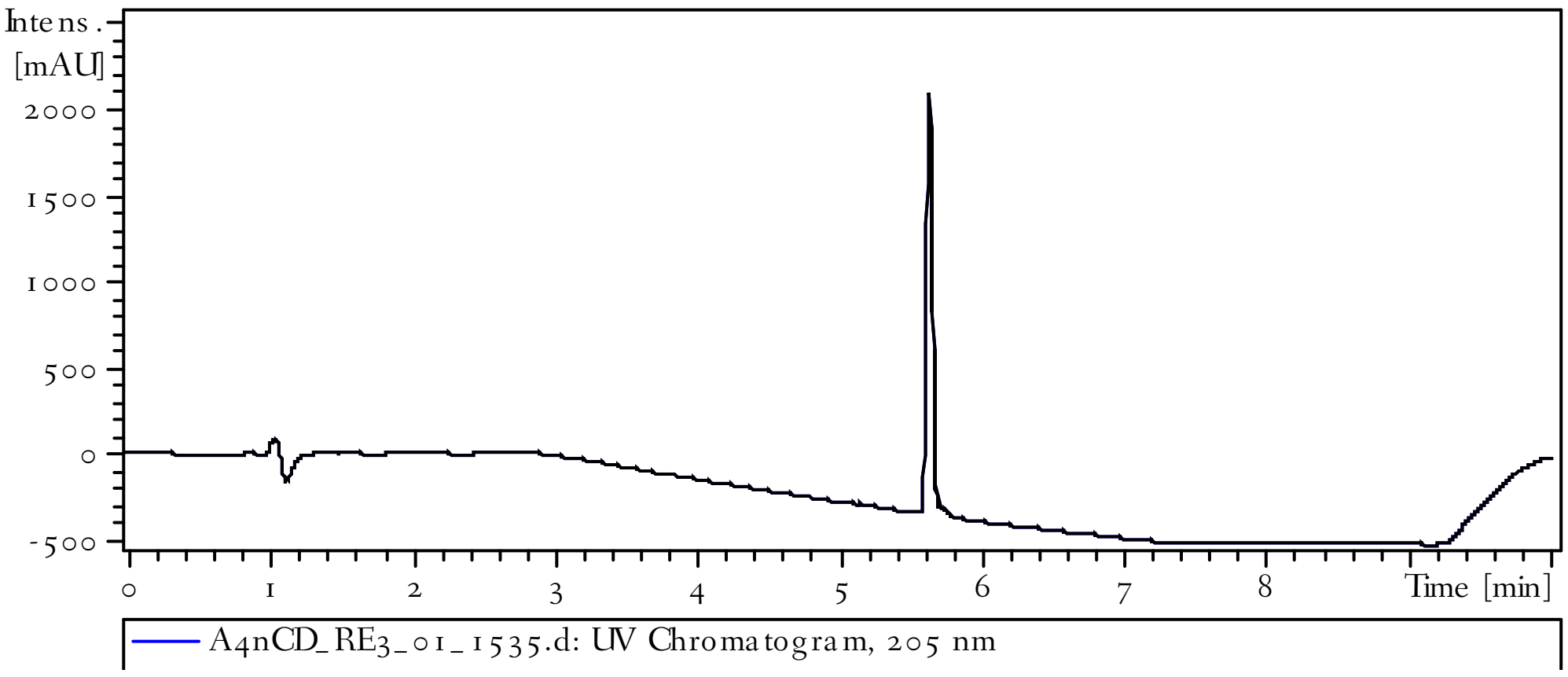

t_R:5.6 min, 99.1%

Fig. S42. HPLC chromatogram of compound **A4**.

A5

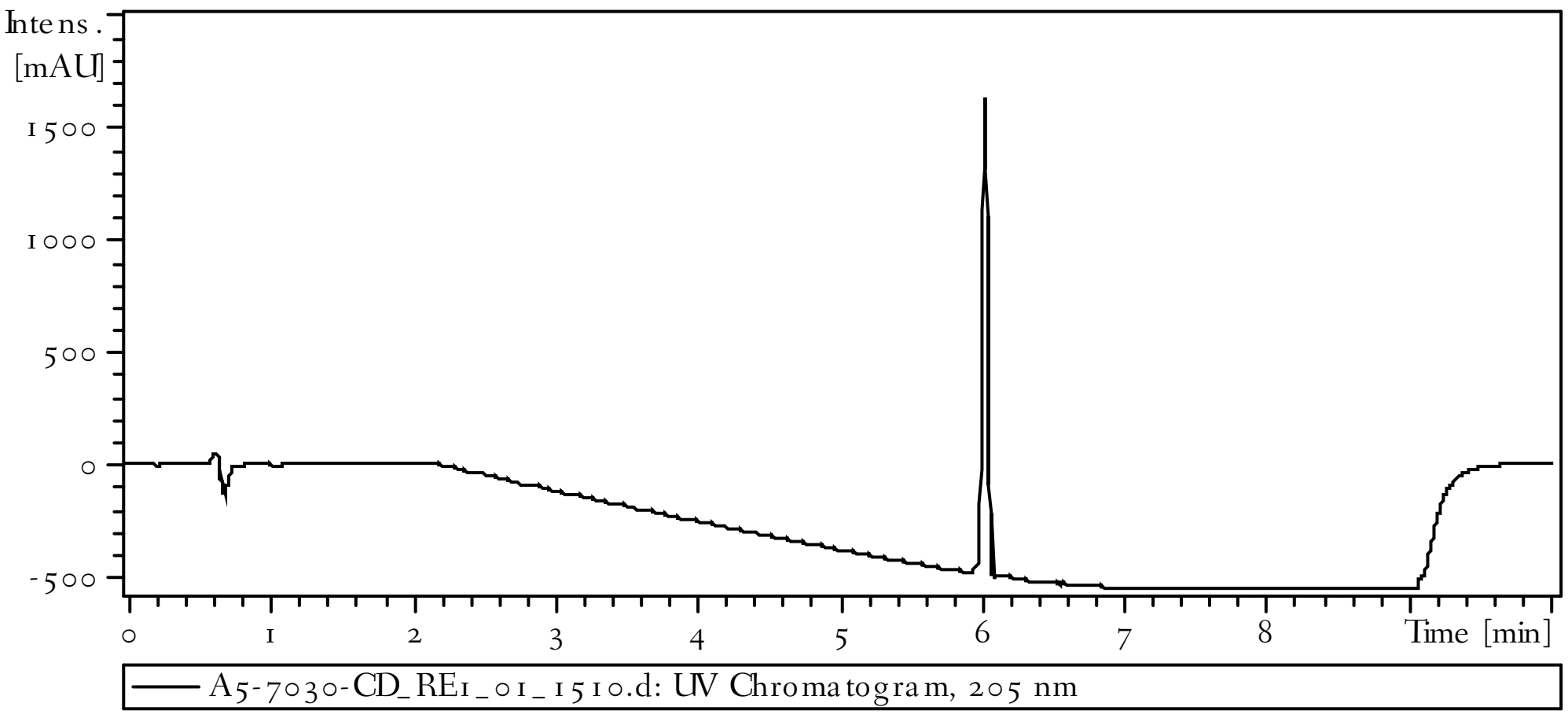

t_R:6.0 min, 99.8%

Fig. S43. HPLC chromatogram of compound **A5**.

A6

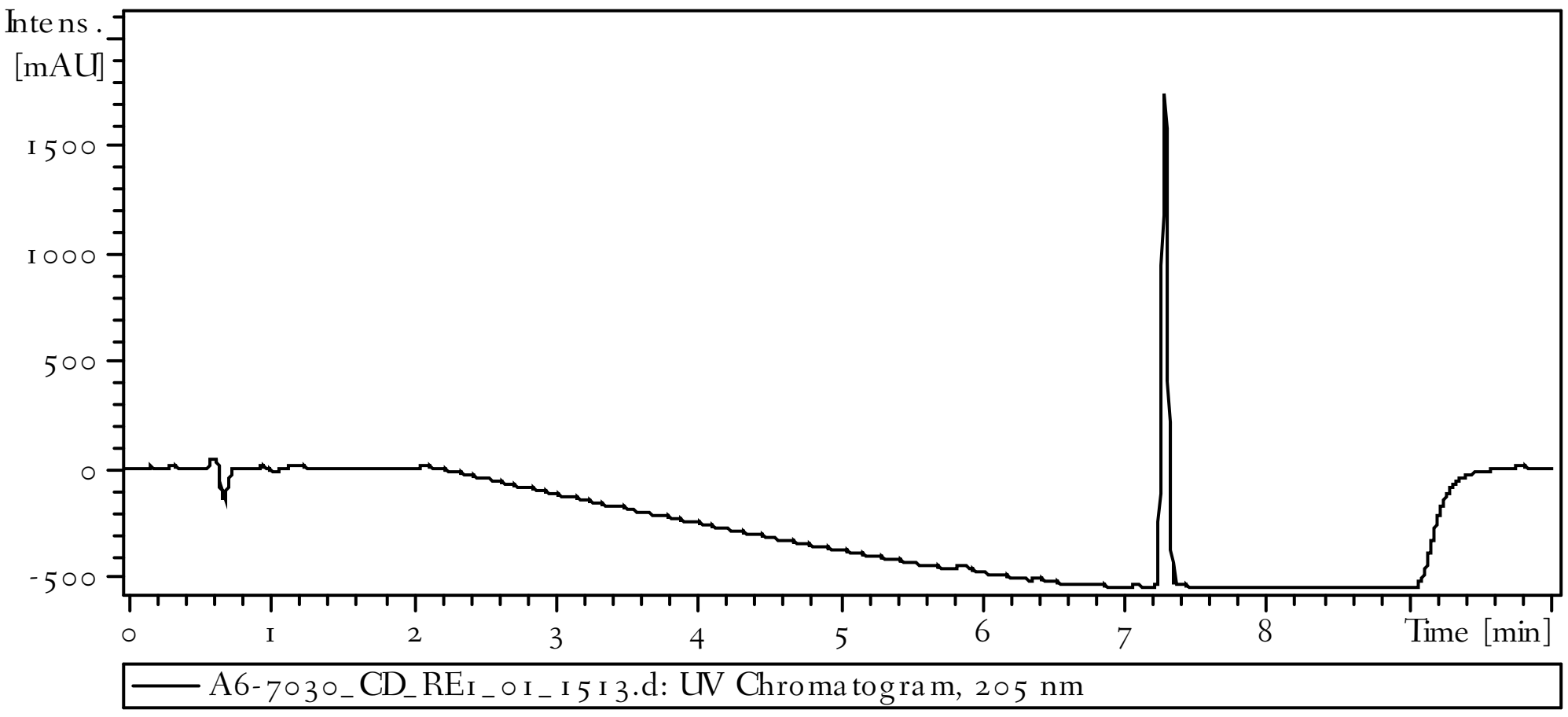

t_R: 7.3min, 98.1%

Fig. S44. HPLC chromatogram of compound **A6**.

A7

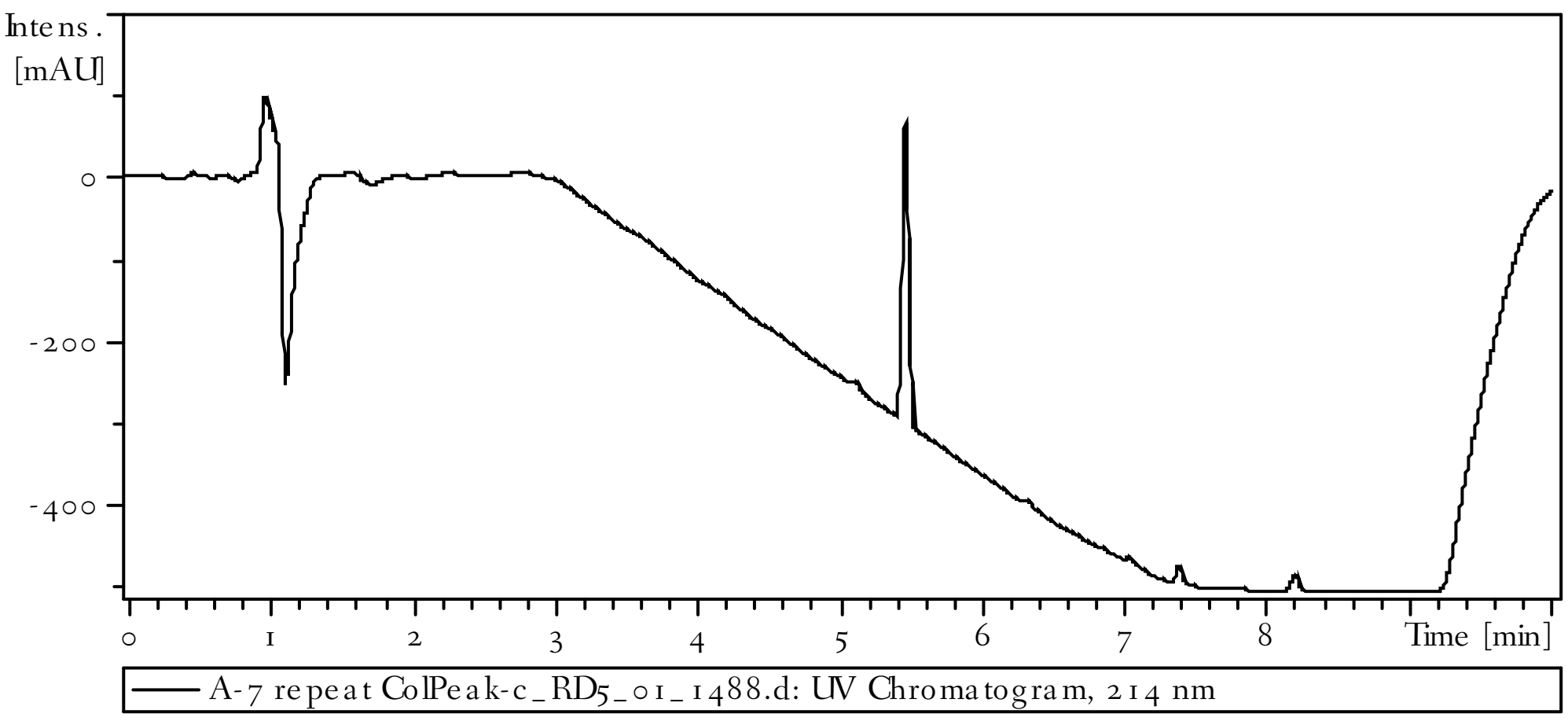

t_R:5.5 min, 95.1%

Fig. S45. HPLC chromatogram of compound **A7**.

A8

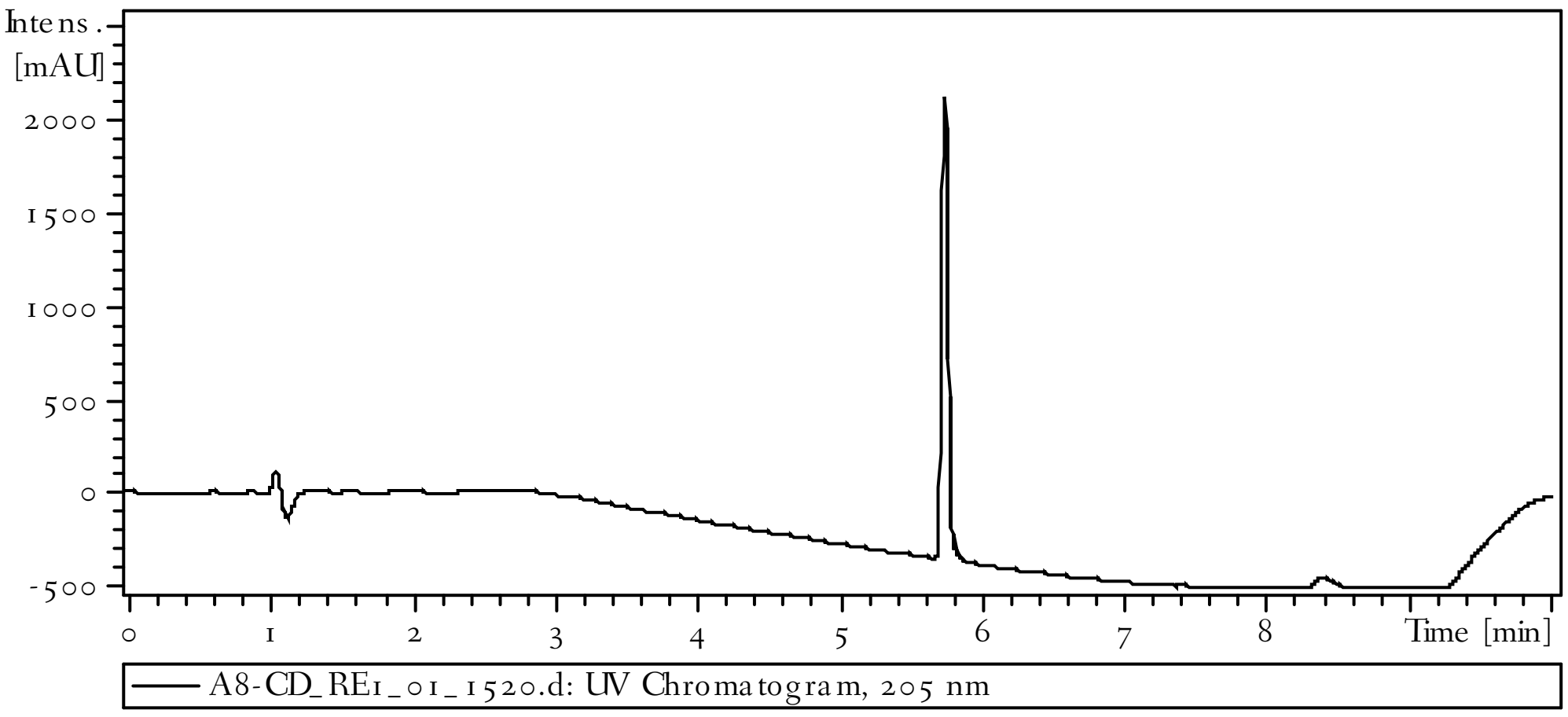

t_R:5.7 min, 95.6%

Fig. S46. HPLC chromatogram of compound **A8**.

A10

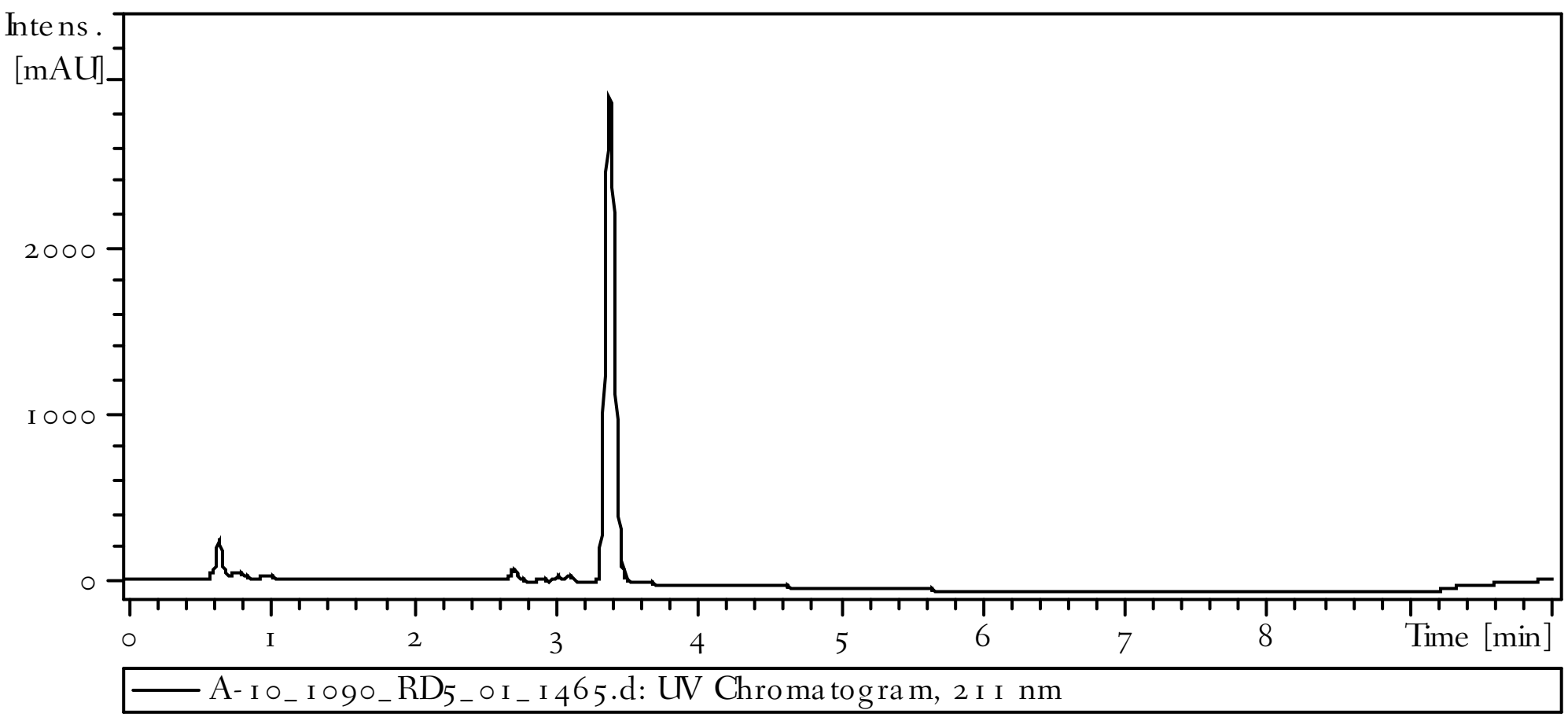

t_R:3.41 min, 96.62%

Fig. S47. HPLC chromatogram of compound **A10**.

A11

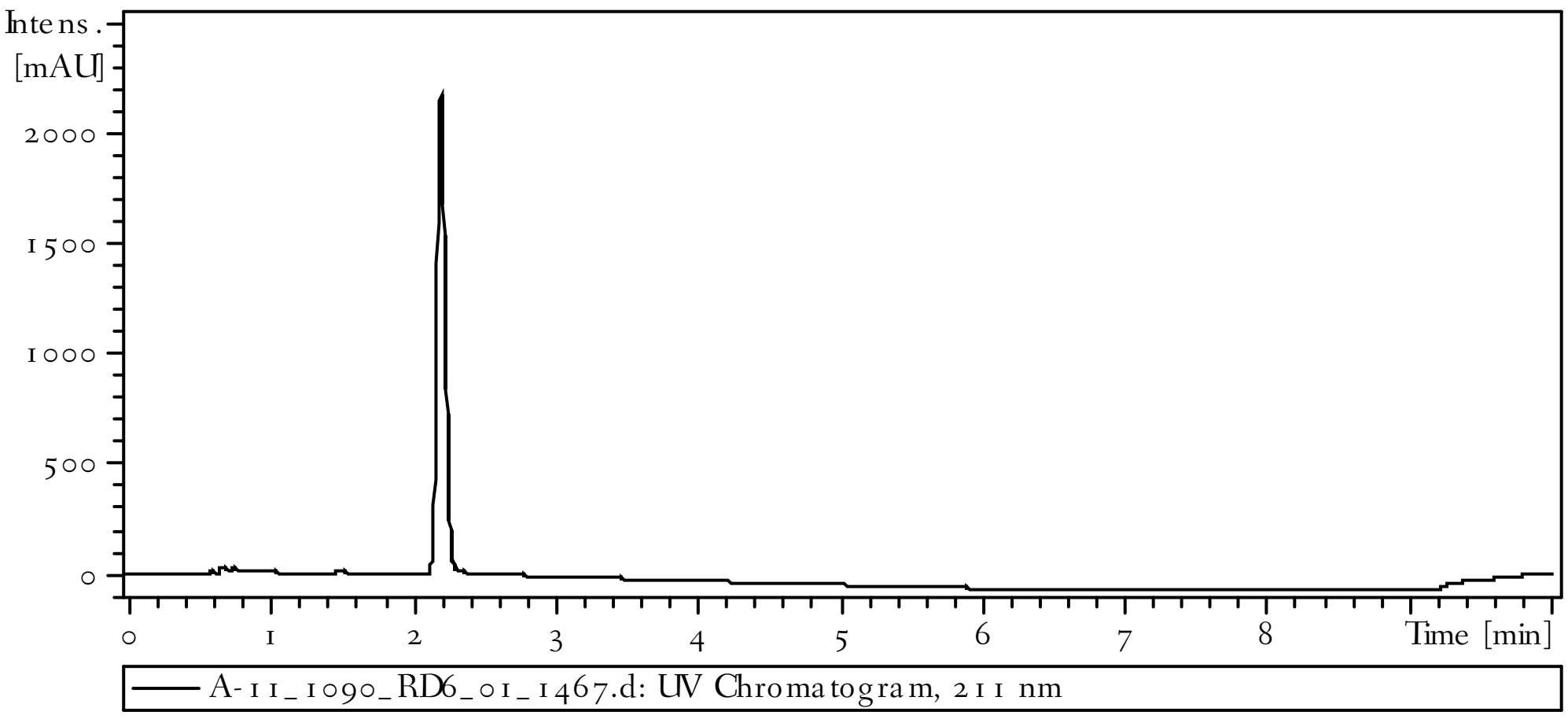

t_R:2.02 min, 99.9%

Fig. S48. HPLC chromatogram of compound **A11**.

A12

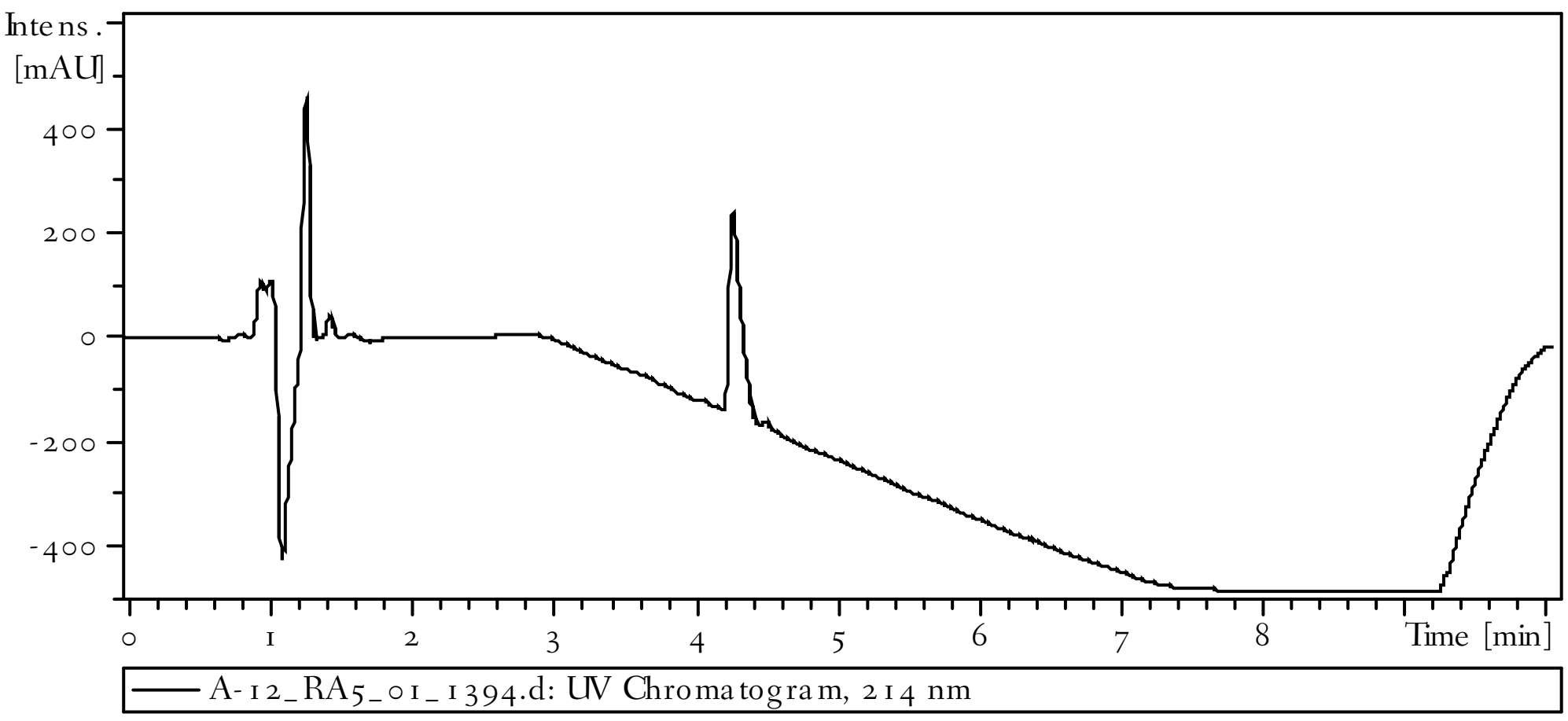

t_R: 4.3 min, 98.16%

Fig. S49. HPLC chromatogram of compound **A12**.

A13

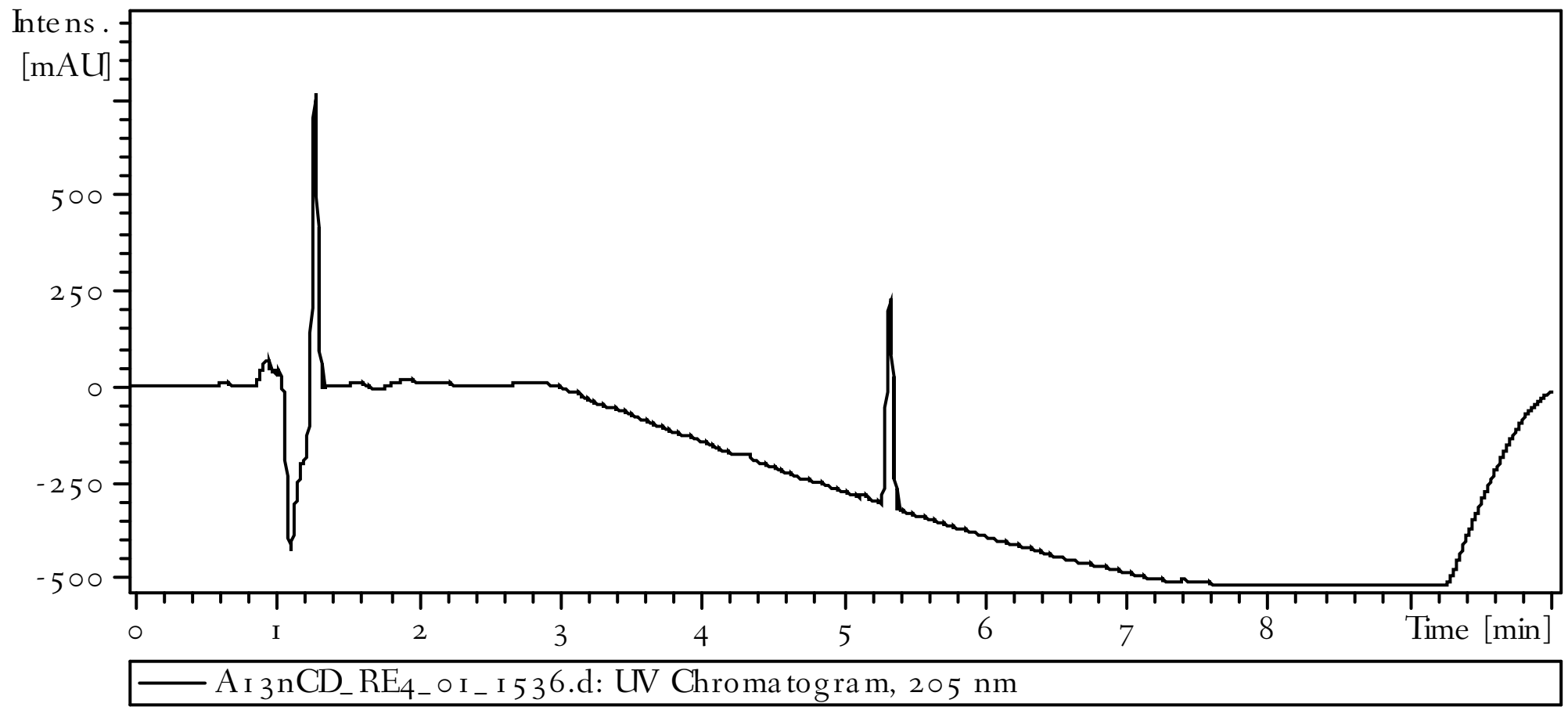

t_R:5.25 min, 98.2%

Fig. S50. HPLC chromatogram of compound **A13**.

A14

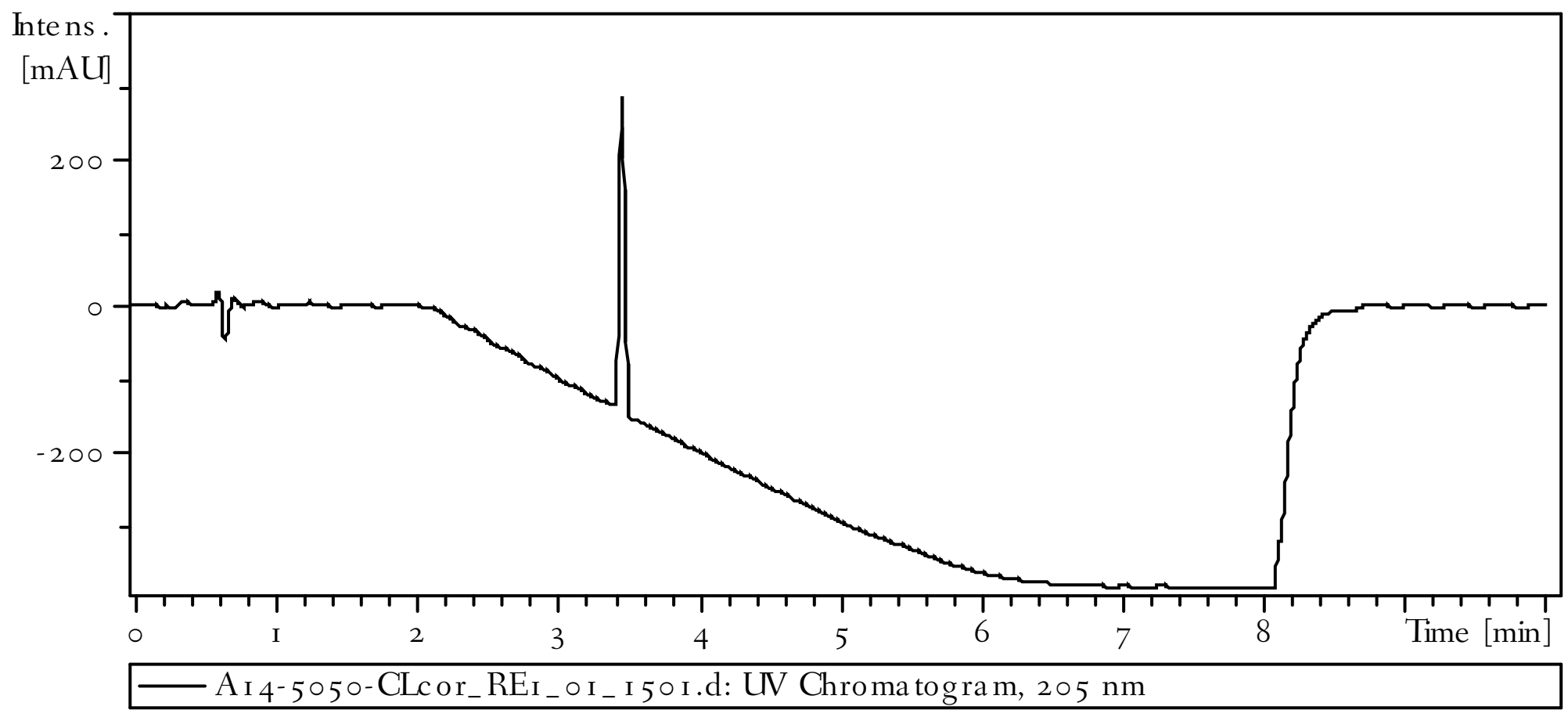

t_R:3.4 min, 95.3%

Fig. S51. HPLC chromatogram of compound **A14**.

A15

D:\Data\Data\2026\Athanassopoulos_26\UHPLC\06072026\A-15_RA7_01_1398

DAD (214.000000)

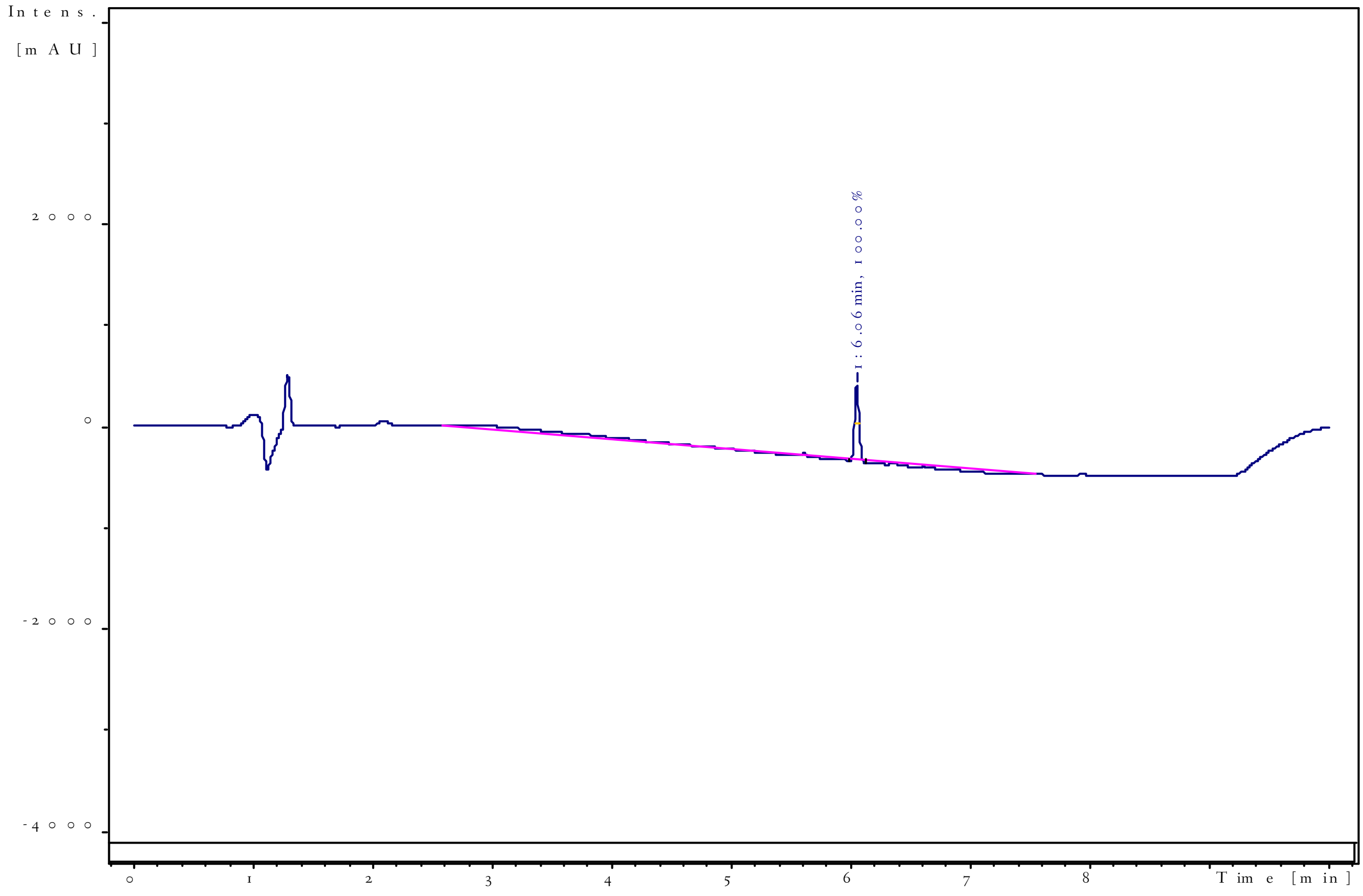

No	Time [min]	Intensity [mAu]	Integral [%]	Integral [mAU]
1	6.06	400.09	100.00	9460.60

Fig. S52. HPLC chromatogram of compound **A15**.

A16

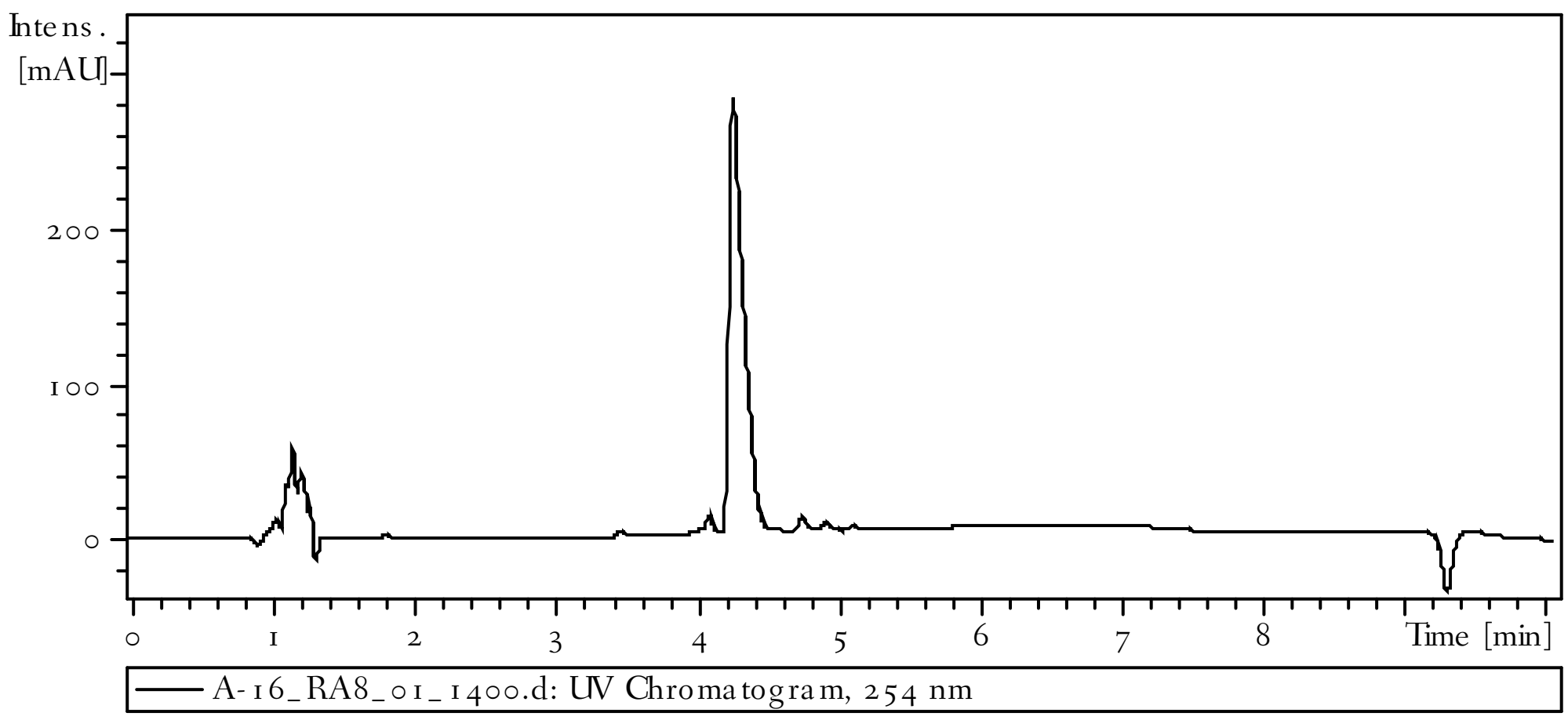

t_R:4.27 min, 95.12%

Fig. S53. HPLC chromatogram of compound **A16**.

A17

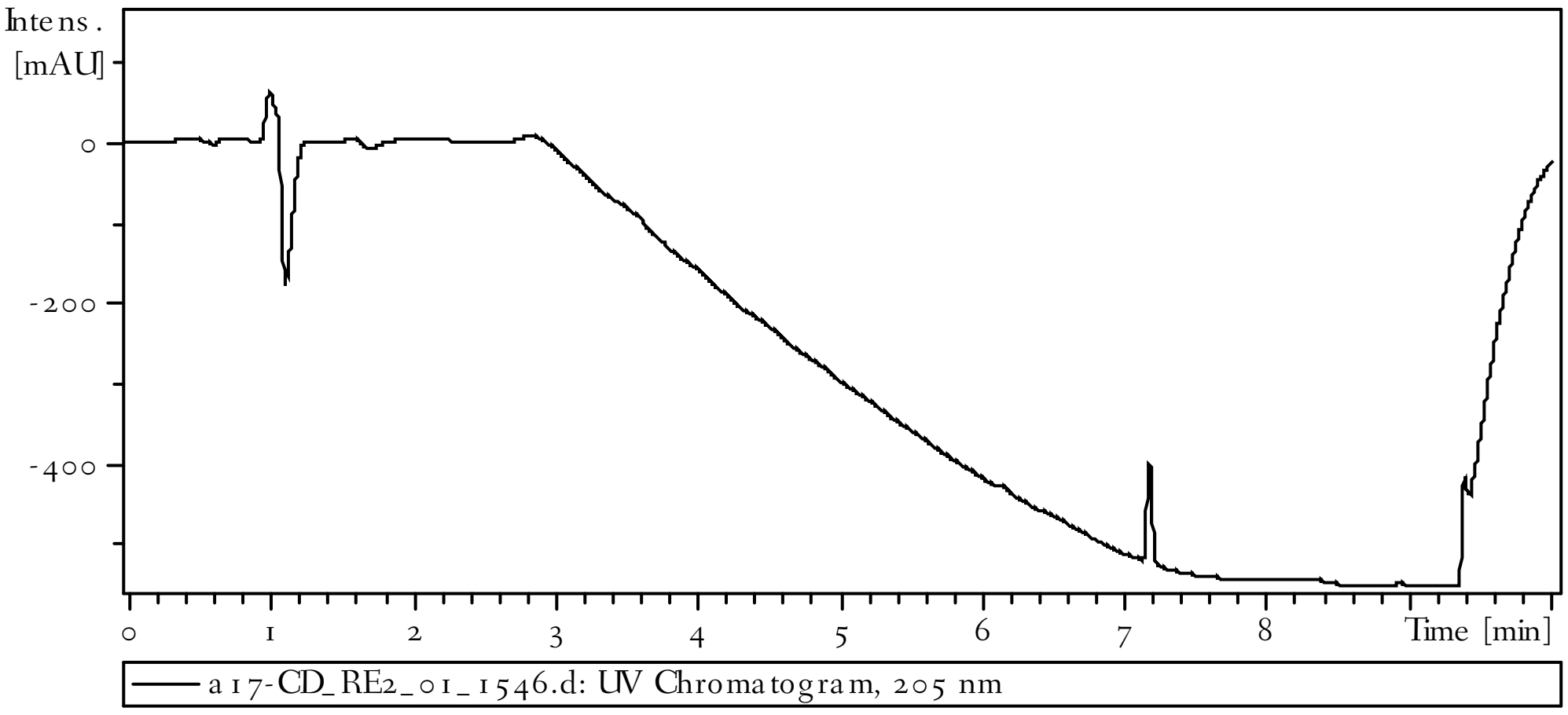

t_R: 7.2 min, 96.1%

Fig. S54. HPLC chromatogram of compound **A17**.

A18

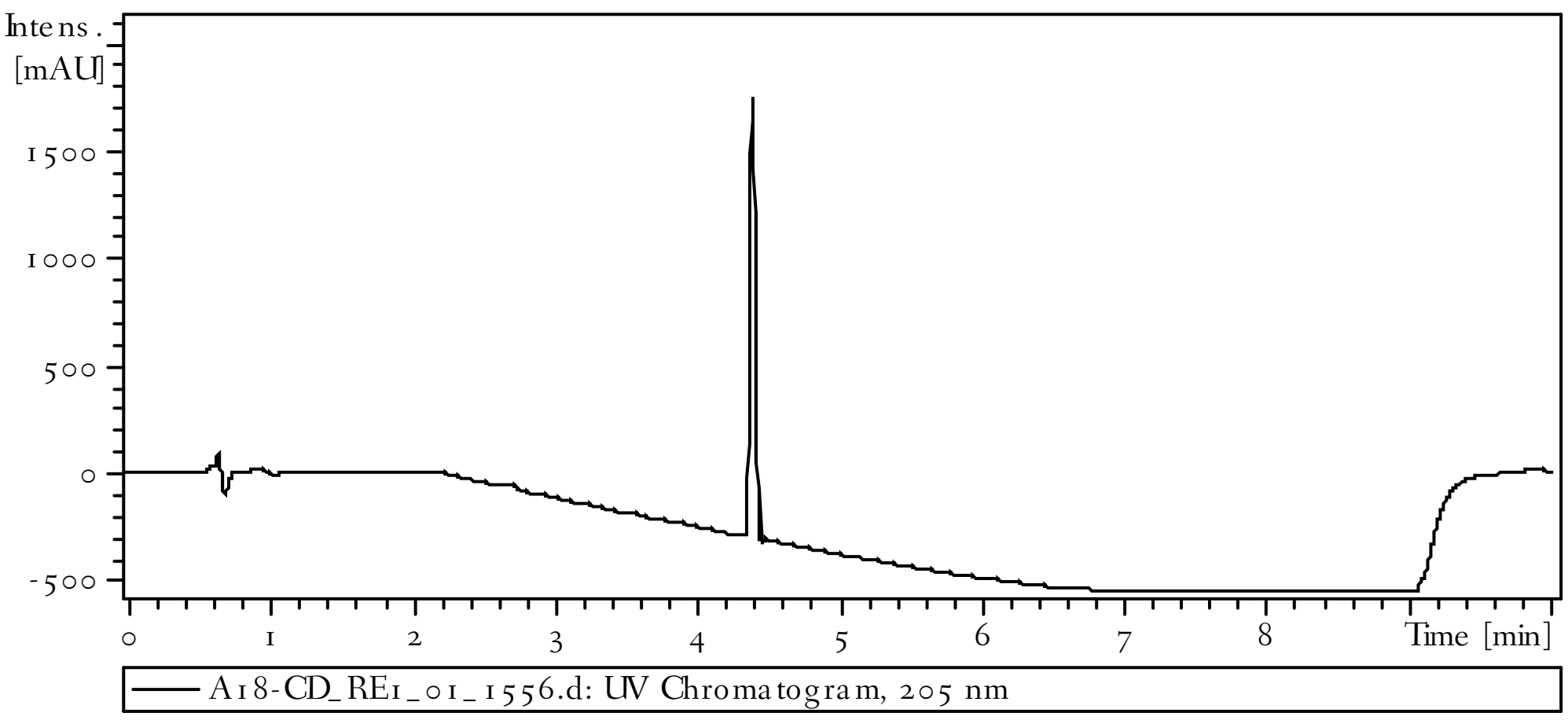

t_R:4.4 min, 99.5%

Fig. S55. HPLC chromatogram of compound **A18**.

A19

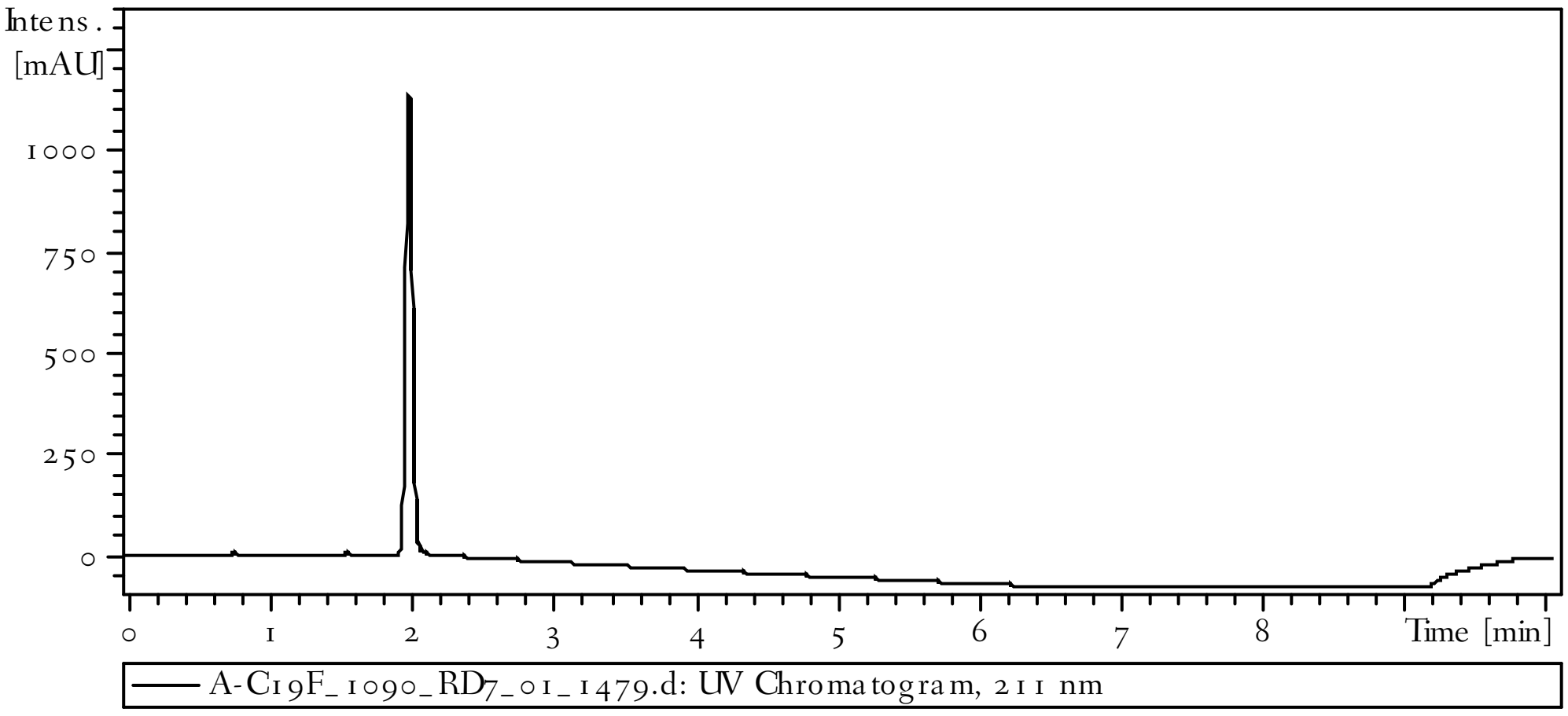

t_R:2.01 min, 98.15%

Fig. S56. HPLC chromatogram of compound **A19**.

A20

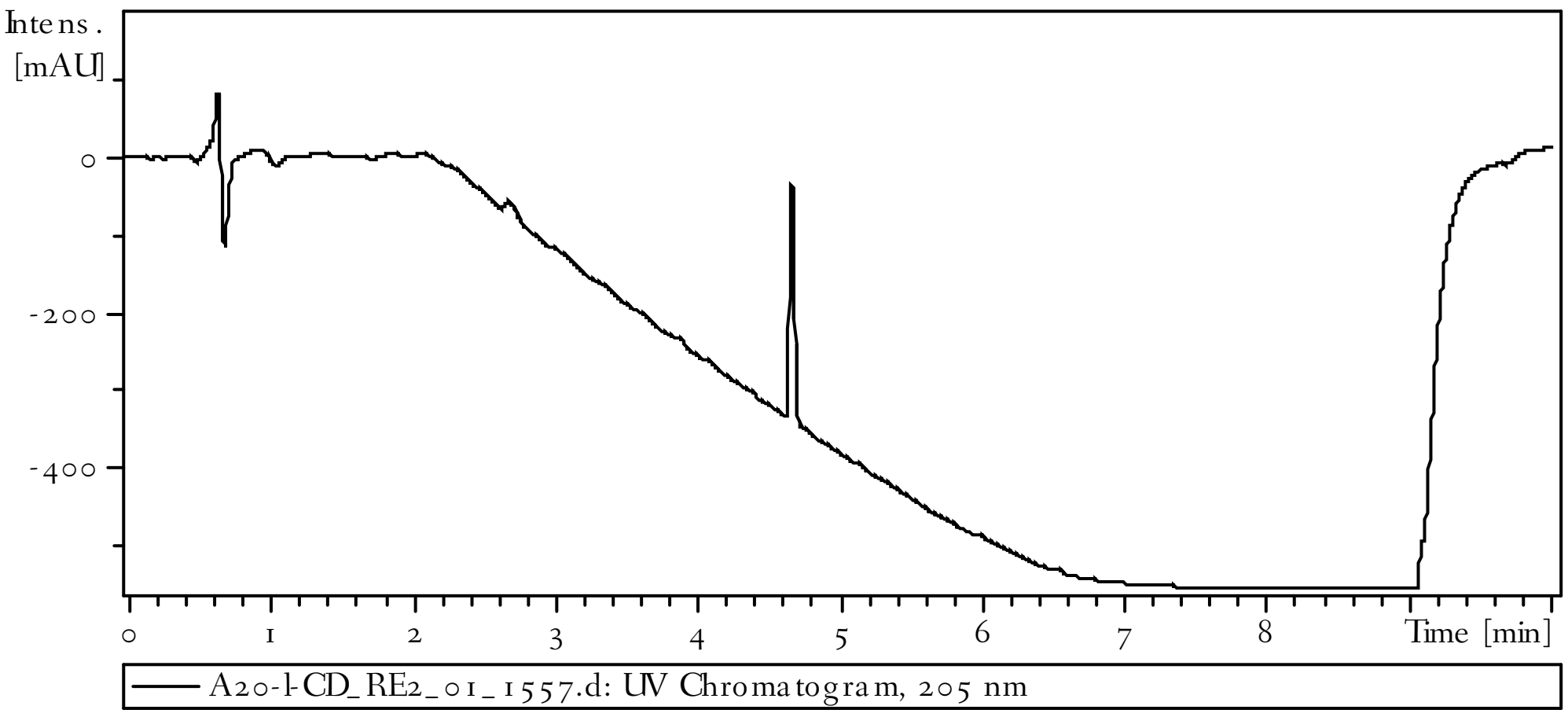

t_R:4.7 min, 95.6%

Fig. S57. HPLC chromatogram of compound **A20**.

Molecular modeling details

The following calculations aim to determine the electron-donor and polarizability properties of some of the fragments present in various substituents of the fragment referred to as "moiety 2" in the main text.

Table S2. Calculated properties for fragments commonly found in the artemisinin derivative series. Computational details for main text Table 1.

properties	ε(HOMO) /eV	ε(LUMO) /eV	E(N) /Hartree [(a)]	E(RC) /Hartree [(b)]
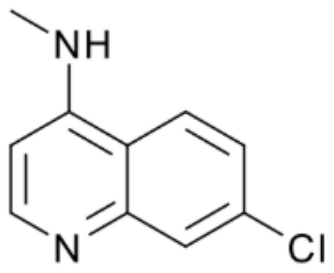	-7.096	-0.591	-956.069101	-955.789518
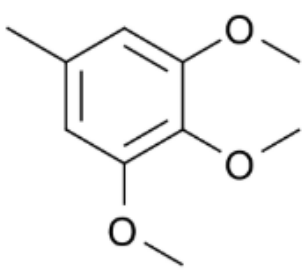	-7.399	-0.747	-614.990302	-614.700197
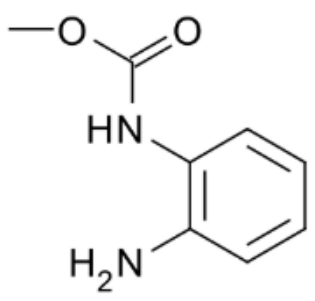	-7.414	0.488	-570.760499	-570.470708
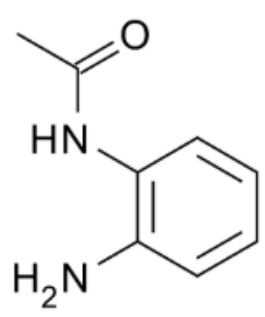	-7.423	0.397	-495.529661	-495.235504
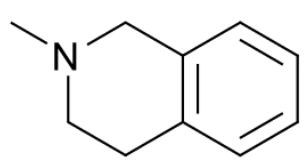	-7.622	0.422	-443.493424	-443.194491

a) Computed total electronic energy of the neutral molecule. b) Total electronic energy of the radical cation at the equilibrium geometry of the neutral.

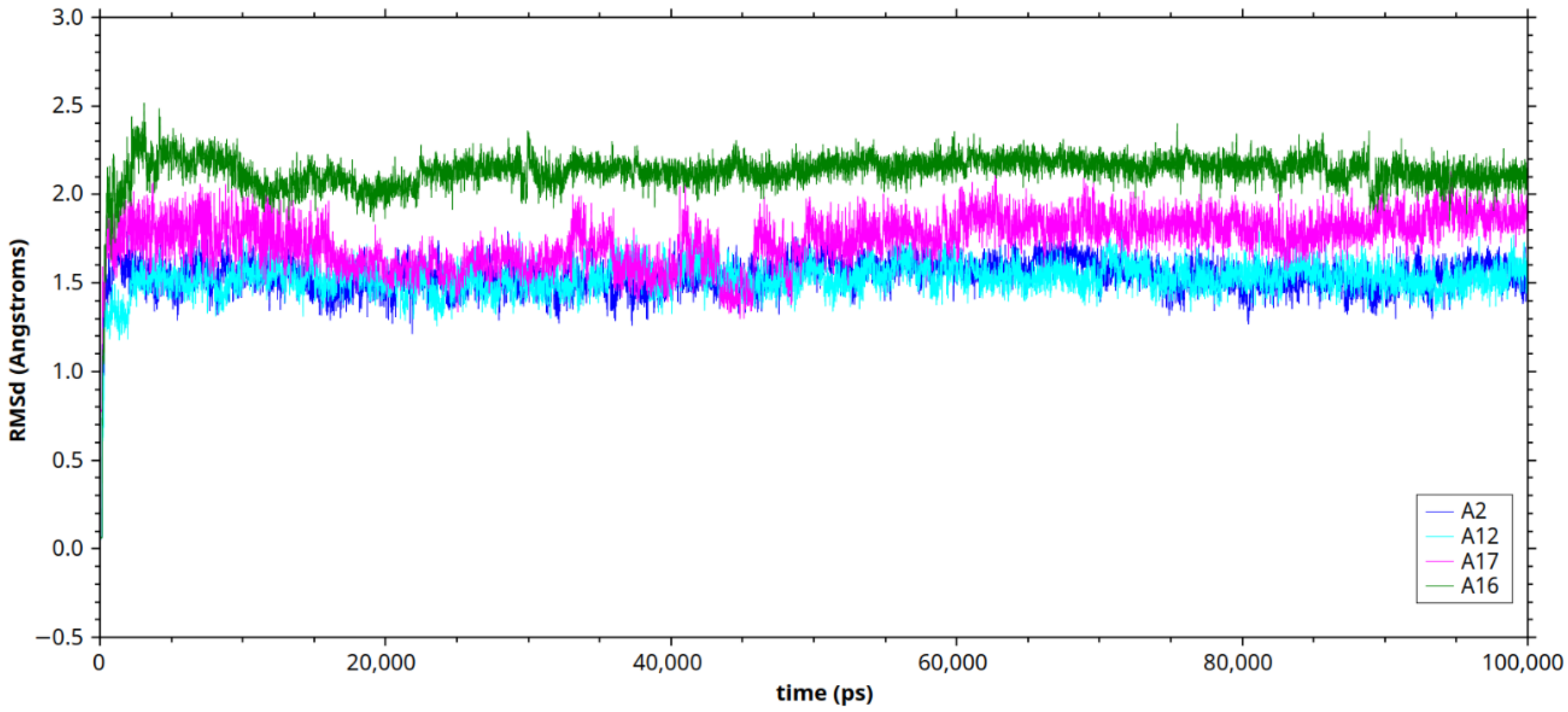

Fig. S58. Examples of typical RMSd 's obtained.

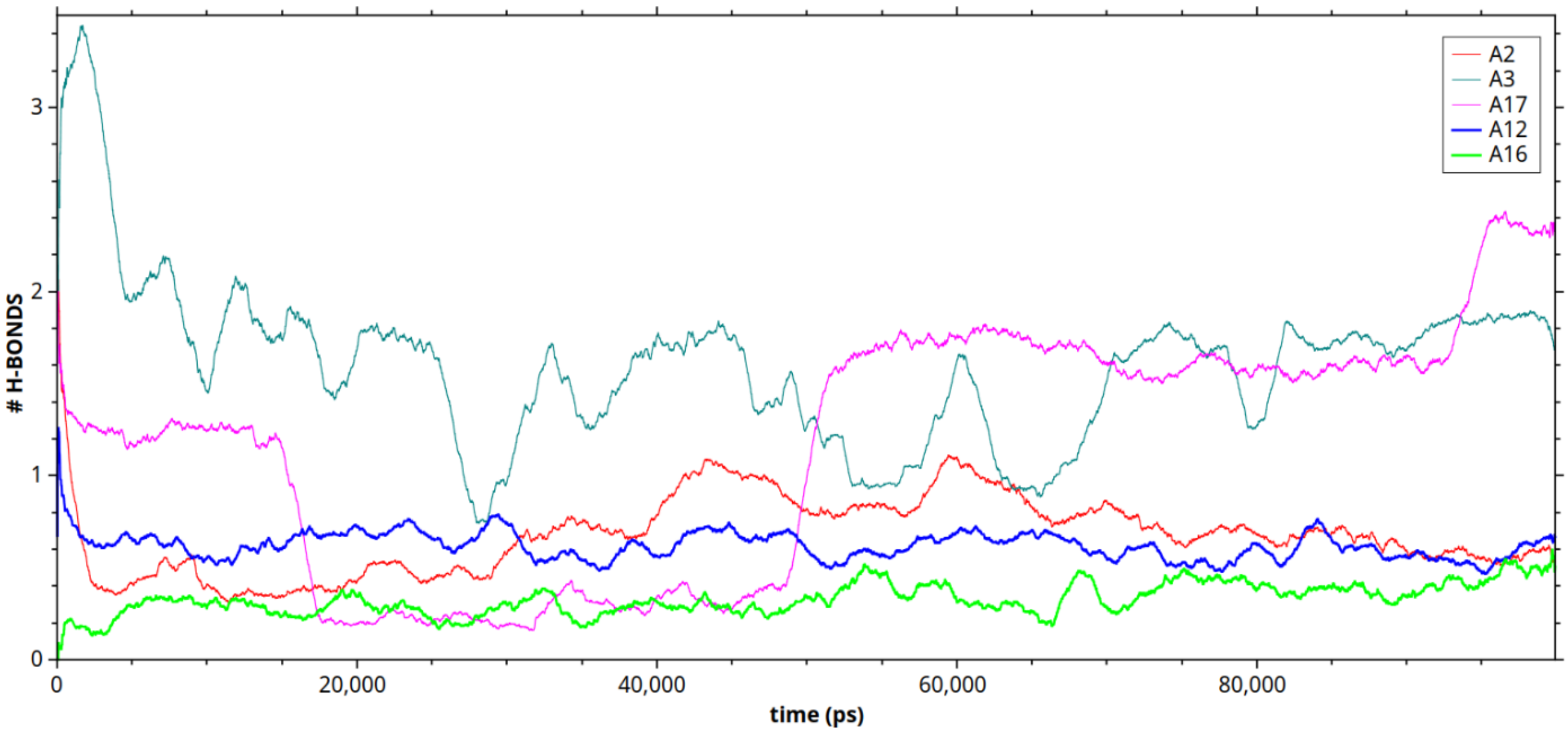

Fig. S59. Total number of hydrogen bonds (H-bonds) plotted as running average over 250 ps. No clear preference for H-bond formation is observed for the most potent compounds such as compound **A12** or compound **A16**.

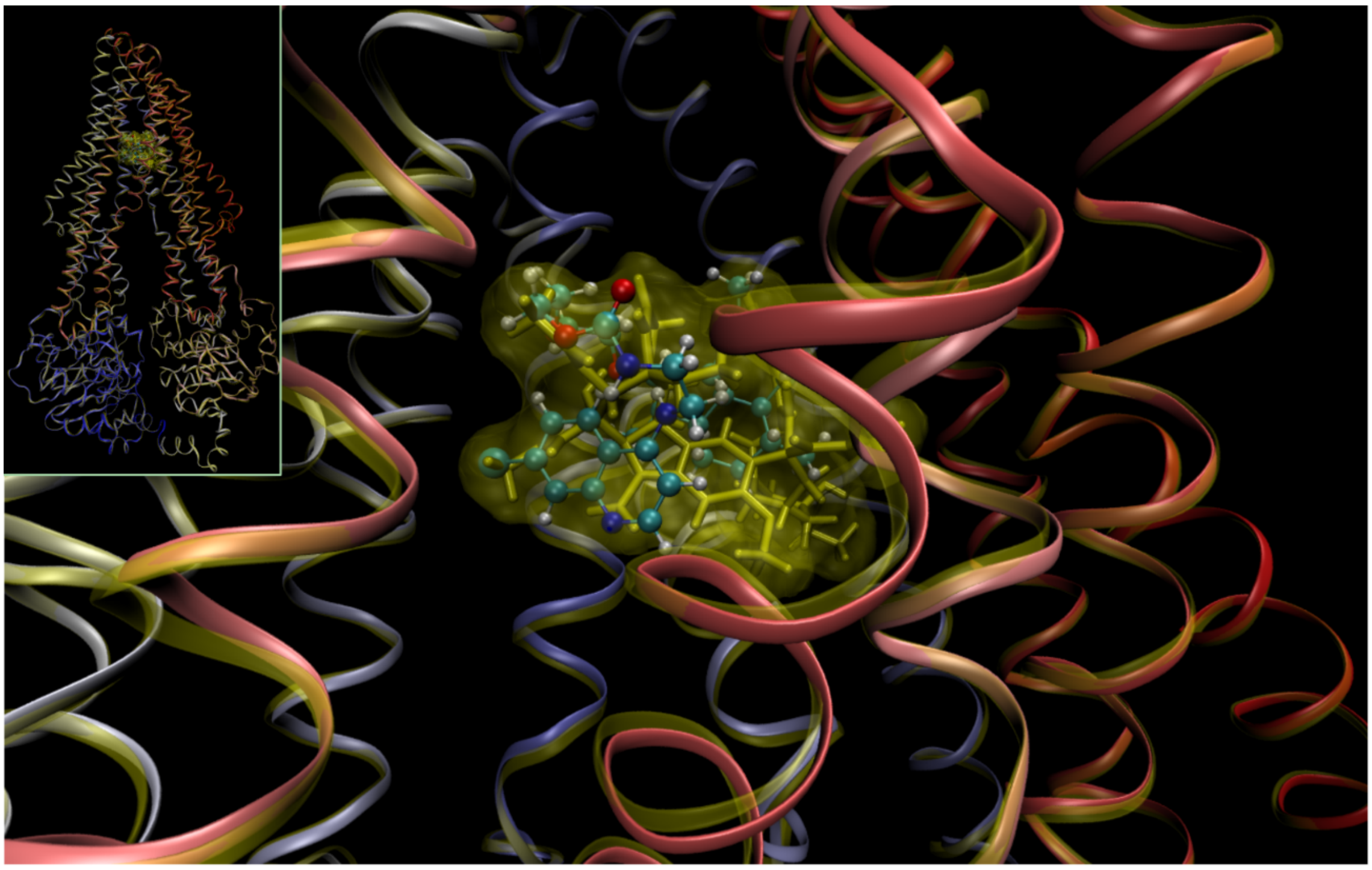

Fig. S60. Superimposition of compound **A16** (CPK representation, with the backbone colored according to sequence from red to blue) and vincristine (yellow licorice representation). The structure corresponds to the most populated cluster identified from cluster analyses of the 100ns molecular dynamics (MD) simulation. Throughout the simulation, vincristine remained in the experimental binding pose determined by Cryo-EM. The inset in the upper left corner shows the overall structure.

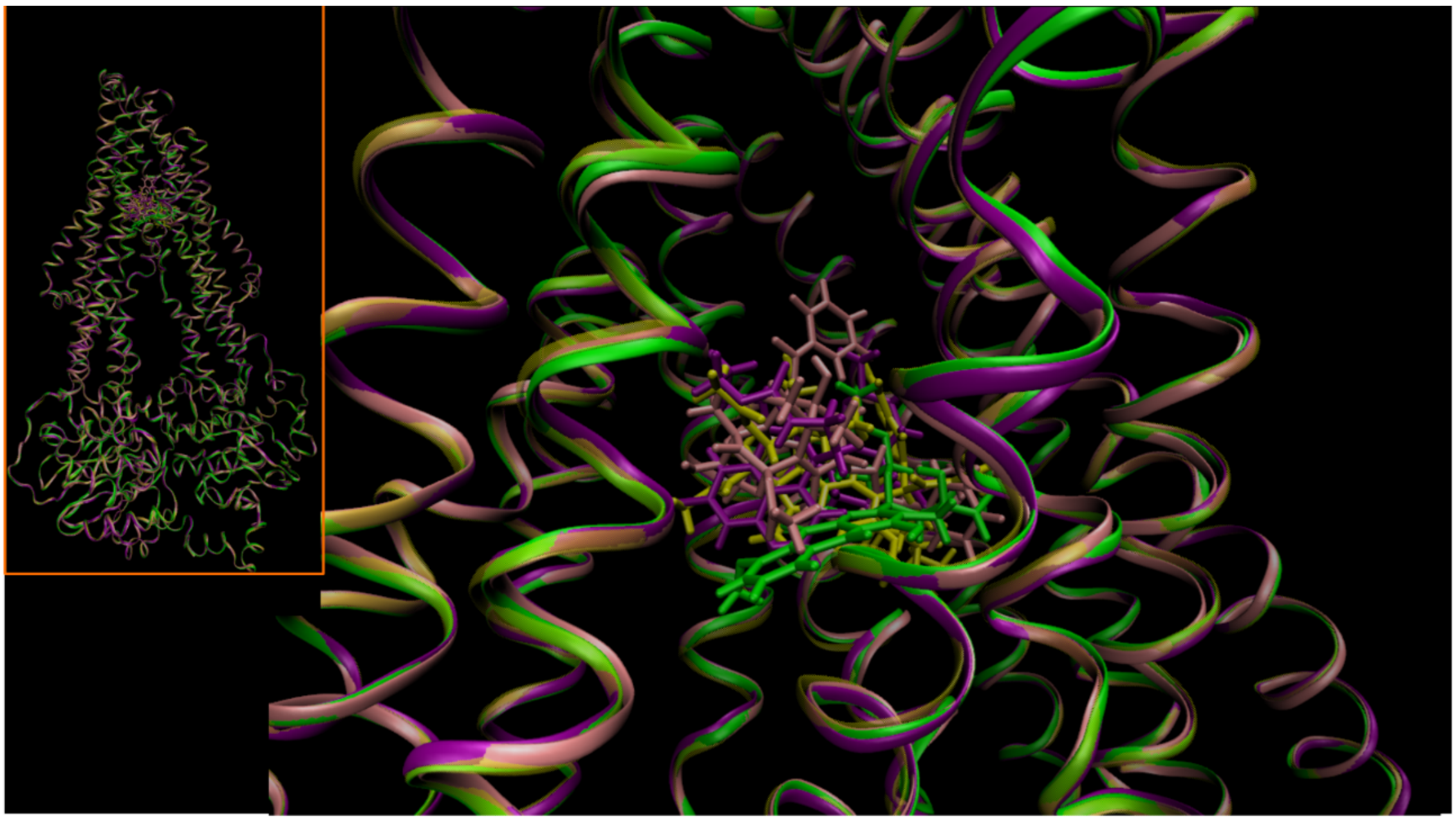

Fig. S61. Superimposition of the representative cluster structures obtained from the molecular dynamics (MD) simulations of compounds **A16** (purple), taxol (pink), doxorubicin (green) and vincristine (yellow). The representation is the same as that used in Fig. S67.

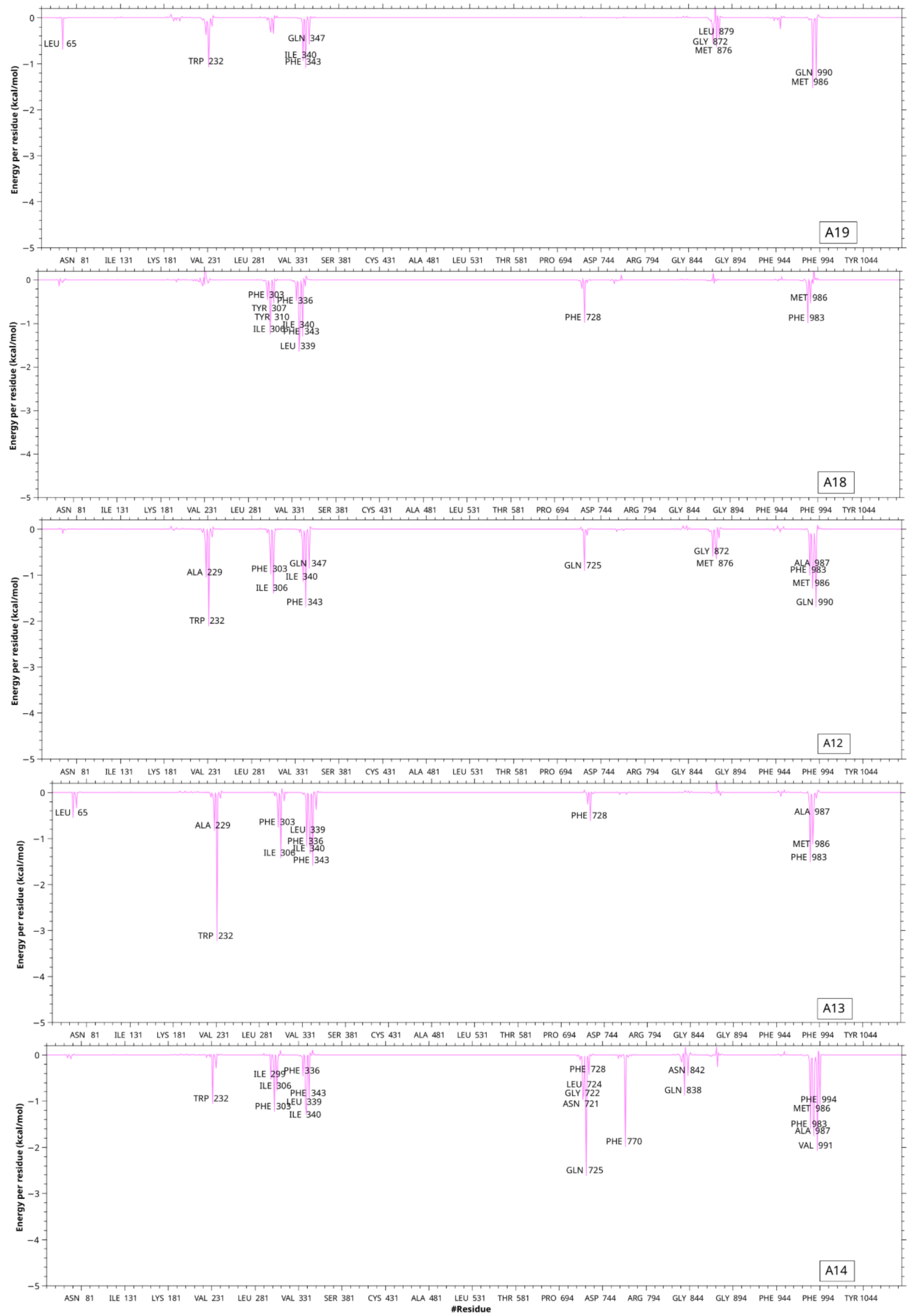

Fig. S62. Energy contributions in terms of per residue decomposition for compounds **A19**, **A18**, **A12**, **A13** and **A14**. See also Fig. 6 in main text.

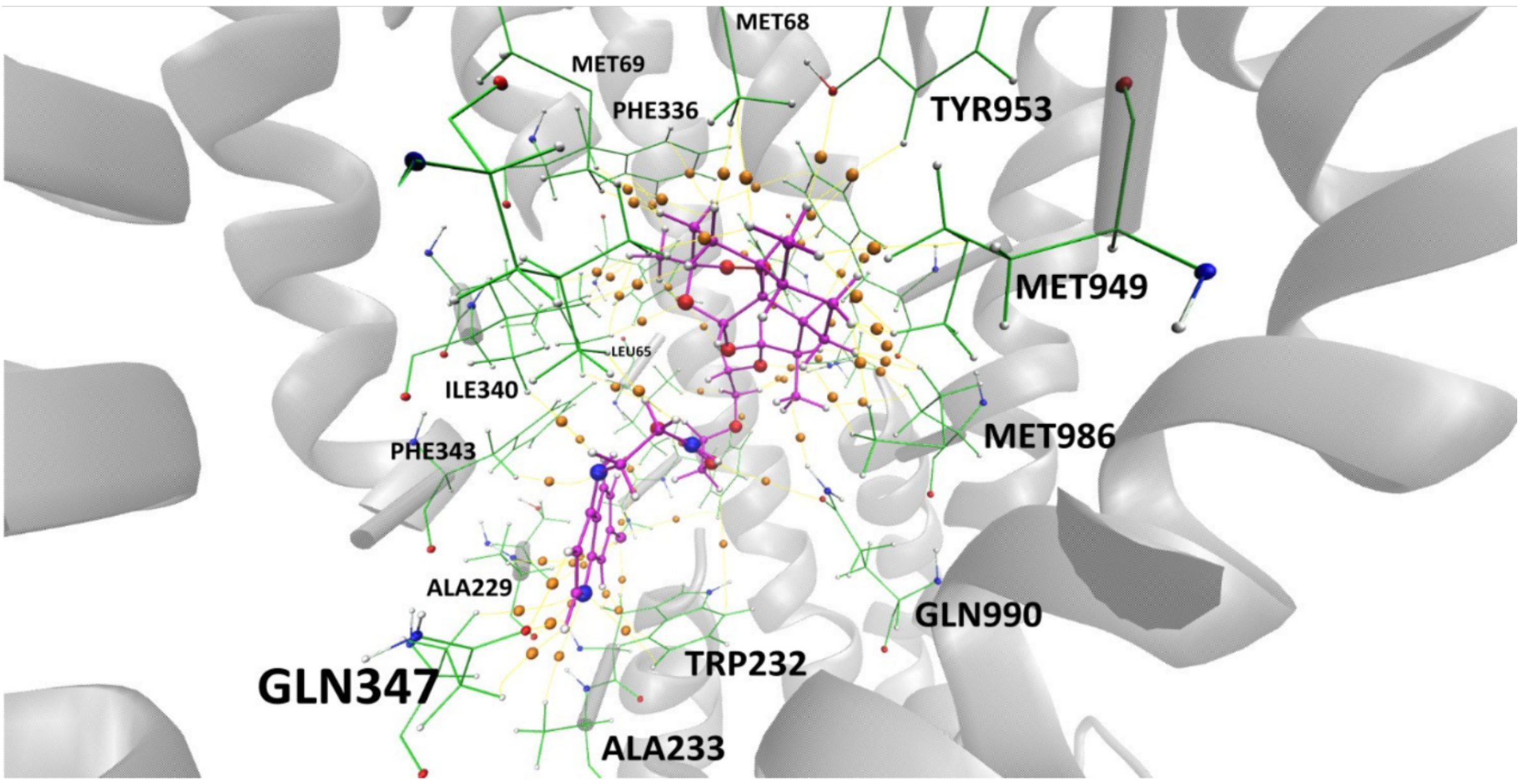

Fig. S63. QM/MM/QTAIM analysis of compound **A16**. Non-covalent interactions between P-gp residues (green) and compound **A16** (magenta) are shown. Bond paths are depicted in yellow and bond critical points (BCPs) are represented as small orange spheres.

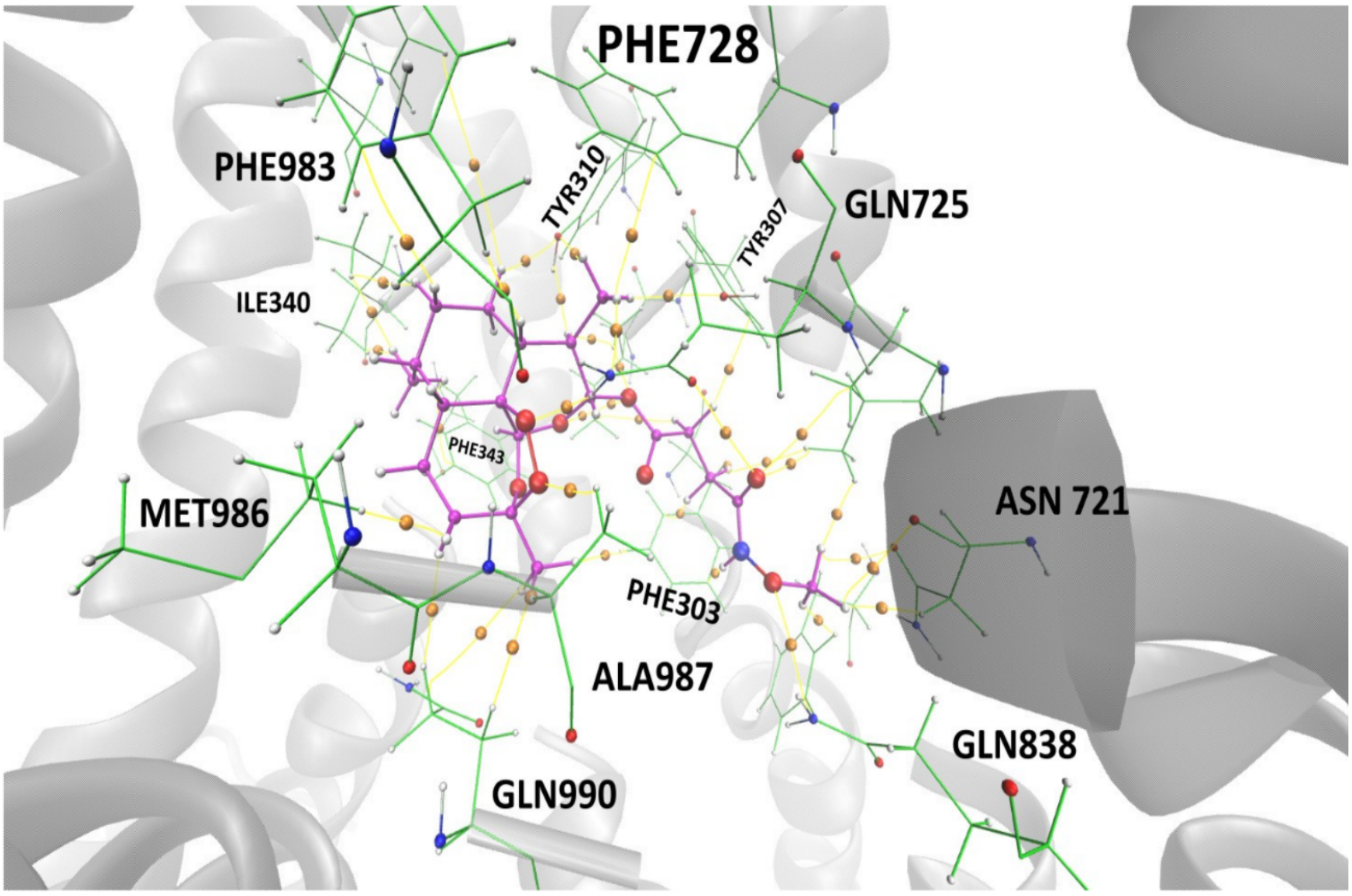

Fig. S64. QM/MM/QTAIM analysis for compound **A2**. The same color scheme as in Fig. S70 was used.

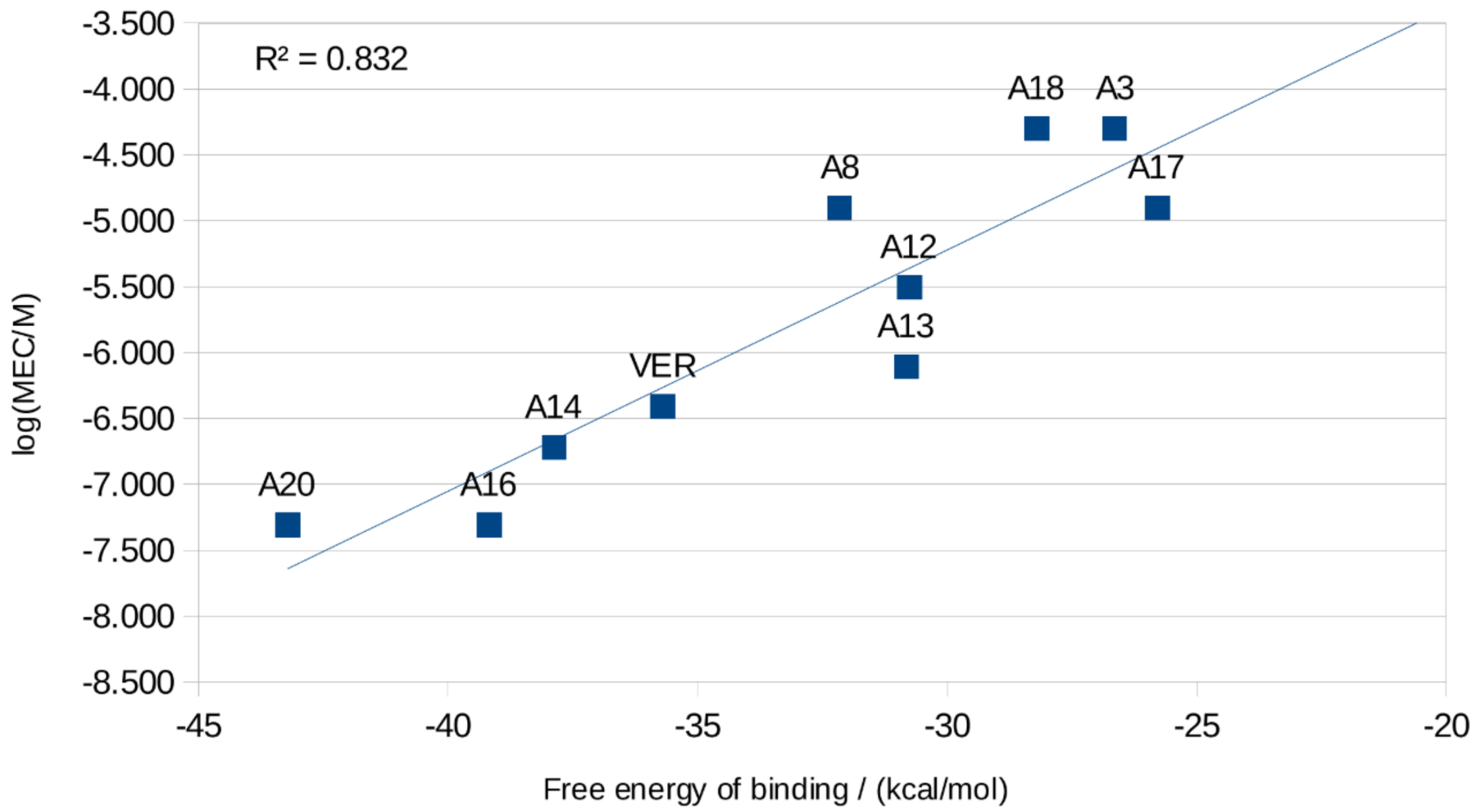

Fig. S65. Correlation between the calculated free energies of binding (ΔG°_b) and the experimental MEC's (VER=verapamil).
